# microRNA-33 deficiency in macrophages enhances autophagy, improves mitochondrial homeostasis, and protects against lung fibrosis

**DOI:** 10.1172/jci.insight.158100

**Published:** 2023-02-22

**Authors:** Farida Ahangari, Nathan L. Price, Shipra Malik, Maurizio Chioccioli, Thomas Bärnthaler, Taylor S. Adams, Jooyoung Kim, Sai Pallavi Pradeep, Shuizi Ding, Carlos Cosmos, Kadi-Ann S. Rose, John E. McDonough, Nachelle R. Aurelien, Gabriel Ibarra, Norihito Omote, Jonas C. Schupp, Giuseppe DeIuliis, Julian A. Villalba Nunez, Lokesh Sharma, Changwan Ryu, Charles S. Dela Cruz, Xinran Liu, Antje Prasse, Ivan Rosas, Raman Bahal, Carlos Fernández-Hernando, Naftali Kaminski

**Affiliations:** 1 Section of Pulmonary, Critical Care and Sleep Medicine, Yale University School of Medicine, New Haven, Connecticut, USA.; 2 Vascular Biology and Therapeutics Program, Yale Center for Molecular and System Metabolism, Department of Comparative Medicine, and Department of Pathology, Yale University School of Medicine, New Haven, Connecticut, USA.; 3 Experimental Gerontology Section, Translational Gerontology Branch, National Institute on Aging, NIH, Baltimore, Maryland, USA.; 4 Department of Pharmaceutical Sciences, School of Pharmacy, University of Connecticut, Storrs, Connecticut, USA.; 5 Division of Pharmacology, Otto Loewi Research Center, Medical University of Graz, Graz, Austria.; 6 Department of Internal Medicine, Weill Cornell Hospital Medicine, New York, New York, USA.; 7 Life Span Medical Group, Department of Internal Medicine, Rhode Island Hospital, Providence, Rhode Island, USA.; 8 Division of Pulmonary and Critical Care Medicine, Brigham and Women’s Hospital, Harvard Medical School, Boston, Massachusetts, USA.; 9 Center for Cellular and Molecular Imaging (CCMI), Department of Cell Biology, Yale School of Medicine, New Haven, Connecticut, USA.; 10 Department of Pneumology, University of Hannover, Fraunhofer Institute for Toxicology and Experimental Medicine, Hannover, Germany.; 11 Department of Medicine, Baylor College of Medicine, Houston, Texas, USA.

**Keywords:** Metabolism, Pulmonology, Autophagy, Fatty acid oxidation, Fibrosis

## Abstract

Idiopathic pulmonary fibrosis (IPF) is a progressive and ultimately fatal disease. Recent findings have shown a marked metabolic reprogramming associated with changes in mitochondrial homeostasis and autophagy during pulmonary fibrosis. The microRNA-33 (miR-33) family of microRNAs (miRNAs) encoded within the introns of sterol regulatory element binding protein (*SREBP*) genes are master regulators of sterol and fatty acid (FA) metabolism. miR-33 controls macrophage immunometabolic response and enhances mitochondrial biogenesis, FA oxidation, and cholesterol efflux. Here, we show that miR-33 levels are increased in bronchoalveolar lavage (BAL) cells isolated from patients with IPF compared with healthy controls. We demonstrate that specific genetic ablation of *miR-33* in macrophages protects against bleomycin-induced pulmonary fibrosis. The absence of miR-33 in macrophages improves mitochondrial homeostasis and increases autophagy while decreasing inflammatory response after bleomycin injury. Notably, pharmacological inhibition of miR-33 in macrophages via administration of anti–miR-33 peptide nucleic acids (PNA-33) attenuates fibrosis in different in vivo and ex vivo mice and human models of pulmonary fibrosis. These studies elucidate a major role of miR-33 in macrophages in the regulation of pulmonary fibrosis and uncover a potentially novel therapeutic approach to treat this disease.

## Graphical abstract

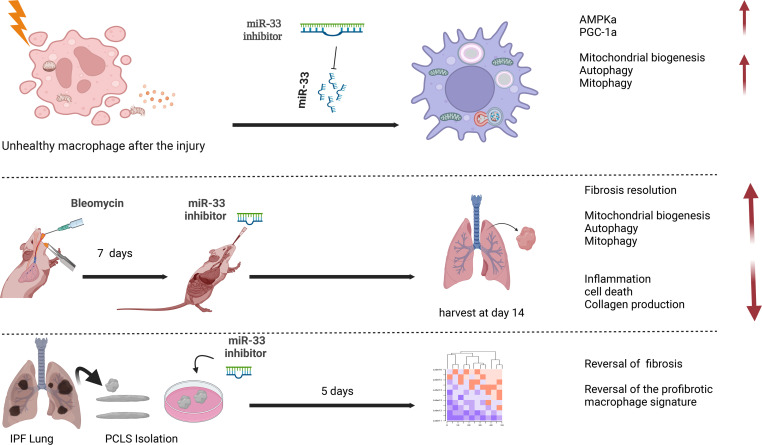

## Introduction

Pulmonary Fibrosis (PF) is a chronic, fatal, and progressive disease characterized by an aberrant wound healing caused by repetitive alveolar epithelial cell injury and excessive deposition of extracellular matrix proteins in the interstitial space of the lung (1). There are 2 main types of pulmonary fibrosis: (a) with unknown etiology (idiopathic) and (b) secondary to autoimmunity exposure to environmental factors such as foreign antigens, toxins, chemical warfare, radiation, or viral infection. The median survival of idiopathic PF (IPF) is 3–5 years after initial diagnosis, and its incidence continues to rise (1–3). Two FDA-approved drugs, pirfenidone and nintedanib, slow disease progression but have no effect on patient symptoms or quality of life, and the only curative treatment for PF is lung transplantation (4).

A growing body of evidence supports the role of macrophages in the pathogenesis of PF. Although many cell types are involved in tissue repair, macrophages have been shown to exhibit critical regulatory activity at all stages of repair and fibrosis (5). Consequently, because macrophages represent potentially important therapeutic targets, there has been a great deal of interest over the past few years in deciphering the contributions of the different macrophage populations that control the initiation, maintenance, and resolution of wound-healing responses (5). Lung macrophages are critical components of the pulmonary fibrotic response in mice and humans, as has been most recently demonstrated by studies that applied single-cell RNA-Seq technologies (6, 7) and by numerous mechanistic studies (8–10). In the past decade, there has been increased recognition of the roles of aging, innate immunity, metabolic reprogramming, and mitochondrial dysfunction in PF (10–13), and it has been shown that mitochondria in alveolar macrophages (AM) from patients with IPF have prominent morphological and transcriptional defects (14). Taken together, manipulation of these macrophages and their metabolic programs may present an attractive therapeutic strategy to treat PF (15).

The miR-33 family (*miR-33a* and *miR-33b*) of intronic miRNAs has been shown to control lipid metabolism as well as cellular energy status and mitochondrial function (16–23). Inhibition of endogenous miR-33 in macrophages promotes mitochondrial biogenesis and efficient production of adenosine triphosphate (ATP) (16, 24). miR-33 sustains cardiac fibrosis and remodeling by preserving lipid raft cholesterol content and controlling proliferation in cardiac fibroblasts (25). Loss or inhibition of miR-33 is protective in multiple models of kidney dysfunction, and this may be, in part, due to a reduction of the repression of fatty acid oxidation (FAO) in fibrotic kidneys and reduced lipid accumulation (26). Additionally, hepatic miR-33 deficiency has been demonstrated to protect against liver fibrosis and improve metabolic function in mice fed a high-fat diet (27). However, the regulatory role of miR-33 in the context of lung fibrosis has not been elucidated yet.

In this study, we discovered that miR-33 regulates the immunometabolic responses of macrophages during PF. Most importantly, we demonstrate that miR-33 therapeutic silencing in lung macrophages attenuates the progression of lung fibrosis in in vivo and ex vivo models. These findings may overcome one of the main problems in treating lung-related disorders with RNA-based medicines: the lack of targeted miRNA delivery technology to minimize off-target effects and improve the safety of miRNAs in vivo (28).

## Results

### miR-33 levels are increased in monocytes/macrophages isolated from bronchoalveolar lavages (BAL) and lungs of patients with IPF compared with healthy controls.

**Figure 1:**
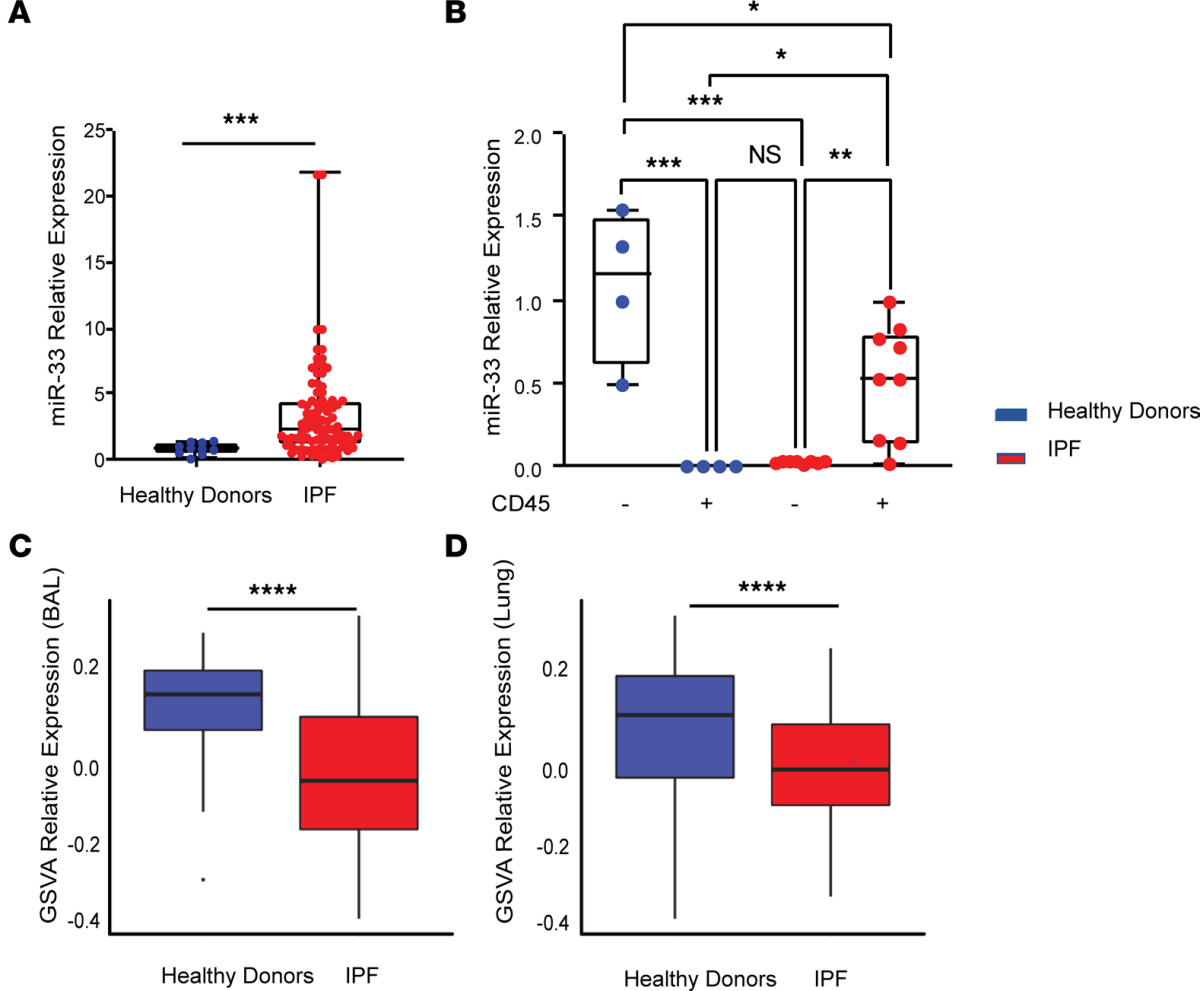
miR-33 levels increase in BAL and lung CD45^+^ cells in patients with IPF, and its target gene expressions decrease in IPF BAL and lung. (**A**) miR-33 relative expression in cells isolated from BAL of patients with IPF (*n* = 62) compared with healthy controls (*n* = 10) by qPCR analysis. (**B**) miR-33 relative expression in hematopoietic cells (CD45^+^) isolated from IPF (*n* = 9) compared with healthy controls (*n* = 4). (**C**) GSVA of miR-33-5p targets in BAL data set (GSE70866), 212 patients with IPF, and 20 healthy donors. (**D**) GSVA of miR-33-5p targets in the Lung LTRC data set (GSE47460), 254 patients with IPF, and 108 healthy donors. All PCR data were analyzed by nonparametric tests (Mann–Whitney *U* test or Kruskal-Wallis test where appropriate) and are presented as mean ± SEM. *P ≤ 0.05, **P < 0.01, ***P < 0.001, ****P < 0.0001.

We evaluated the level of miR-33 in BAL cells, obtained from 62 well-characterized patients with IPF and 10 age-matched healthy controls (Supplemental Table 1; supplemental material available online with this article; https://doi.org/10.1172/jci.insight.158100DS1). This analysis revealed that miR-33 is significantly increased in IPF cells compared with controls (fold change, +2.5; *P* = 0.0024) (Figure 1A). Since more than 90% of BAL cells are immune cells, we aimed to evaluate miR-33 levels in the immune cells of the human lungs. We isolated CD45^+^ cells from the lungs of the patients with IPF (*n* = 9) (Supplemental Table 2) and identified a significant increase in miR-33 levels (*P* = 0.023) in IPF CD45^+^ cells compared with controls (*n* = 4) (Figure 1B). Interestingly, the expression of miR-33 in IPF CD45^–^ populations was significantly lower than controls (*P* ≤ 0.001), and this suggested a potentially important regulatory role for miR-33 in immune cells and perhaps monocyte/macrophage populations in IPF. To expand our analysis and provide a better understanding of miR-33 regulation in humans, we assessed indicators of miR-33 activity in publicly available gene expression lung data sets. We identified miR-33 target genes in 2 gene expression data sets, 1 from lung tissue (Gene Expression Omnibus [GEO] accession no. GSE47460) and 1 from BAL (GSE70866). Since a single gene’s expression is a poor correlate for miRNA activity, we determined overall miRNA activity by analyzing all target genes using Gene Set Variation Analysis (GSVA). This analysis revealed that miR–33-5p target gene expression is significantly decreased in both IPF BAL (5p, *P* = 0.000187; 3p, *P* = 2.57 ***×*** 10^–5^) **(**Figure 1C) and IPF lung tissues (5p, *P* = 2.64 ***×*** 10^–6^) (Figure 1D). Targets of the 3p strand of the microRNA were significantly decreased in BAL but not in lung tissue (Supplemental Figure 1, A and B).

### Myeloid-specific miR-33–KO mice are protected against bleomycin-induced PF.

**Figure 2:**
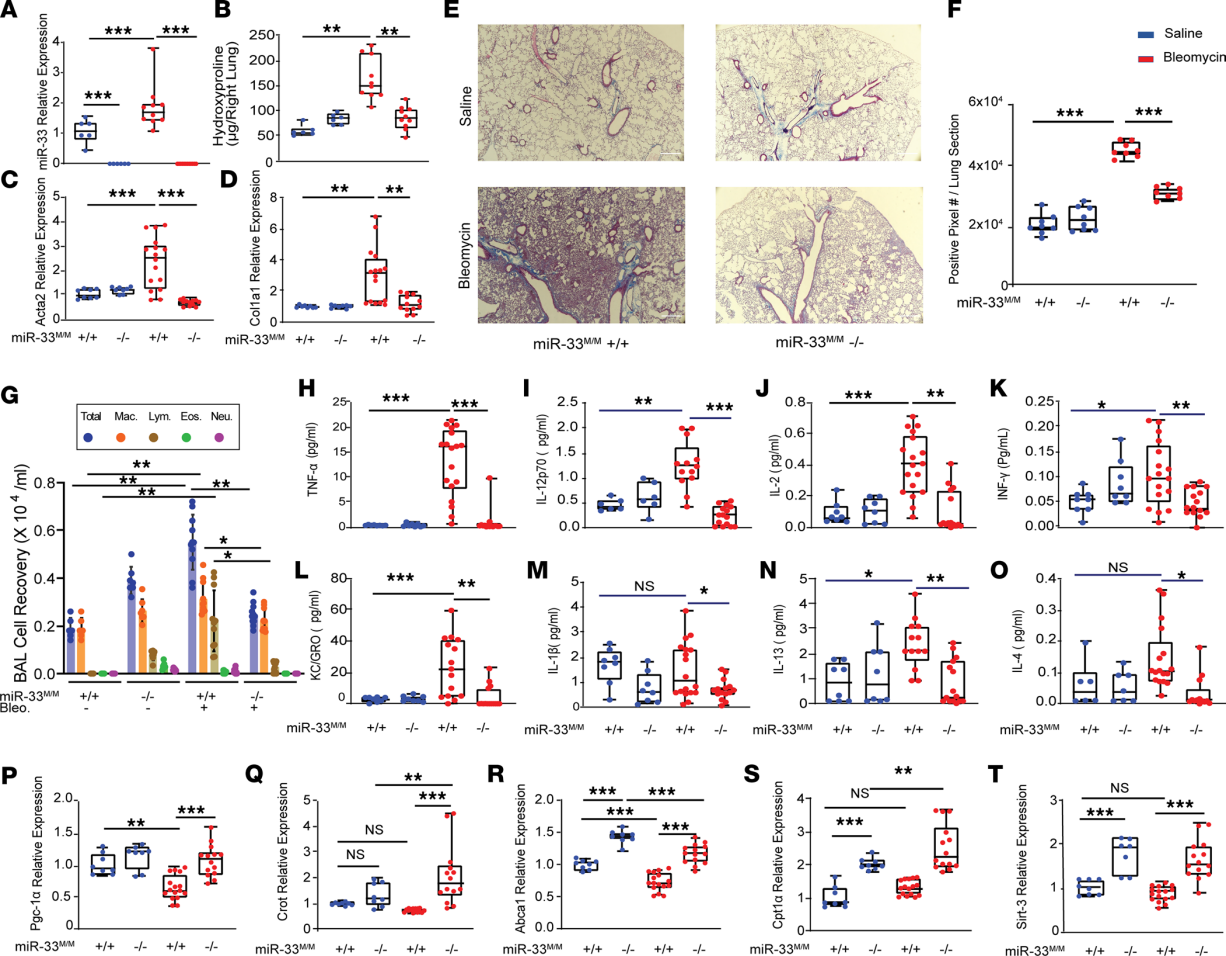
Loss of miR-33 in myeloid cells is protective against bleomycin-induced lung fibrosis and increases mitochondrial-related target gene expression. Evaluation of bleomycin-induced lung fibrosis in myeloid-specific miR-33–KO mice (*miR33^M/M^–/–*) versus controls (*miR33^M/M^+/+*) in bleomycin (red) compared with saline (blue) *n* = 8 for saline and *n* = 16 for bleomycin groups. (**A**) miR-33 relative expression by qPCR analysis in AM isolated from *miR33^M/M^–/–* versus controls *miR33^M/M^+/+*. (**B**) Quantitative analysis of hydroxyproline in lung homogenates from indicated groups of mice. (**C** and **D**) *Acta2* and *Col1a1* relative gene expression by qPCR analysis in mice lungs from indicated groups. (**E** and **F**) Representative images and quantitative measurements of Masson’s trichrome staining of lung sections in *miR33^M/M^–/–* versus controls *miR33^M/M^+/+* with saline and bleomycin. (**G**) Differential cell counts in BAL were harvested from indicated groups. (**H**–**O**) BAL cytokines inflammatory panel in indicated groups: (**H**) TNF-α, (**I**) IL-12p70, (**J**) IL-2, (**K**) INF-γ, (**L**) KC-GRO, (**M**) IL-1β, (**N**) IL-13, and (**O**) IL-4. (**P**–**T**) qPCR analysis of mitochondrial-related miR-33 target genes: (**P**) *Pgc-1α,* (**Q**) *Crot,* (**R**) *Abca1,* (**S**) *Cpt1α*, and (**T**) *Sirt3* in *miR33^M/M^–/–* versus controls *miR33^M/M^+/+* with saline and bleomycin. All data were analyzed by ANOVA or Kruskal-Wallis tests, followed by post hoc analysis, and are presented as mean ± SEM. *P ≤ 0.05, **P < 0.01, ***P < 0.001. Total original magnification, 4***×***.

To directly test the immune cell–specific role of miR-33, we generated myeloid-specific miR-33–KO mice (*LysM^CRE+^miR-33^LOX/LOX^* [*miR-33^M/M^–/–*] and appropriate controls *LysM^CRE–^miR-33^LOX/LOX^* [*miR-33^M/M^+/+*]) and subjected them to a bleomycin-induced PF model. The evaluation of miR-33 levels in BAL AM, as well as the total lungs of these mice, confirmed the absence of miR-33 in macrophages and confirmed that bleomycin administration increased the levels of miR-33 in control mice compared with the saline-treated group (Figure 2A and Supplemental Figure 2A). *miR-33^M/M^–/–* mice were protected from bleomycin-induced lung fibrosis, as indicated by a reduction in collagen content, measured by hydroxyproline assay (fold change, –2; *P* ≤ 0.0001) (Figure 2B) and expression of profibrotic genes including *Col1a1* and *Acta2* (fold change, –2.5; *P* ≤ 0.0001) (Figure 2, C and D). The histological evaluations using Masson’s trichrome staining of lung tissues confirmed these findings (Figure 2, E and F). Next, we evaluated the inflammatory response in these mice via the measurements of BAL cell recovery and the cytokine levels after the bleomycin injury. BAL total cell numbers, as well as macrophage and lymphocyte cell numbers, were significantly increased in the control mice with bleomycin treatment and largely ameliorated in the absence of miR-33 (Figure 2G). Measurements of cytokine panels in the BAL isolated from these mice revealed that many of the inflammatory markers, including TNF-α, IL-12p70, IL-2, INF-γ, KC-GRO, IL-10, IL-13, and IL-4, are decreased in the absence of miR-33 after bleomycin injury (Figure 2, H–O).

We investigated the expression of miR-33 target transcripts relevant to mitochondrial homeostasis in these samples and identified numerous targets, including peroxisome proliferator–activated receptor γ coactivator-1α (*Pgc-1α*, *Ppargc1a*), carnitine o-octanoyltransferase *(Crot*), carnitine palmitoyltransferase-1 α (*Cpt1α*), and ATP binding cassette subfamily A member1 (*Abca1*), with increased expression in the absence of miR-33 at baseline and after bleomycin exposure (Figure 2, P–S). The same pattern of changes was observed in the level of Sirtuin3 (*Sirt3*) expression (Figure 2T), which could be secondary to other effects induced by the absence of miR-33. As expected, the administration of bleomycin reduced the expression of some of these genes in the control mice (Figure 2, P–T). The expression of the miR-33 host gene, Sterol regulatory element binding transcription factor 2 (*Srebp*-*2*), was increased in the lungs of the *miR-33^M/M^–/–* mice (Supplemental Figure 2, B and C). These data reveal the protective effect of macrophage-specific miR-33 ablation in the bleomycin model of PF in mice and suggested that this effect could be via alterations in the expression of target genes relevant to mitochondrial function.

### Pharmacological inhibition of miR-33 in lung macrophages protects against bleomycin in an in vivo murine model of PF.

**Figure 3:**
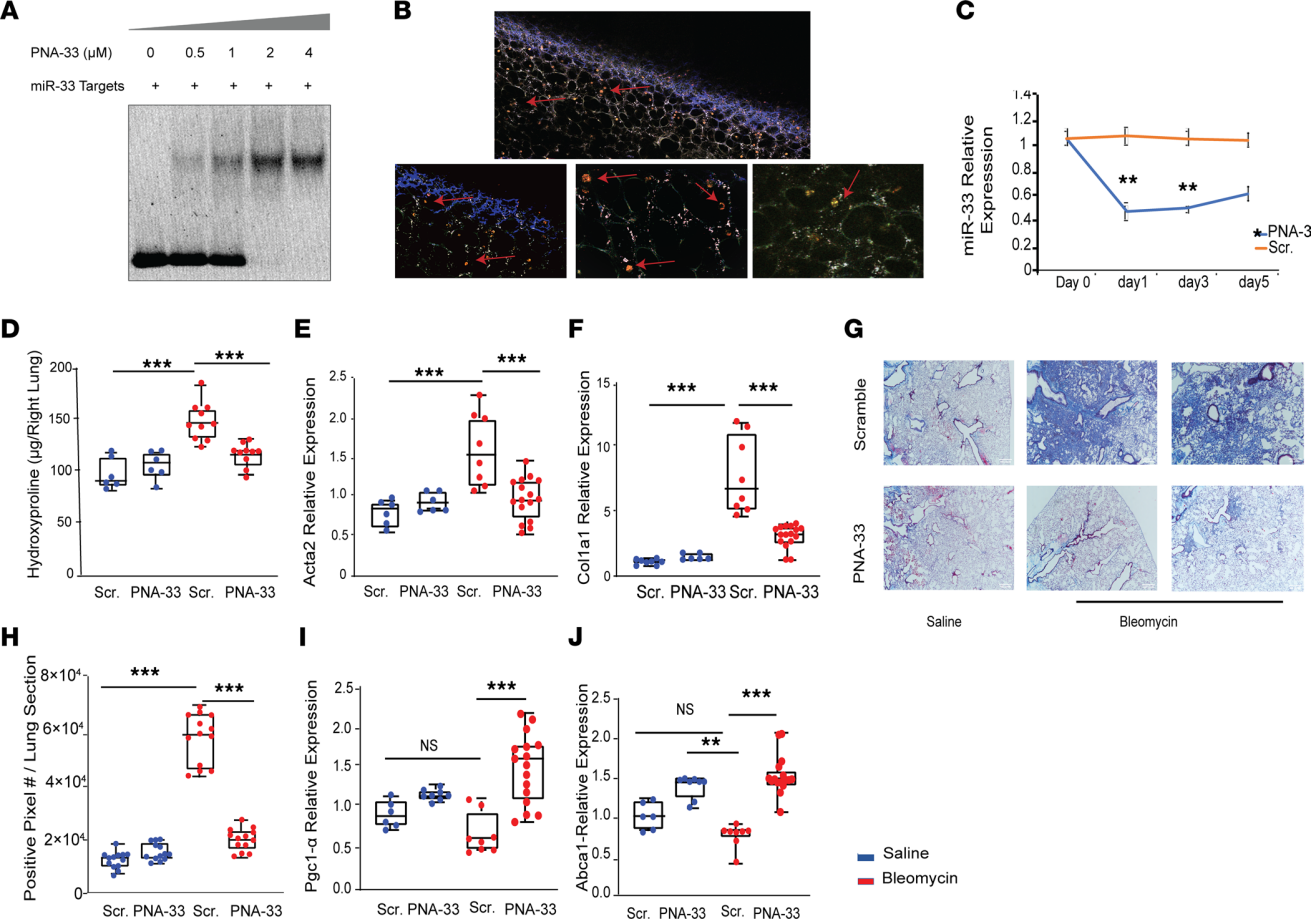
Pharmacological inhibition of miR-33 using PNA-33 in lung macrophages protects against bleomycin-induced pulmonary fibrosis in in vivo mouse model of PF. (**A**) Binding efficiency of PNA-33 to the known miR-33 targets by gel shift assay on 10% nondenaturing polyacrylamide gel. Bound and the unbound fraction of target miR-33 were visualized by staining the gel in SYBR Gold. (**B**) Two-photon microscopy imaging of PNA-33 TAMRA conjugated in WT mice 24 hours after i.v. administration (red arrows indicate orange accumulation of TAMARA dye in macrophages). Total original magnification, (top panel) 4***×***, (Lower panels) 20***×***. (**C**) miR-33 relative expression in AM of WT mice after i.n. administration of PNA-33 and scrambled control in different time points (days 0, 1, 3, and 5). (**D**) Quantitative analysis of hydroxyproline in lung homogenates from indicated groups of mice in bleomycin-induced lung fibrosis model. (**E** and **F**) *Acta2* and *Col1a1* relative gene expression by qPCR analysis in mice lungs from indicated groups. (**G** and **H**) Representative images and quantitative measurements of Masson’s trichrome staining of lung sections after administration of PNA-33/scrambled control in saline and bleomycin. Total original magnification, 4***×***.(**I** and **J**) qPCR analysis of mitochondrial related miR-33 target genes: *Pgc-1α* and *Abca1* after PNA-33/scrambled control in saline and bleomycin groups. Bleomycin; red, Saline; blue. *n* = 6 for saline, *n* = 8 for bleomycin groups. All data were analyzed by ANOVA or Kruskal-Wallis tests, followed by post hoc analysis, and are presented as mean ± SEM. *P ≤ 0.05, **P < 0.01, ***P < 0.001.

Peptide nucleic acids (PNAs) are synthetic analogs of DNA composed of nucleobases that are connected to a pseudo-peptide backbone, which imparts resistance to nuclease enzymes and considerably increases stability and half-life (29, 30). We generated and assessed the efficacy of anti–miR-33 PNA (PNA-33) as a cell-specific inhibitor for miR-33. Our results show that PNA-33 binds specifically to well-established miR-33 target transcripts by gel shift assay (Figure 3A). In prior studies, we optimized the protocol for the preparation of PNA conjugates containing the fluorophore TAMRA (31). We administered PNA-33 TAMRA–conjugated anti-miR in WT mice via an i.v. route and confirmed the accumulation of the compound in lung macrophages 24 hours after injection using a 2-photon microscopy imaging system (Figure 3B). Next, we administered PNA-33 and scrambled control in WT mice via an i.n. route, which is the most efficient method for direct delivery to the lung and isolated lungs and AM at different time points. As shown in Figure 3C, miR-33 levels were dramatically decreased, specifically in AM after PNA-33 administration, and they remained at low levels even 5 days after a single administration of PNA-33 (Figure 3C). After confirming the efficacy of PNA-33 in delivering and antagonizing miR-33 in macrophages, we tested whether therapeutic silencing of miR-33 using PNA-33 protects against bleomycin-induced PF in mice. Notably, we found that intranasal administration of PNA-33 markedly reduces lung fibrosis, as indicated by a significant decrease in collagen content and fibrogenic gene expression (*Col1a1* and *Acta2*) compared with mice treated with scrambled control (Figure 3, D–F). Histological assessments in lung sections via quantifications of the collagen accumulations in Masson’s trichrome staining confirmed the fibroprotective effects of PNA-33 in this mice model (Figure 3, G and H). Consistent with what we observed in *miR-33^M/M^–/–* mice, the expression of mitochondrial-related miR-33 target genes *Pgc-1α* and *Abca1* was increased in response to PNA-33 treatment (Figure 3, I and J).

Our results demonstrate that macrophage-specific deletion and pharmacological inhibition of miR-33 in the lung protect against bleomycin-induced PF in in vivo model.

### The absence of miR-33 in macrophages improves mitochondrial homeostasis at baseline and after bleomycin injury.

**Figure 4:**
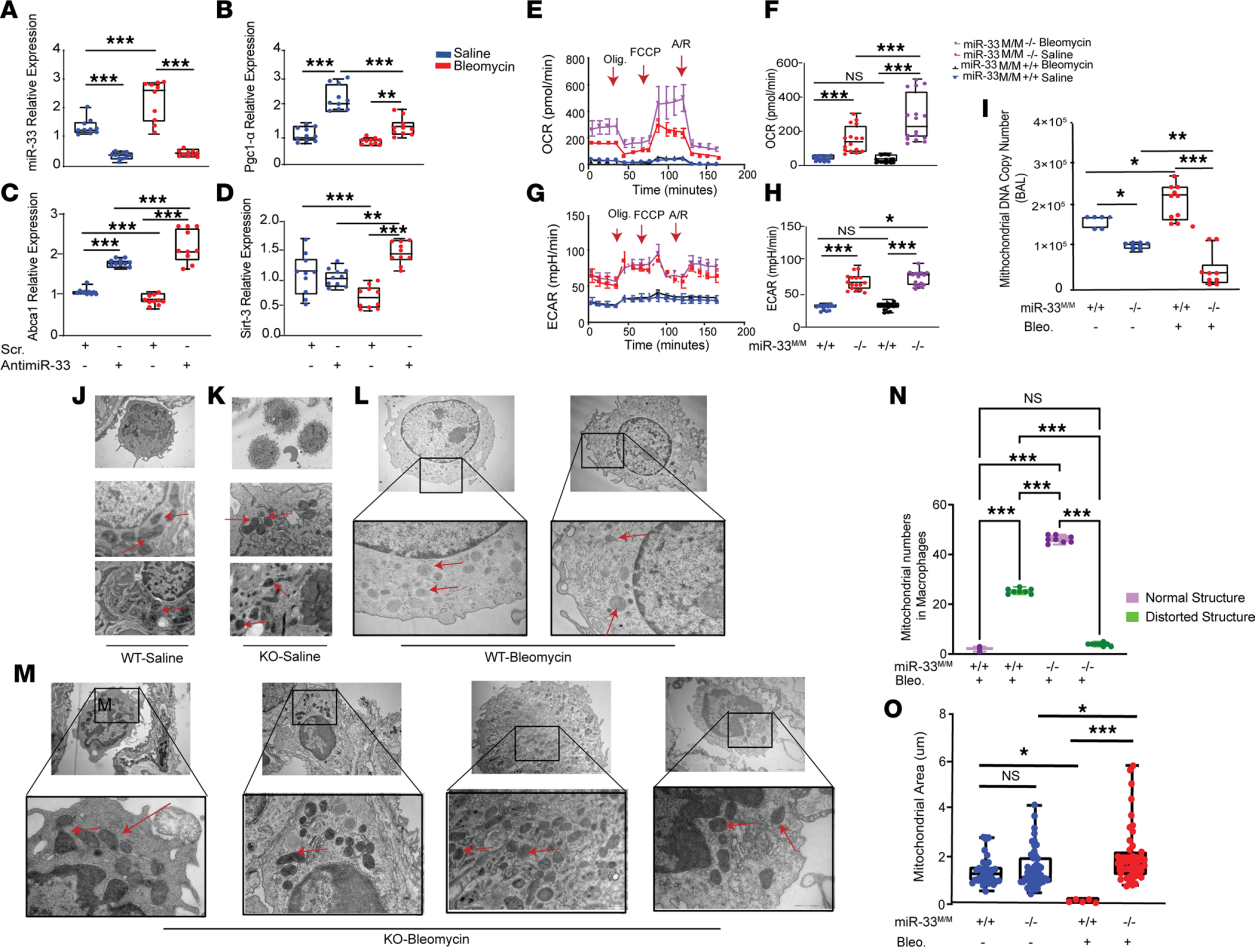
The absence of miR-33 in macrophages improves mitochondrial homeostasis (function and structure) at baseline and after bleomycin injury. (**A**–**D**) qPCR analysis of miR-33 and target gene expression in response to inhibition by miR-33 antagomir in AM in vitro with and without bleomycin (*n* = 10). miR-33, *Pgc-1α, Abca1*, and *Sirt3* relative expressions. (**E**–**H**) Seahorse analysis of AM isolated from *miR33^M/M^–/–* versus miR*33^M/M^+/+* in response to bleomycin and saline. The analysis was measured under basal conditions followed by the addition of oligomycin, FCCP, rotenone, and antimycin (*n* = 16 in all groups). (**E** and **F**) Oxygen consumption rate (OCR, pmol/min). (**G** and **H**) Extracellular acidification rate (ECAR, pmpH/min). (**I**) Measurement of free circulating mtDNA by qPCR in the BAL isolated from *miR33^M/M^–/–* versus *miR33^M/M^+/+* mice in response to bleomycin and saline (*n* = 6 for saline, *n* = 10 for bleomycin groups). (**J**–**M**) Representative images of transmission electron microscopy (TEM) imaging on lung tissues isolated from *miR33^M/M^–/–* versus *miR33^M/M^+/+* mice in response to bleomycin and saline (*n* = 8). Red arrows indicate mitochondria. (**N**) Blinded measurements of mitochondria in TEM images from mice AM in *miR33^M/M^–/–* versus *miR33^M/M^+/+* mice in bleomycin-treated mice by counting the dysmorphic versus normal-looking mitochondria in different groups (*n* = 8). (**O**) Ultrastructural qualitative and quantitative analysis of mitochondria in mice lung TEM images represented as mitochondrial area (au) in *miR33^M/M^–/–* versus *miR33^M/M^+/+* mice in bleomycin- and saline-treated mice. The statistical test used were ANOVA or Kruskal-Wallis tests, followed by post hoc analysis. All data are presented as mean ± SEM. *P ≤ 0.05, **P <0.01, ***P < 0.001. Total original magnification, 4***×*** (**J** and **K**, top panels in **L** and **M**) and 10***×*** (lower panels in **L** and **M**).

To understand the mechanism behind the fibroprotective effects of miR-33 inhibition and to confirm its regulatory role in the expression of genes associated with mitochondrial biogenesis and metabolism in macrophages, we tested primary mice AM in an ex vivo system. Inhibition of miR-33 using a miR-33 antagomir with or without bleomycin treatment revealed a significant increase in the expression of miR-33 target genes *Pgc-1α*, *Abca1*, and *Sirt3*, indicating a role of miR-33 in regulating key mitochondrial pathways (Figure 4, A–D). To further explore the regulatory role of miR-33 in mitochondrial homeostasis in macrophages, we measured mitochondrial function in primary AM isolated from myeloid-specific miR-33–KO mice by Seahorse XF96 Extracellular Flux Analyzer. As expected, mitochondrial activity in AM of control mice was very limited at baseline and after bleomycin injury. Interestingly, we found a dramatic increase in mitochondrial function, as indicated by a significant increase in oxygen consumption rate (OCR, pmol/min) and extracellular acidification rate (ECAR, pmpH/min) (fold change, +3), in the *miR-33^M/M^–/–* AM compared with control AM at baseline and after bleomycin exposure (Figure 4, E–H).

Emerging evidence suggests that the examination of circulating mtDNA could be a mechanism-based prognostic biomarker of PF (32). Thus, we evaluated mtDNA in the extracellular fraction of the BAL isolated from *miR-33^M/M^–/–* and controls after bleomycin injury (32). Notably, we observed a significant increase in mtDNA content in control mice after bleomycin injury but not in the absence of miR-33 in macrophages after bleomycin or even at baseline (Figure 4I). Since we observed an improved oxygenation rate and decreased extracellular mitochondrial DNA in the BAL, we hypothesized that miR-33 deficiency improves mitochondrial stability. To evaluate the effect of miR-33 inhibition on structural alterations in the mitochondria in the lung, we performed transmission electron microscopy (TEM) imaging analyses on lung tissues as well as AM isolated from control and *miR-33^M/M^–/–* mice (Figure 4, J–M). Ultrastructural qualitative and quantitative analysis revealed that the mitochondria in macrophages from control mice after bleomycin injury were dysmorphic and contained disorganized cristae (Figure 4, L, N, and O). These deleterious morphological mitochondrial alterations were partially prevented in macrophages from *miR-33^M/M^–/–* mice. Detailed morphometric analysis of macrophages from *miR-33^M/M^–/–* mice demonstrated a significant improvement in the mitochondrial structure as well as an increase in the mitochondrial number and area at the baseline and, strikingly, after bleomycin exposure (Figure 4, M–O).

### The absence of miR-33 in macrophages induces autophagy after bleomycin injury.

**Figure 5:**
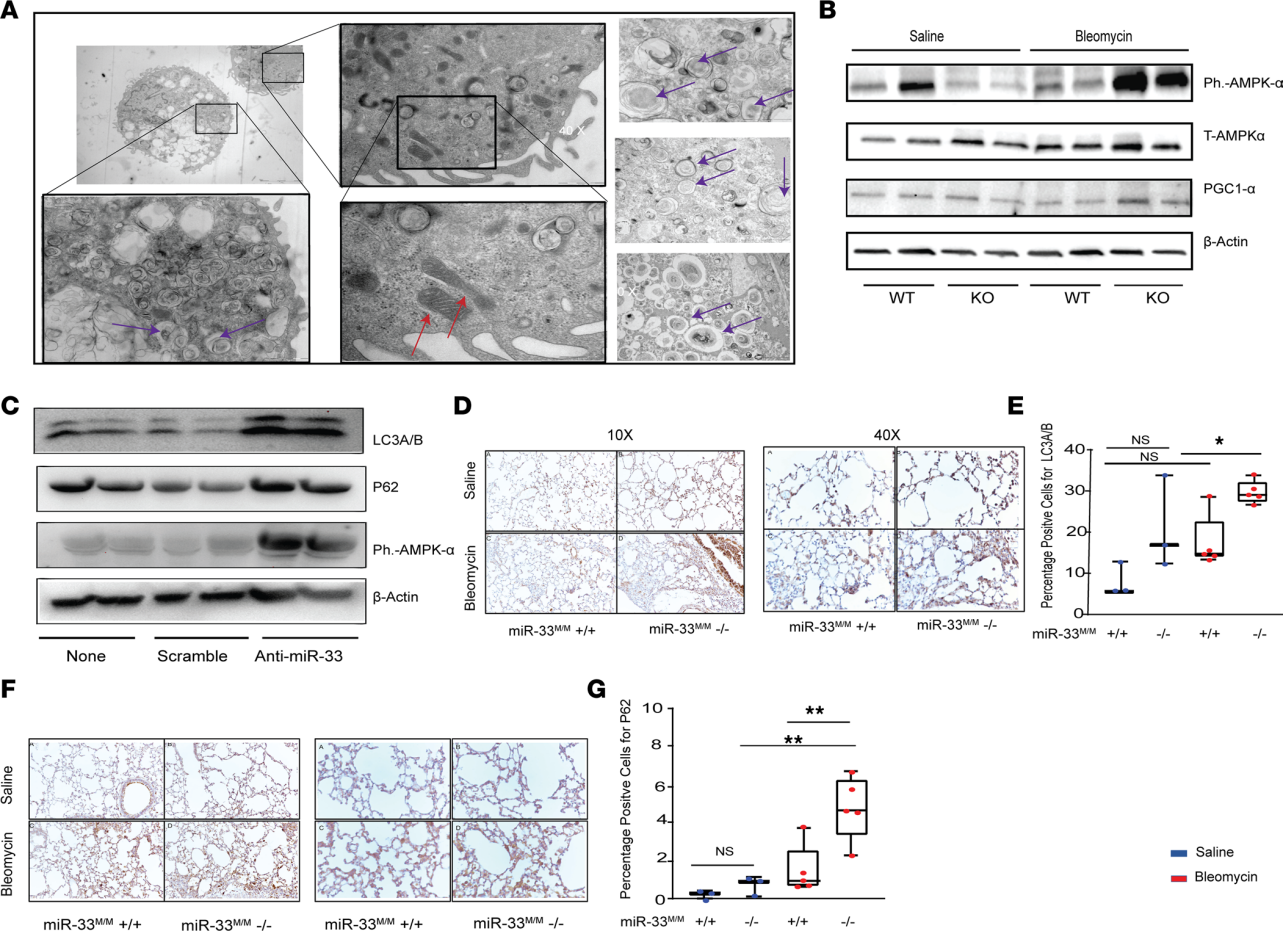
Genetic ablation of miR-33 in lung macrophages induces autophagy after bleomycin injury. (**A**) Representative images of transmission electron microscopy (TEM) imaging on lung tissues isolated from *miR33^M/M^–/–* after bleomycin. There is a dramatic increase in autophagosome contents, only in the *miR33^M/M^–/–* macrophages after bleomycin along with the improvement in mitochondrial structure in these cells (*n* = 6). Red arrows indicate mitochondria, and purple arrows indicate autophagosome. Total original magnification, 4***×***, 10***×***, and 20***×***. (**B**) Western blot analysis of phospho–AMPK-α (Ph.–AMPK-α) and PGC-1α in the lung homogenates isolated from *miR33^M/M^–/–* and controls after bleomycin and saline. (**C**) Western blot analysis of Ph.–AMPK-α, LC3A/B, and P62 in AM after inhibiting miR-33 by miR-33 antagomir. (**D** and **E**) Representative images and quantification of IHC staining of LC3A/B in lung tissues isolated from *miR33^M/M^–/–* and controls after bleomycin and saline treatment. (**F** and **G**) Representative images and quantification of IHC staining of P62 in lung tissues from miR33^M/M^–/– and controls after bleomycin and saline treatment. All data were analyzed by ANOVA or Kruskal-Wallis tests, followed by post hoc analysis, and are presented as mean ± SEM. *P ≤ 0.05, **P < 0.01. Total original magnification, 4***×*** (**D** and **F**).

We extended our investigation by further evaluating the TEM images acquired from the lung tissues and AM from *miR-33^M/M^–/–* mice after bleomycin exposure. In addition to the improvement in mitochondrial structure, we found a dramatic enhancement in autophagy, as visualized by an increase in autophagosome contents, specifically in the *miR-33^M/M^–/–* macrophages after bleomycin (Figure 5A). Given that AMP-activated protein kinase α (*AMPK-α*) and *PGC-1α* are well-established miR-33 target genes, relevant to mitochondrial homeostasis and autophagy, we evaluated their protein expressions in these mice. Western blot analysis of the lung tissues revealed an increase in the expression of phospho–AMPK-α and PGC-1α in *miR-33^M/M^–/–* mice at baseline and after bleomycin exposure compared with control mice (Figure 5B). To confirm the relationship between the absence of miR-33 in macrophages and autophagy, we also evaluated AM after the inhibition of miR-33 using an antagomir and found an increase in phospho–AMPK-α as well as LC3A/B and P62 (also known as SQSTM1) expression in these cells compared with the scrambled control (Figure 5C). IHC staining in lung tissues isolated from *miR-33^M/M^–/–* mice showed a significant increase in LC3A/B^+^ (Figure 5, D and E) and P62^+^ (Figure 5, F and G) cells in these mice compared with controls after bleomycin injury. Co-IHC of LC3A/B and P62, along with F4/80 (a marker of macrophages in the lung), identified colocalization of F4/80 with LC3A/B and P62 in the lungs of *miR-33^M/M^–/–* mice after bleomycin (Supplemental Figure 3, A–C).

### miR-33 deficiency in macrophages induced mitophagy in response to injury.

**Figure 6:**
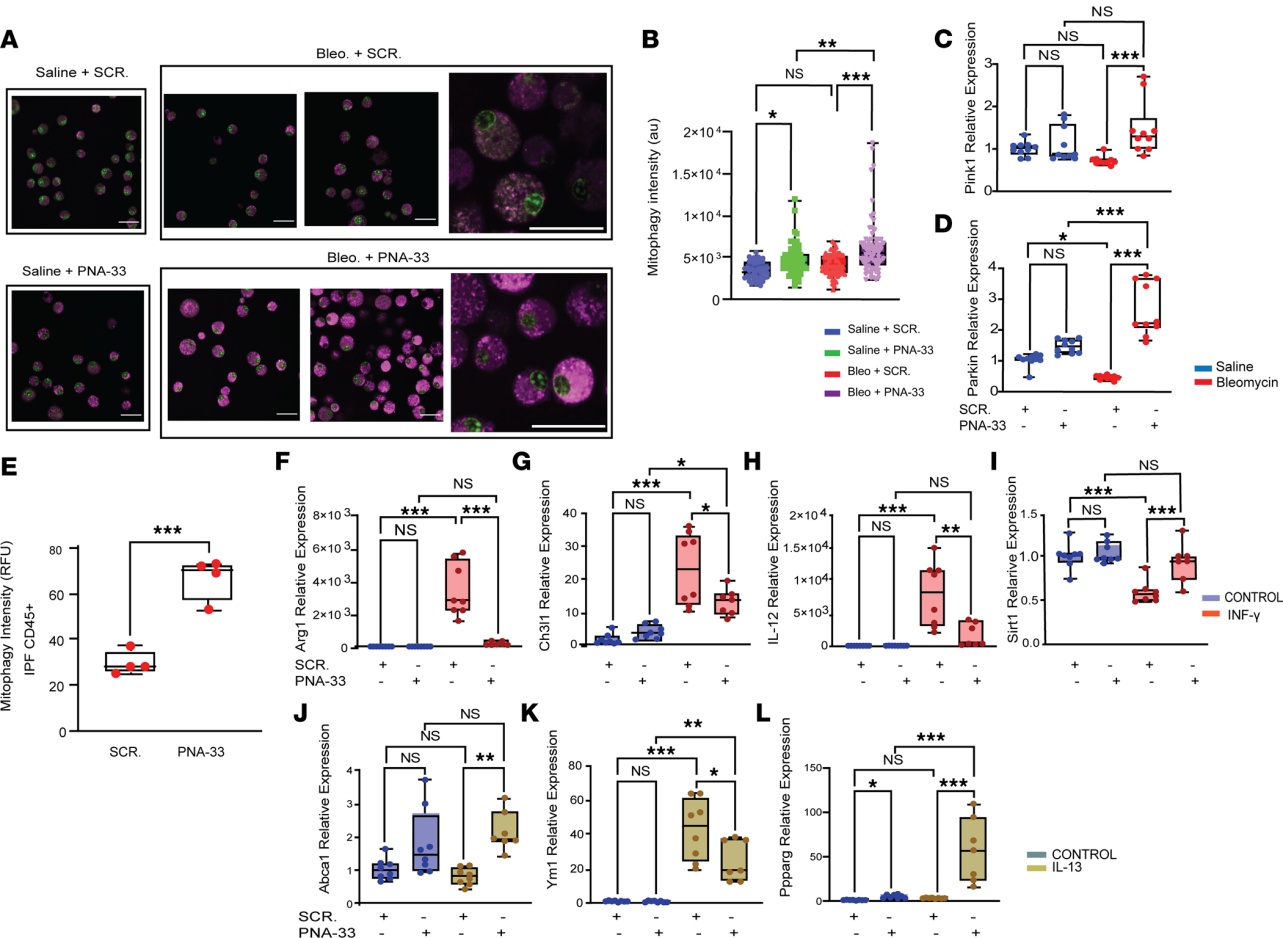
The absence of miR-33 in macrophages induces mitophagy in response to injury and alters cytokine-induced gene expressions in AM. (**A** and **B**) Mitophagy assay in primary AM isolated from WT mice treated with PNA-33 (2 nM) or scrambled control after bleomycin (15 nM) or saline. (**A**) Representative images in indicated groups. (**B**) Quantitation of mitophagy staining. (**C** and **D**) *Pink1* and Parkin expression in PNA-33/scramble-treated primary mice AM after bleomycin/saline. (**E**) Mitophagy measurement (RFU) in CD45^+^ cells isolated from human IPF lungs in response to PNA-33 versus scrambled control after 24 hours in culture (*n* = 4). (**F**–**L**) Evaluation of the effects of PNA-33/scramble on primary mice AM after cytokines stimulation. Primary mice AM were treated with PNA-33 (2 nM) or scrambled control for 24 hours before exposing them to IL-13 or INF-γ + LPS for another 24 hours. (**F**–**I**) Expression of *Arg1*, *Chi3l1*, *IL-12,* and *Sirt1* in INF-γ–treated cells PNA-33/scramble treatments. (**J**–**L**) Expressions of *Ym1*, *Abca1*, and *Pparg* in *IL-13–*treated cells after PNA-33/scramble treatments (*n* = 7). All data were analyzed by ANOVA or Kruskal-Wallis tests, followed by post hoc analysis, and data are presented as mean ± SEM. *P ≤ 0.05, **P < 0.01, ***P < 0.001. Total original magnification, 4***×*** and 10***×***.

Mitophagy is a quality control mechanism that could be regulated independently of the signals that govern autophagy (33); therefore, we sought to study the effects of miR-33 inhibition on mitophagy after bleomycin injury. To this end, we isolated primary AM from WT mice and treated them with PNA-33 or scrambled control after bleomycin injury. Confocal imaging analysis demonstrated that miR-33 inhibition markedly induced mitophagy at baseline and after bleomycin injury in macrophages (Figure 6, A and B). We next evaluated *Pink1* and *Parkin* gene expression and found a significant increase in the expression of these 2 critical regulators of mitophagy in the miR-33–deficient AM after bleomycin treatment (Figure 6, C and D). Notably, mitophagy was also increased in CD45^+^ cells isolated from patients with IPF after miR-33 inhibition (Figure 6E), further confirming the concordance of this finding between human and mice lung fibrosis.

Taken together, these data demonstrate the fibroprotective role of macrophage miR-33 ablation via improvement of mitochondrial function and structure at baseline and after bleomycin injury as well as via an increase in autophagy/mitophagy in these cells.

### Targeted inhibition of miR-33 in macrophages alters the proinflammatory/antiinflammatory gene expression profile.

Given the importance of macrophages in the Th1/Th2 paradigm in PF, we assessed how miR-33 inhibition influenced macrophage immune responses. We isolated AM from WT mice and skewed them toward M1 (using INF-γ + LPS) or M2 (using IL-13) after miR-33 inhibition using PNA-33. As was expected, INF-γ induced the expression of proinflammatory genes, including arginase 1 (*Arg1*), chitinase 3 like 1 (*Chi3L1*), and *IL-12*; interestingly, the expression of all these profibrotic markers decreased after miR-33 inhibition (Figure 6, F–H). Conversely, INF-γ decreased the expression of *Sirt-1*, which was reversed after PNA-33 treatment (Figure 6I).

M2 alternative activated macrophages gene expression analysis revealed that miR-33 ablation reversed the IL-13 induced *YM1* (also known as Chitinase like 3 [*Chil3*]) levels in these cells (Figure 6K). Notably, miR-33 inhibition of IL-13–treated cells increased the expression of *Abca1* and *Ppar-γ* (Figure 6, J and L).

This experiment suggests a potential regulatory role for miR-33 in skewing proinflammatory/antiinflammatory macrophages that may be beneficial in fibrosis resolution.

### Lack of miR-33 in myeloid cells improves mitochondrial homeostasis and decreases cell death in alveolar type II cells after bleomycin injury.

**Figure 7:**
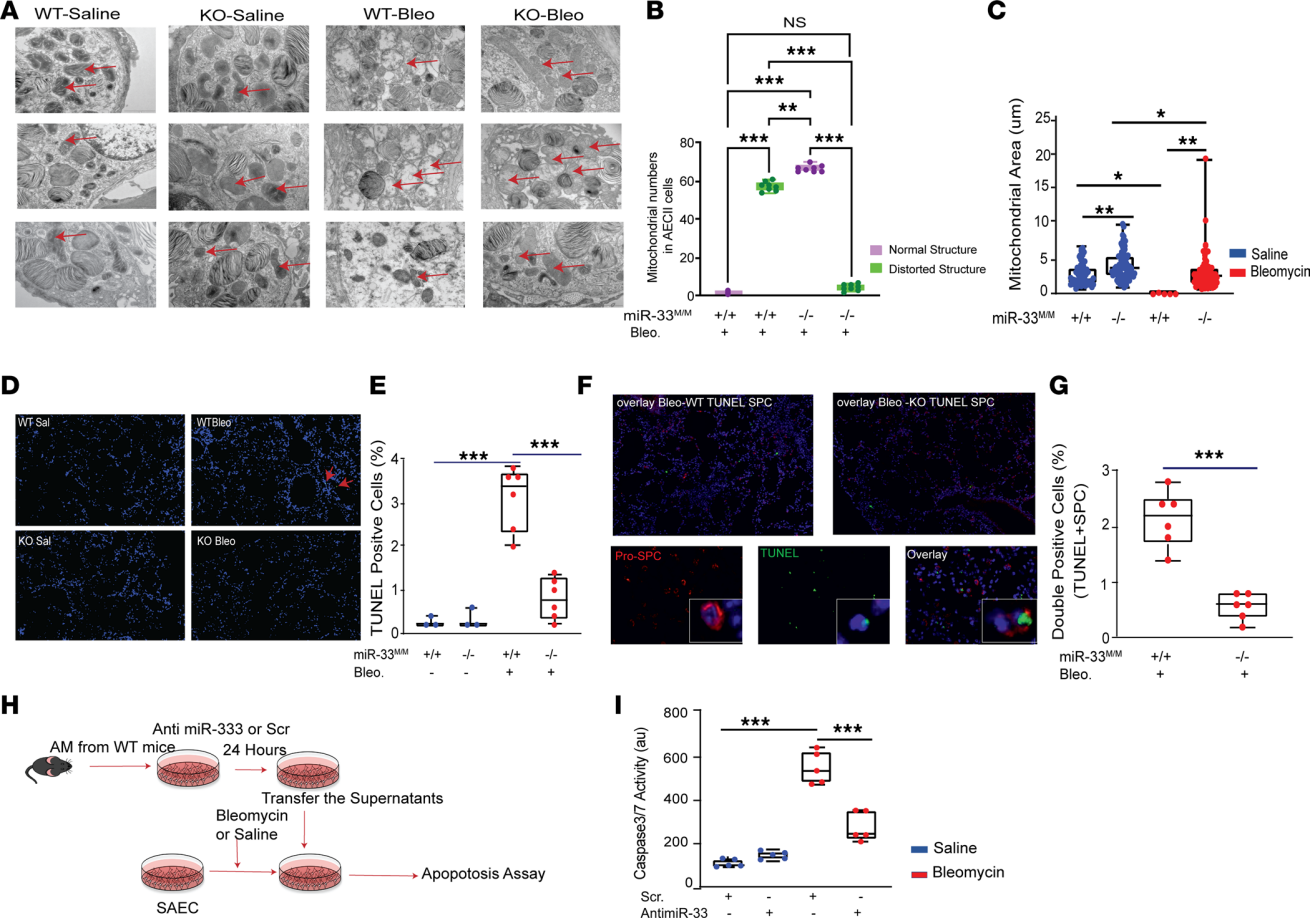
The absence of miR-33 in lung macrophages improves mitochondrial homeostasis and decreases cell death in AT2 after bleomycin injury. (**A**) Representative images of transmission electron microscopy (TEM) on lung tissues isolated from *miR33^M/M^–/–* and *miR33^M/M^+/+* after bleomycin and saline treatment. Red arrows indicate mitochondria in AT2 (*n* = 8 per group). Total original magnification, 4***×*** and 10***×***. (**B**) Blinded measurements of mitochondria in mice AT2 after bleomycin in TEM images by counting the dysmorphic versus normal-looking mitochondria in *miR33^M/M^–/–* and control groups (*n* = 8). (**C**) Ultrastructural qualitative and quantitative analysis of mitochondria in mice lung AT2s in TEM images represented as mitochondrial area (au). (**D** and **E**) Representative images and quantification analysis of TUNEL IF staining on lung sections from *miR33^M/M^–/–* and *miR33^M/M^+/+* after bleomycin and saline treatment (*n* = 6). Green, TUNEL^+^ cells. Total original magnification, 4***×***. (**F**) Representative images of TUNEL IF staining with Pro-SPC on lung sections from *miR33^M/M^–/–* and *miR33^M/M^+/+* after bleomycin treatment (*n* = 6). Green, TUNEL; red, SPC. Total original magnification, 4***×*** and 20***×.*** (**G**) Quantification analysis of TUNEL^+^SPC^+^ cells on lung sections from *miR33^M/M^–/–* and *miR33^M/M^+/+* after bleomycin (*n* = 6). (**H** and **I**) Schematic experimental planning and quantification of Caspase 3/7 activity measured in small airway epithelial cells (SAEC) with and without bleomycin after exposure to the supernatants harvested from ablated miR-33 AM (using PNA-33 or scrambled control) from WT mice. All data were analyzed by ANOVA or Kruskal-Wallis tests, followed by post hoc analysis, and are presented as mean ± SEM. *P ≤ 0.05, **P <0.01, ***P < 0.001.

Given the importance of cell-to-cell communications between macrophages and other players — such as alveolar epithelial cells — in the pathogenesis of PF (34), we sought to determine the impact of myeloid-specific miR-33 deficiency on alveolar epithelial cells. Improvement of mitochondrial integrity and function in alveolar type II cells (AT2) has been shown to have antifibrotic effects (11). We analyzed AT2 morphology in the lung tissue of *miR-33^M/M^–/–* mice after bleomycin exposure. As previously reported (35), we confirmed that most of the mitochondria in AT2s of the control mice after bleomycin cells were structurally abnormal and dysmorphic (Figure 7, A and B). The morphometric quantification of the mitochondria in AT2s in the *miR-33^M/M^–/–* mice revealed a significant improvement in mitochondrial structure and morphology by area and number at baseline and after bleomycin injury (Figure 7, A–C).

After observing that the absence of miR-33 in the macrophages preserves bleomycin-induced mitochondrial abnormalities in AT2, we further aimed to determine the effects of miR-33 ablation on mitochondria-regulated apoptotic pathways. To this end, we performed a TUNEL assay on the lung tissues after bleomycin and identified a significant increase in apoptosis as indicated by TUNEL^+^ cells in control mice after bleomycin (Figure 7, D and E). We observed a significant reduction in apoptosis in the lungs of *miR-33^M/M^–/–* mice after bleomycin (Figure 7, D and E). We also analyzed whether AT2s in these mice are protected against apoptosis by combining the TUNEL assay with pro–surfactant protein C (Pro-SPC) IF. We found a significant reduction in apoptosis of AT2s (double-positive cells) in *miR-33^M/M^–/–* mice compared with the control mice after bleomycin exposure (Figure 7, F and G).

We extended our investigation by performing a deeper analysis of this macrophage–epithelial cell interaction in vitro. To this end, we inhibited miR-33 in AM isolated from untreated WT mice and transferred supernatants from these cells, after miR-33 ablation, to cultured small airway epithelial cells (SAEC). We found that these supernatants significantly reduced bleomycin-induced apoptosis measured by Caspase 3/7 activity in SAEC compared with the supernatants from AM treated with scrambled control (Figure 7, H and I), concordant with the in vivo reduction of the inflammatory cytokines in the BAL of the *miR-33^M/M^–/–* mice after bleomycin (Figure 2, H–O). This observation provides evidence for beneficial crosstalk from miR-33–depleted macrophages to a key lung structural cell such as AT2.

Our results reveal that the absence of miR-33 in macrophages could have a protective role on AT2s by improving mitochondrial homeostasis and reducing apoptosis in these cells after bleomycin.

### Pharmacological inhibition of miR-33 in lung macrophages protects against ex vivo murine models of PF.

**Figure 8:**
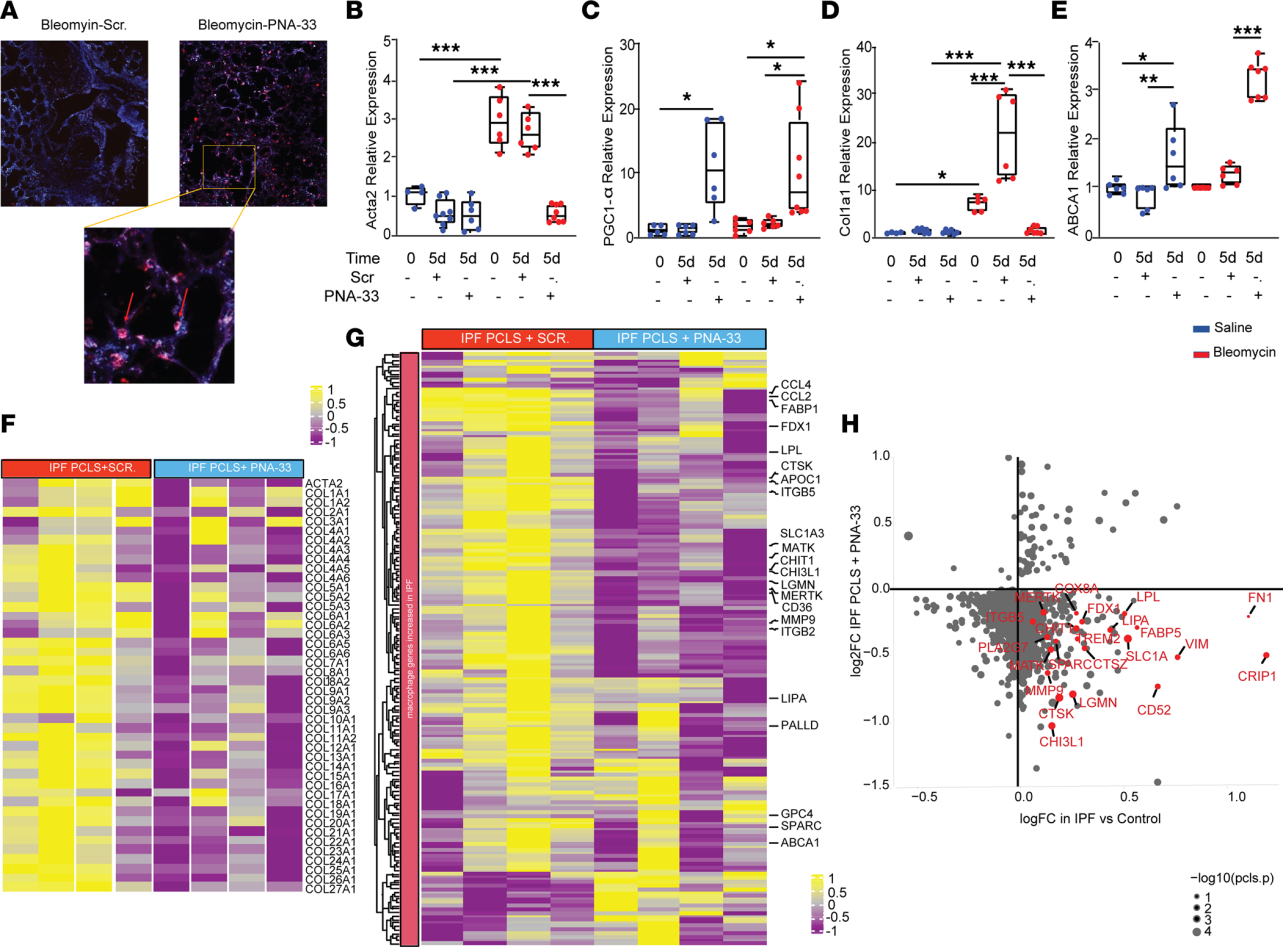
Pharmacologic inhibition of miR-33 using PNA-33 ameliorates fibrosis in mice and human ex vivo models of PF. (**A**–**E**) Evaluation of antifibrotic effects of PNA-33 in murine ex vivo model. Mouse PCLS isolated at day 14 after bleomycin or saline treatment. Bleomycin, red; saline, blue. (**A**) Two-photon microscopy imaging of mice PCLS from the bleomycin-treated group at the end of 5 days of stimulation with PNA-33 TAMRA conjugated or scrambled control. Blue, collagen; orange, TAMRA accumulation in macrophages. Total original magnification, 4***×*** and 10***×***. (**B**–**D**) qPCR analysis of *Acta2*, *Col1a1, Pgc-1α*, and *Abca1* in mouse PCLS after bleomycin following 5 days of stimulation with PNA-33 or scramble (*n* = 6 per group). All data were analyzed by ANOVA or Kruskal-Wallis tests, followed by post hoc analysis, and are presented as mean ± SEM. *P ≤ 0.05, **P < 0.01, ***P < 0.001. (**F**–**H**) Evaluation of antifibrotic effects of PNA-33 in human ex vivo model. hPCLS prepared from human IPF lungs isolated and treated with PNA-33 or scrambled control for 5 days before performing RNA-Seq. (**F**) Heatmap showing the fibrotic gene expression in IPF PCLS treated with PNA-33 or scramble. (**G**) Heatmap showing the profibrotic macrophage gene expression alterations by PNA-33 in IPF PCLS. (**H**) Scatterplot of genes found significantly differentially expressed in IPF lung macrophage versus controls (in single-cell RNA-Seq analysis) compared with IPF PCLS treated or untreated with miR-33 inhibitor (PNA-33). The *x* axis corresponds to the log fold change differences in IPF versus control lung macrophage reported in single-cell RNA-Seq analysis, the *y* axis corresponds to fold change differences in IPF PCLS following treatment with PNA-33. The size of each dot corresponds to the negative log_–10_ transformed *P* values of a comparison of IPF PCLS with or without miR-33 treatment.

To extend our investigation and to confirm the fibroprotective effects of miR-33 ablation using PNA-33, we used murine precision-cut lung slices (mPCLS) generated from the bleomycin-induced PF model. We isolated lung slices from WT mice on day 14 after bleomycin and treated them with PNA-33 and/or scrambled control for 5 days ex vivo. We also used PNA-33–TAMRA conjugated to validate the successful uptake of this PNA by macrophages in these PCLS. As shown in Figure 8A, PNA-33 not only accumulates in macrophages in PCLS, but it also attenuates collagen accumulation measured by second harmonic generation (SHG) microscopy compared with control slices (Figure 8A). This assay also confirmed that there is a significant decrease in fibrogenic gene expression (*Col1a1* and *Acta2*) as well as an increase in miR-33 target gene expressions (*Pgc-1a* and *Abca1*) in PNA-33–treated lung slices compared with controls (Figure 8, B–E). 

### Pharmacologic inhibition of miR-33 ameliorates fibrosis in human PCLS from patients with IPF.

We sought to assess how miR-33 inhibition using PNA-33 affects PF in human lungs. We utilized an ex vivo culture of human PCLS (hPCLS) prepared from human IPF lungs and treated with PNA-33 or scrambled control for 5 days. At the end of the treatment, we performed bulk RNA-Seq to identify the effects of miR-33 inhibition on gene alterations in these fibrotic lung slices. GSVA did not demonstrate a statistically significant effect on all miR-33 target genes; however, there was a significant increase in the expression of multiple relevant miR-33 target genes, including *PPARGC1A* (*PGC-1α*; *P* = 0.03) and ATP Binding Cassette Subfamily G Member1 (*ABCG1*; *P* = 0.012) after PNA-33 treatment (Supplemental Figure 4, A–D). Further transcriptomic analysis of this IPF PCLS data set revealed that PNA-33 reduced numerous fibrosis-relevant genes including *ACTA2* (*P* = 0.01; fold change, 0.7) and multiple collagen genes (Figure 8F and Supplemental Table 3).

IPF lung single-cell RNA-Seq identified a fibrotic macrophage archetype with a distinct profibrotic signature (6, 36, 37). We evaluated the expression of a signature of 179 profibrotic macrophage genes that are previously reported upregulated in IPF (6, 36, 37), and we discovered that PNA-33 treatment significantly reduced the expression of 74 of these genes in IPF PCLS (Supplemental Table 4). While *CTSK* (Cathepsin K) is the most downregulated gene after miR33 inhibition in IPF PCLS (*P* = 0004), the expression of multiple important profibrotic genes from this signature is also decreased, including *CHI3L1* (chitinase-3-like protein 1) (*P* = 0.003; fold change, 0.7), *CHIT1* (chitinase 1) (*P* = 0.001; fold change, 0.8), *ITGA8* (integrin subunit α 8) (*P* = 0.005; fold change, 0.9), *ITGB5* (integrin subunit β 5) (*P* = 0.002; fold change, 0.84), *CCL2* and *CCL4* (C-C motif chemokine ligand) (*P* = 0.01; fold change, 0.6; and *P* = 0.03; fold change, 0.54, respectively), *CD36* (cluster of differentiation 36) (*P* = 0.01; fold change, 0.9), and *MERTK* (MER proto-oncogene, tyrosine kinase) (*P* = 0.01; fold change, 0.88) (Figure 8G and Supplemental Table 4).

To validate the ability of PNA-33 to attenuate the IPF macrophage disease signature, we also compared the log fold changes of these genes in IPF PCLS following treatment with PNA-33 to the differentially expressed genes in IPF macrophages from single-cell RNA-Seq data (from 32 IPF lungs and the 28 controls) (37). Our analysis revealed that miR-33 inhibition attenuated multiple genes from the IPF macrophage’s signature list, including *CHI3L1*, *CHIT1, MERTK, CTSK, ITGB5, FN1* (fibronectin 1), *LGMN* (legumain), *LPL* (lipoprotein lipase), *FABP5* (Fatty acid binding protein 5), *LIPA* (lipase A), and *CTSZ* (cathepsin Z) (Figure 8H and Supplemental Table 5).

In summary, the analysis of the ex vivo lung model of fibrosis demonstrated that miR-33 inhibition has potent antifibrotic effects in human lung tissue at the level of gene expression and alters the profibrotic macrophage gene expression in this setting.

## Discussion

Metabolic changes in macrophages are recognized as a key feature in the pathogenesis of chronic lung diseases, including PF (38). Here, we demonstrate that these metabolic alterations can be improved in the absence of miR-33. miR-33 regulates key biological and metabolic functions — including bioenergetics, autophagy, mitophagy, and induction of inflammation in response to injury — in lung macrophages, and the ablation of this miRNA results in improved resolution of fibrosis. Following the observation that levels of miR-33 are substantially increased in BAL cells as well as in monocytes isolated from the patients with IPF, we studied the role of this miRNA in preclinical models of PF in mice. Genetic deletion of miR-33 in the lung macrophages attenuated PF in the bleomycin mouse model, likely via an improvement in mitochondrial homeostasis as well as augmentation of autophagy. Pharmacological inhibition of miR-33 using i.n. delivery of PNA-33 in mice model of PF, as well as the 3D culture of murine PCLS and human IPF, established the possibility of a macrophage-specific miR-33 inhibitor as a therapeutic approach for PF.

The identification of a novel, effective, long-term anti-fibrotic agent that targets the underlying mechanisms of PF is appealing. Individual miRNAs can modulate the expression of multiple mRNA targets and can have broad effects on multiple cellular pathways. For this reason, therapies targeting individual miRNAs can have a broader impact than traditional single-molecule/single-target approaches. Current anti-miRNA technologies are hindered by physiological and cellular barriers to delivery into target cells. Presenting a targeted lung macrophage, miRNA silencing can be a unique and novel therapeutic approach for PF. PNAs are synthetic analogs of DNA composed of nucleobases that are connected to a pseudo-peptide backbone through a carboxymethylene linker (29, 30). The charge-neutral backbone reduces electrostatic repulsion and enables PNAs to hybridize with DNA and bind to single-strand targets with high specificity and affinity. These constructs are also not susceptible to proteases or nucleases, making PNAs ideal molecules for targeting miRNAs (39). We generated PNA-33 as a specific antisense inhibitor of miR-33 and tested the accumulation of this nanoparticle in the lung macrophages after direct delivery to the lung. The strong antifibrotic protection of this system was evident in the animal model of PF as well as the ex vivo 3D culture system from mice and humans.

There is evidence of the vital role of AM in the process of PF, and they have been presented as the chief effector cells of immune responses with both proinflammatory and antiinflammatory properties (40). miR-33 regulates cellular lipid metabolism and mediates the balance of aerobic glycolysis and mitochondrial oxidative phosphorylation to instruct macrophage inflammatory polarization and shape innate and adaptive immune responses (16, 41, 42). The ability of miR-33 to reprogram immune cells has been identified as a player in the osteoprotective actions of anti–miR-33 therapies (43). Our discovery that the antifibrotic effects of miR-33 ablation are mediated through enhanced mitochondrial homeostasis, as well as augmented autophagy and repression of postinjury inflammation, opens the possibility of a novel promitochondrial anti-miRNA therapy for PF. Furthermore, the reprogramming of immune cells via miR-33 inhibition could have important implications for a wide variety of diseases characterized by exaggerated immune responses, such as rheumatoid arthritis and asthma.

There is evidence showing the cellular interaction between macrophages and epithelial cells in the process of lung repair and fibrosis (9). It has been shown that, during homeostasis, AM interact with alveolar epithelial cells to clear apoptotic cells, environmental particulates, and pathogens (44). Herein, we documented that the specific ablation of miR-33 in lung macrophages improves mitochondrial homeostasis in macrophages as well as in AT2s after bleomycin injury. We also demonstrated that these AT2s are protected against cell death. This striking cell-to-cell interaction has an important antifibrotic effect, although the cascades of cellular and mechanistic events behind this interaction are yet to be explored.

Autophagy is one of the basic cellular homeostatic processes induced under conditions of stress, and dysregulation of autophagy impacts numerous human diseases, including IPF (45). It has been shown that autophagy is not induced in total human IPF lungs, and there is a decrease in the number of autophagosomes observed with electron microscopy in IPF lungs (46). The regulatory role of this process depends on the cell type and is decreased in lung epithelial cells from patients with IPF and in murine models of IPF (47). At the same time, it has been shown that augmenting autophagy in fibroblasts results in the resolution of PF (48). These findings demonstrate that autophagy plays a vital yet complicated role in the pathogenesis of these chronic diseases. In this study, we presented a profound increase in autophagosome formation in miR-33–deficient macrophages after bleomycin injury. It has also been shown that autophagy plays an essential role in the inflammatory response of the lung to infection and stress (49). We demonstrated a significant decrease in the inflammatory response in our model along with this augmentation in autophagy.

Mitophagy is a highly specialized form of autophagy that is induced to clear the dysfunctional mitochondria after the injury. Insufficient mitophagy gives rise to the accumulation of damaged mitochondria, and it is implicated in a cell-specific manner in the pathogenesis of IPF (35, 50, 51). Mitochondrial quality control in lung macrophages is a critical determinant of PF. Diminished mitochondrial quality control results in augmented mitochondrial dysfunction and increased mtROS that leads to PF by promoting profibrotic macrophages (52). The absence of miR-33 in the macrophages leads to an augmentation of autophagy as well as mitophagy, which results in the resolution of fibrosis by polarizing the macrophages from profibrotic to antifibrotic.

Macrophages are innate immune cells, and the interplay between proinflammatory/antiinflammatory macrophage phenotypes has been suggested to play a key role in the development and progression of lung fibrosis as well as a key role in inhibiting fibrosis (53–55). The recent findings from single-cell RNA-Seq data from mice and human lung fibrosis revealed the presence of a distinct profibrotic macrophage subtype in the fibrotic lung, with a higher level of *CHI3L1*, *CHIT1*, *APOE*, and integrins (36, 37, 56–58). Inhibiting miR-33 in PCLS isolated from patients with IPF not only decreased the expression of *ACTA2* and collagen, but also altered the expression of genes known to be expressed in profibrotic macrophages, suggesting an effect on this population.

AMPK is a critical cell bioenergetic sensor and metabolic regulator (56). Importantly, previous studies have demonstrated that AMPK activators such as metformin exert protective effects on lung injury (48). In addition to AMPK, PGC-1α is another master regulator of metabolic reprogramming and an inducer of mitochondrial biogenesis, and we previously discovered that thyroid hormone (T3) inhibits lung fibrosis by restoring mitochondrial health and function through a PGC-1α–dependent pathway (11, 13). miR-33 has previously been shown to regulate mitochondrial homeostasis by targeting both *Pgc-1α* and *Ampk* in the context of atherosclerosis (17, 22, 59). The importance of the AMPK/SIRT3/PGC-1α pathway in the regulation of metabolic remodeling is also well known (60, 61). AMPK and PGC-1α may modulate autophagy, apoptosis, inflammation, and mitochondrial function through SIRT3 signaling (62, 63). In this work, we confirmed a significant increase in the expression levels of AMPK, PGC-1α, and SIRT3 in miR-33–deficient macrophages; their downstream effects might be responsible for the resolution of PF via an increase in autophagy, decrease in apoptosis, amelioration of the inflammation, and improvement of mitochondrial homeostasis.

Despite elucidating the role of miR-33 in regulating macrophage immune-metabolic response during lung fibrosis, we did not address several important mechanistic insights in this study. For Instance, we did not explore the role of lipid metabolism and FAO in the pathogenesis of lung fibrosis. The contribution of miR-33 in regulating lipid metabolism is well established (18–23, 26, 42, 64), although the relationship between this concept and lung fibrosis needs to be explored. In the current study, while we provide strong experimental evidence suggesting active crosstalk between macrophages and other cells in the lung such as epithelial cells, the molecular mechanism that mediates these profibrotic responses need to be further elucidated. Finally, many of our analyses were based on the comparison between advanced IPF samples with disease-free controls; miR-33 may have different effects in different stages of the progression of IPF or in other advanced lung diseases. Thus, future studies will need to assess miR-33 and its targets in different stages of IPF and other lung diseases.

Herein, using in vivo and ex vivo models of PF, we demonstrated that genetic and pharmacologic inhibition of miR-33 in macrophages attenuates lung fibrosis via improvement of mitochondrial homeostasis and augmentation of autophagy. Finally, we propose an efficient targeted therapy for PF that could be further evaluated as a therapeutic strategy in humans.

## Methods

Supplemental Methods are available online with this article.

### In vitro experiments

#### Primary mouse AM isolation.

AM was isolated as previously described (65). Briefly, BAL was harvested by slowly injecting and withdrawing 1 mL of PBS into the lung 3 times through a cannula in the trachea. BAL fluid was centrifuged (300g, 5 minutes, 4°C), the cell pellet was washed in PBS, and cells were plated and used for different purposes as described in the appropriate figure legend. For miR-33 inhibition in initial in vitro studies, miR-33 antagomir was purchased from Dharmacon and transfected using lipofectamine RNAiMAX (Thermo Fisher Scientific) according to the manufacturer’s protocols.

#### Cell isolation from human lungs.

Human immune cells were isolated from fresh human lung samples as previously described (66, 67). Briefly, 2–3 g of tissue were used for digestion using (1 mg/mL collagenase/dispase (Roche), 3 U/mL elastase (Worthington), and 20 U/ mL DNAse (Roche) for 30 minutes at 37°C. All CD45^+^ and CD45^–^ cells were isolated using MACS (Miltenyi Biotec) according to the manufacturer’s protocol.

### In vivo experiments

#### Generation of macrophage-specific miR-33–deficient mice.

Generation of miR-33–conditional KO mice (*miR-33^loxP/loxP^*) was accomplished with the assistance of Cyagen Biosciences Inc. The success of this approach has been verified by Southern blotting and confirmed by PCR-based genotyping using specific primers. To generate macrophage/monocyte-specific miR-33–deficient mice, *miR-33^loxP/loxP^* mice were crossed with *LysM^Cre^* mice from The Jackson Laboratory (stock no. 004781).

#### Bleomycin-induced mice model of PF.

miR-33 macrophage-specific–KO mice (*miR-33^M/M^–/–*), and appropriate controls (*miR-33^M/M^+/+*) were used in a bleomycin-induced PF model. Briefly, PF was induced in mice by intrapulmonary delivery of bleomycin (1.5 U/kg) or 0.9% normal saline via oropharyngeal instillation. Mice were euthanized, and lungs were harvested on day 14 for fibrosis analysis. Mice were randomly assigned to groups. Mice of both sexes were studied, and 10–15 mice per group were used. The bleomycin challenge was not blinded, but the analysis of animal samples was. All animal model experiments were performed 3 times.

#### Pharmacological inhibition of miR-33 in mice.

All mice used for this study were WT C57BL/6 (Jackson Laboratories). PF was induced in mice by intrapulmonary delivery of bleomycin (1.5 U/kg) or 0.9% normal saline via oropharyngeal instillation. PNA-33 or scrambled control was administered i.n. (2 mg/kg) on day 7 after the bleomycin injury and repeated every 3 days. Mice were euthanized, and lungs were harvested on day 21 for fibrosis analysis.

### Ex vivo experiments

#### mPCLS.

mPCLS were generated as previously described (68–70). Mouse lungs from both vehicle and bleomycin (day 14) models were used and repeated accordingly. Briefly, low melting grade agarose (3%, weight/volume) was slowly injected via the trachea to artificially inflate the lung. Lungs were cooled at 4°C for 15 minutes to allow jelling of the agarose and then cut to a thickness of 150 μm using a Compresstome (VF-300-0Z by Precisionary) at a cutting speed of 6 μm/s and the oscillation frequency of 5 Hz. The mPCLS were cultured in 24 multiwell plates (Corning) in 500 μL DMEM-F12 no-phenol red containing 0.1% FBS, 1% penicillin/streptomycin 37°C (Sigma-Aldrich), 5% CO_2_, and 95% humidity. Treatment with PNA-33 or scrambled control was administered in the first 24 hours after slicing, all media were changed daily, and lung slices were isolated 5 days after treatment for analysis. Serial live imaging of lung slices by SHG microscopy was performed before (0 hours) and after each treatment (120 hours) (71). mPCLS were fixed with 4% (weight/volume) paraformaldehyde overnight, and paraffin was embedded at 0 hours and 120 hours. Sections (3 μm) were cut using a microtome, mounted on glass slides, and subjected to antigen retrieval. After deparaffinization and rehydration, staining was performed according to standard protocols for Masson’s trichrome. Finally, samples were mounted using a mounting medium and covered with a cover slip. Microscopic scanning of the slides was conducted in bright-field (BF) with a Nikon inverted microscope at 20***×*** magnification. Two representative images were acquired for each sample, and at least 20 different random fields of view were used for collagen quantification. RNA was isolated from 5–6 replicated lung slices from each treatment using miRNeasy mini kit (Qiagen) at 0 and 120 hours for all groups. mRNA levels of fibrogenic genes were quantified by TaqMan quantitative PCR (qPCR) and normalized to GAPDH expression. All experimental groups were performed in a group of 6 technical replicates and repeated at least 3 times.

#### hPCLS.

hPCLS was generated from the lungs of the IPF patients as previously described (69). Briefly, right-middle and lower lung lobes were inflated by injecting 2% warm (37°C) low-melting agarose, and peripheral slices (300 μm) were cut with a vibratome and then punched to a diameter of 4 mm using a biopsy punch (69). Individual PCLS/punches were treated with PNA-33 or scrambled control for 5 days. All IPF PCLS were harvested at the end of the 5 days of treatment and were used for RNA extraction using miRNeasy mini kit (Qiagen) for further analysis.

### RNA-Seq

Total RNA from all IPF PCLS samples was extracted with miRNeasy Mini Kit (Qiagen, 217004) according to the manufacturer’s instructions. The purity of the RNA was verified using a NanoDrop at 260 nm, and the quality of the RNA was assessed using the Agilent 2100 Bioanalyzer (Agilent Technologies). Libraries were prepared using KAPA Stranded mRNA-Seq Kit. mRNA was enriched by ribosomal RNA depletion. Library quality was checked using an Agilent TapeStation analyzer, and sequencing was performed with NovaSeq (2***×***100, 25 million reads per sample). FastQC files were generated and trimmed (https://www.bioinformatics.babraham.ac.uk/projects/trim_galore/), and a quality review was performed by FastQC (http://www.bioinformatics.babraham.ac.uk/projects/fastqc/) before and after quality trim. Reads were aligned with STAR aligner (version 2.6.1d, using the default settings) to the HG38 human genome and ENSEMBL94 annotation GTF file. After alignment and summarization with featureCounts of the Subread package (feature Counts release 1.6.3 and Picard version 2.18.21), data were normalized and differential expression was carried out with edgR package (72) and implemented in R.

For comparing the effect of miR-33 inhibition in IPF PCLS to IPF lung macrophage signature, processed single-cell RNA-Seq data of IPF and control lungs (37) was downloaded from GEO (accession no. GSE136831). Gene expression counts were normalized by scaling values to 10,000 transcripts per cell; they were then normalized by natural log transformation with a pseudo count of 1. IPF and control cells labeled as “Macrophage” (37) were extracted from the entire data set, and average normalized gene expression values of all genes were calculated for each subject (32 IPF; 28 controls); these average per subject values were then used for a 2-tailed Student’s *t* test comparing IPF and control values; the resulting *P* values were adjusted for FDR. Nonribosomal genes with an adjusted *P* value below 0.5 and absolute log fold change greater than 0.07 were used to represent differentially expressed genes in IPF macrophage. Of these 1,197 genes, 1,181 genes were also evaluated via bulk RNA-Seq of IPF PCLS either treated or untreated with miR-33 inhibitor. To evaluate miR-33 inhibition’s ability to attenuate the IPF macrophage disease signature, we compared the log fold changes of these genes in ex vivo IPF PCLS following treatment with miR-33 inhibitor to the reported log fold changes in IPF versus control lung macrophage in vivo.

### Data and materials availability

All data, code, and materials used in the analysis are available to any researcher for purposes of reproducing or extending the analysis. All raw count expression data from ex vivo studies were deposited to the GEO (accession no. GSE215948).

### Statistics

For in vitro and in vivo assays, statistical analysis was performed by GraphPad Prism version 8.1.2. Results were analyzed by Mann–Whitney *U* test for comparisons of 2 groups when sample data were not normally distributed, by unpaired Student’s *t* test for comparisons of 2 groups with normal distribution, by 1-way ANOVA with Student-Newman-Keuls post hoc test for pairwise comparisons of 3 or more groups or more than 10 per group, and by Kruskal-Wallis 1-way ANOVA for comparison of groups with fewer than 10 samples per group followed by Dunn’s post hoc test for pairwise comparisons. Efficacy experiments were designed for 10–15 animals in the control and treated groups, to allow for 82% power to detect a difference of 20% between the 2 groups at a statistical significance level of 0.05, but the actual size of the groups differed because of mortality. All data were presented as (mean ± SEM), and the differences were considered statistically significant at *P* < 0.05. Unless specified in the text, data are expressed as means of at least 3 independent experiments. Fold change for qPCR was determined using the 2^–ΔΔCt^ method (Livak).

### Studies approval

#### Human studies.

Demographic characteristics of the BAL Freiburg cohort (73) used for miR-33 measurements are described in Supplemental Table 1. IPF diagnosis was established by a multidisciplinary board according to the American Thoracic Society/European Respiratory Society criteria and was later determined to be consistent with recent guidelines (74). Healthy age- and sex-matched controls were used from the healthy volunteers, and lung disease was ruled out by a pulmonary function test and clinical examination (73). Demographic characteristics of the IPF explant lung samples used for cell isolation used are described in Supplemental Table 2. Age- and sex-matched healthy lungs were from rejected donor lung organs at the Brigham and Women’s Hospital or from donor organs provided by the National Disease Research Interchange (NDRI). The study protocol was approved by the Partners Healthcare IRB (no. 2011P002419) and the Yale University IRB (no. 2000022618).

#### Animal studies.

All mice were housed and used for the experiments under the direction and approved protocols. All animal studies were conducted in accordance with the NIH guidelines for the humane treatment of animals and were approved by the IACUC of Yale University.

## Author contributions

FA, CFH, and NK conceptualized, acquired funding, and supervised the study. NLP and CFH generated macrophage-specific miR-33–KO mice and provided the necessary guidance for this project. All experiments in this study were performed by FA, NLP, SM, MC, JK, SPP, TB, SD, CCJ, KASR, JEM, NRA, GI, NO, JCS, GD, and TSA. PNA-33 was designed and generated by SM and RB. LS, CR, and CSDC provided guidance and necessary supports for this project. AP, JAVN, and IR provided BAL and human lung tissues from IPF and controls for this study. FA and XL prepared and processed all TEM images. The manuscript was drafted by FA, NLP, RB, CFH, and NK and was reviewed and edited by all other authors.

## Supplementary Material

Supplemental data

## Version 1 01/10/2023

In-Press Preview

## Version 2 02/22/2023

Electronic publication
