# Supplementary material for: microRNA-33 deficiency in macrophages enhances autophagy, improves mitochondrial homeostasis, and protects against lung fibrosis
Source: JCI Insight. 2023 Feb 22;8(4):e158100. doi: 10.1172/jci.insight.158100 (PMC9977502; doi:10.1172/jci.insight.158100)
Supplement: Supplemental data [file jciinsight-8-158100-s255.pdf]

Supplementary Materials.

microRNA-33 Deficiency in Macrophages Enhances Autophagy, Improves Mitochondrial Homeostasis and Protects Against Lung Fibrosis

Authors:

Farida Ahangari[1]†, Nathan L. Price[2,3]†, Shipra Malik[4], Maurizio Chioccioli[1], Thomas Bärnthaler[1,5], Taylor Adams[1], Jooyoung Kim[1], Sai Pallavi Pradeep[4], Shuizi Ding[1], Carlos Cosmos Jr.[1], Kadi-Ann S. Rose[1], John E. Mcdonough[1], Nachelle R. Aurelien[1,6], Gabriel Ibarra[1,7], Norihito Omote[1], Jonas C. Schupp[1], Giuseppe Deluliis[1], Julian Villalba Nunez[8], Lokesh Sharma[1], Changwan Ryu[1], Charles S. Dela Cruz[1], Xinran Liu[9], Antje Prasse[10], Ivan Rosas[8,11], Raman Bahal[4], Carlos Fernández-Hernando[2]†*, Naftali Kaminski[1]†*

MATERIALS AND METHODS

Gene Set Variation Analysis (GSVA).

To measure miRNA activity, we identified miR-33 target genes in two publicly available gene expression datasets, one from lung tissue (GSE47460) and one from BAL (GSE70866). We determined overall miRNA activity by analyzing all target genes using Gene Set Variation Analysis (GSVA) (1). Briefly, GSVA allows for gene set enrichment analysis of miRNA target genes to be analyzed as a group allowing for a more sensitive determination of differential pathway activity compared to other methods.

Hydroxyproline assay.

Lung hydroxyproline was analyzed with a hydroxyproline colorimetric assay kit from Biovision (Biovision K555-100) following the manufacturer's instructions. Briefly, constant weight homogenates from the lungs of control and experimental mice were hydrolyzed in 12 N HCl for 3 hrs at 120°C. The dried digestions reacted with Chloramine T and were visualized by DMAB reagents. The absorbance was measured at 560 nm in a microplate reader. Data were expressed as µg of hydroxyproline/right lung.

Mouse lung tissue histological analysis.

Animal tissue sections (4 µm) were stained with Masson trichrome (collagen/connective tissue), and H&E (Haematoxylin and Eosin stains). Immune staining was performed after paraffin removal, hydration, and blocking, following the recommendation of the manufacturer (ABC detection system from Vector's lab, USA). Microscopy was performed on a Nikon Ti-E (Nikon Instruments, Tokyo Japan) and quantification of collagen was done with ImageJ software (NIH,

Bethesda, MD) using the Image Color RGB split command. The acquisition setup was identical for all images.

RNA Extraction.

miRNA extraction from cells and tissues was done using miRNeasy kits (Qiagen). Cells were washed twice with PBS and lysed using Qiazol reagent (Qiagen, Valencia, CA, USA). 30–50 mg of frozen lung tissue in 700 µl of Qiazol (lysis buffer, Qiagen) according to the manufacturer's instructions. The purity of the RNA was verified using a NanoDrop at 260 nm, and the quality of the RNA was assessed using the Agilent 2100 Bioanalyzer (Agilent Technologies).

Real-time Quantitative Reverse Transcription-Polymerase Chain Reaction for RNA expression.

Relative expressions of messenger RNAs from all *in vitro*, *in vivo,* and *ex vivo* experiments were determined by real-time quantitative reverse transcription-polymerase chain reaction (qRT-PCR) on Viia7 1.0 Real-Time PCR System using TaqMan gene expression assays. Reverse transcription with random primers and subsequent PCR were performed with TaqMan RNA-to-CT one-step kit (Applied Biosystems). Raw data for cycle threshold (Ct) values were calculated using the ViiA7 v.1 software with an automatically set baseline. The results were analyzed by the ΔΔCt method and GAPDH (Glyceraldehyde 3-phosphate dehydrogenase) was used as a housekeeping gene. Fold change was calculated by taking the average over all the control samples as the baseline. All the probes used in this study were purchased from Thermo Fisher Scientific.

miRNA Assay.

miRNA was extracted using miRNeasy kit (Qiagen) as per the manufacturer's protocol. miRNA measurements were performed using TaqMan™ MicroRNA Assays (Thermo Fischer Scientific). cDNA synthesis for both miRNA and the control was carried out using the High-Capacity cDNA Reverse Transcription Kit (Applied Biosystems, Foster City, CA, USA). Real-time PCR was performed using Viia7 1.0 Real-Time PCR system.

Primers were used as follows:

hsa-miR-33a-5p; GUGCAUUGUAGUUGCAUUGCA

hsa-miR-33a; CAAUGUUUCCACAGUGCAUCAC

U6-snRNA;

GTGCTCGCTTCGGCAGCACATATACTAAAATTGGAACGATACAGAGAAGATTAGCA

TGGCCCCTGCGCAAGGATGACACGCAAATTCGTGAAGCGTTCCATATTT

Western Blot.

For Western blot, frozen lung tissues or isolated cells were lysed and homogenized in T-Per or M-Per, respectively (Thermo Fisher Scientific) added phosphatase and protease inhibitor (100 µl per 10 mg tissue, Abcam, ab201119). Protein content was measured with Nanodrop (280nm) and denaturation was performed at 95°C for 5 min in the presence of mercaptoethanol and Laemmli buffer. 20 µg protein per lane was loaded onto a 4-20% gel (bio rad) and samples were run at 50 mA followed by transfer on PVDF membranes using the Trans-Blot Turbo Transfer System (Bio-Rad). Membranes were washed and blocked in 5% dry milk (American Bio-Inc) for 60 min followed by incubation overnight (at 4°C) with primary antibody according to the manufacturer's instruction. Primary antibodies used as follows; PGC-1α, sc-13067, and ab54481;

LC3A/B, ab51520; p62/SQSTM1, ab56416; AMPK, cs2535T, and cs26035, β-actin, sc-47778) obtained from Santa Cruz Biotechnology (sc), Abcam (ab) or Cell Signaling (cs). Signal was detected using appropriate HRP conjugated secondary antibody (1:1000 for 1h at room temperature) using ECL substrate (Bio-Rad). Visualization was performed using an enhanced chemiluminescent detection kit (Bio-Rad, Hercules, CA, USA). Quantification of blots was done by densitometry using Bio-Rad Image Lab Software 5.2.1(Bio-Rad Laboratories) and actin as a loading control.

BAL Cytokine Measurement using Mesoscale Discovery (MSD).

BAL cytokine measurements were performed using Mesoscale Discovery (MSD) platform. We used 100 ul of BAL from all the mice to measure mouse IFN-γ, IL-1β, IL-2, IL-4, IL-10, IL-12p70, IL-13, KC, and TNF-α. Via U-PLEX Biomarker Group 1 (ms) Assays following the manufacturer's protocol.

Seahorse XF96 Extracellular Flux Analyzer.

Cellular metabolism was measured with the Seahorse XFe96 Extracellular Flux Analyzer (Agilent Technologies, Santa Clara, CA) for real-time analysis of extracellular acidification rate (ECAR) and oxygen consumption rate (OCR), as previously described (2). All assays were performed using primary mouse alveolar macrophages at a seeding density of 80,000 cells/well in 200 µl of DMEM in an XF96 cell culture microplate. Cells were switched to unbuffered DMEM supplemented with 2 mM sodium pyruvate and 20 mM carnosine 1 h before the beginning of the assay and maintained at 37°C. OCR was measured after sequentially adding to each well 25 µl of oligomycin (an ATP synthase inhibitor), FCCP (a protonophore), and rotenone and antimycin A

(inhibitors of complexes I and III) to reach working concentrations of 1 μg/ml, 1 μM and 0.5 μM, respectively. OCR is reported in picomoles per minute per 80,000 cells.

mtDNA measurement in BAL.

mtDNA was quantified in cell-free BAL fluid samples from all the mice by amplification of the mitochondria-specific Cytochrome C Oxidase 1 gene (3, 4). Briefly, DNA extraction from mice BAL was performed using DNeasy Blood and Tissue kit Qiagen, Cat. No. 69504) according to the manufacturer's protocol. Mitochondrial DNA present in the samples was assessed by qPCR using LightCycler FastStart DNA Master SYBR Green (Applied Biosystems). The primer sequences were as follows: *Gapdh* (forward, CCTGCACCACCAACTGCTTAG; reverse, GTGGATGCAGGGATGATGTTC), *Cox1* (forward, 5′-GCCCCAGATATAGCATTCCC-3′; reverse, GTTCATCCTGTTCCTGCTCC). To generate the mtDNA standard curve for mtDNA copy quantification (copies per μl), a PCR fragment was cloned from murine-isolated mtDNA containing the mouse cox sequence using Ptgs1 (NM_008969) Mouse Tagged ORF Clone, OriGene.

Immunocytochemistry/Immunofluorescence.

Immunohistochemistry (ICH) was performed as previously described (5). In brief, slides were rehydrated, heat-mediated antigen retrieval was performed in citrate buffer (pH=6, 10 minutes) and samples were incubated with primary antibodies (LC3A/B, ab51520; p62/SQSTM1, ab56416) overnight at 4°C after blocking. The Signal was visualized using the Vectorlabs Immpress reagents with DAB substrate according to the manufacturer's protocol. Photomicrographs were taken on a Nikon microscope. For immunofluorescence (IF) staining,

AF488 and AF594 antibodies were used for the detection of the signal. Simultaneous detection of two rabbit antibodies was performed as previously described (6). In brief, after labeling the first target, blocking steps using serum as well as denaturation (citrate buffer, pH=6, 10 minutes) the staining for the second target was done. To reduce autofluorescence, slides were incubated in TrueView reagent for 3 minutes before mounting with a mounting medium containing DAPI (5). IHC quantification was performed in a blinded fashion by using ImageJ's deconvolution tool (7-9)followed by automated counting of nuclei and DAB-positive cells via the mask and the analyze particle's function. All data are shown as the percentage of total cells.

TUNEL Assay.

Terminal deoxynucleotidyl transferase dUTP nick end labeling (TUNEL) was performed using the in-situ cell death detection kit fluorescein (Roche) according to the manufacturer's instructions. After rehydration, slides were washed, incubated in permeabilization solution (0,1% Triton X in 0,1% sodium citrate) for 8 minutes, and incubated for 60 minutes at 37°C in the reaction mixture. For the detection of apoptotic type 2 cells, pro-SPC-antibody (Merck Millipore #ab3786) was added. To reduce autofluorescence, slides were incubated in TrueView reagent for 3 minutes before mounting with a mounting medium containing DAPI. Photomicrographs were taken on a Nikon microscope. For quantification, nuclei were counted via mask and analyzed particle's function using ImageJ. Briefly, the numbers of positive cells within were counted in a minimum of six randomly selected microscopic fields per lung section. The counts of positive nuclei per field were expressed as a percentage of the total number of nuclei in the same microscopic field. Sections from each of the mice per group were analyzed. Double positive (TUNEL+/SPC+) cells were counted also by a blinded observer.

Transmission Electron Microscope (TEM).

Electron microscopy was performed on mice lung tissues and primary alveolar macrophages as described in Figure Legends. Briefly, mice lungs were inflation-fixed (20 cm H2O) with a 3% solution of glutaraldehyde in cacodylate buffer (0.1 M, pH 7.4) for 1 hour. Blocks (1 mm3) were cut from each lung and immersed in additional fixatives until preparation for routine electron microscopy Cells were fixed using Karnovsky's fixation protocol and processed for TEM. Images were acquired using JEM 1011 TEM microscope (10, 11). Final images were processed using Adobe Photoshop and ImageJ (NIH, Bethesda, MD). The acquisition setup was identical for all images.

Mitophagy Assay.

Primary AM was isolated from WT mice as previously described (12) and seeded in 12 well plates with a cell density of $2x10^5$ per well**.** On day 2, cells were divided into two groups and exposed to bleomycin (15 nM) or saline for 2 hours before treating with PNA-33 (2 nM) or scramble control. On day 3, cells were washed and stained using a Mitophagy detection kit (Dojindo code; MD01), according to the manufacturer's protocol, and imaged using confocal microscopy.

M1/M2 Assay.

Primary AM macrophages were isolated from WT mice as mentioned above and treated with PNA-33 (2 nM) or scramble control 24 hours before exposing them with IL-13 or INF-γ+ LPS. Recombinant mouse IL-13 (413-ML-005/CF) or control were used at the final concentration of 10 ng/ml for another 24 hours before harvesting the cells. Recombinant mouse INF-γ (485-MI)

or control was used at the final concatenation of 10ng/ml for 24 hours plus LPS (Lipopolysaccharides from *Escherichia coli* O127:B8) at the final concentration of 100ng/ml for one hour. All cells were harvested at the end of the time point and used for RNA extraction.

Caspase 3/7 Glo Assay.

Caspase 3/7 activity was detected according to the manufacturer's protocol as previously described (13). Small airway epithelial cells (SAEC, LONZA) were seeded in 96 well plates (9000 cells per well) and allowed to become adherent for 2 days. Subsequently, cells were treated with bleomycin (15 mU/ml) for 6 hours to induce apoptosis. After the removal of bleomycin, supernatants from mir33 inhibitor/ scrambled treated mouse alveolar macrophages were added in a 1:1 ratio with SAEC medium. After 24h, the medium was removed and the SAEC medium with Glo reagent was added 1:1 to the wells. After 45 minutes, luminescence was measured on a Cytation3 BioTek microplate reader.

Synthesis of PNA oligomer.

PNA was synthesized using solid phase synthesis on 4-methyl benzyl hydramine (MBHA) resin and standard Boc-chemistry protocols as reported previously (14). Boc-protected monomers were purchased from ASM Chemicals and Research (Germany) and Boc-cysteine was obtained from Peptide International (Kentucky, USA). Boc-5-carboxytetramethylrhodamine dye (TAMRA) (VWR, Pennsylvania, USA) was conjugated to the N-terminus of PNA using Boc-MniPEG-3 (Peptide Internationals, Kentucky, USA) as a flexible linker. PNA was then cleaved from the resin using a cocktail of trifluoroacetic acid (TFA): trifluoromethane sulfonic acid (TFMSA): m-cresol: dimethyl sulfide (DMS) at a ratio of 6:2:1:1 followed by precipitation using diethyl ether. Further,

purification of PNA was performed using reverse-phase high-performance liquid chromatography (RP-HPLC) and molecular weight was confirmed using matrix-assisted laser desorption/ionization-time of flight spectroscopy (MALDI). PNA was then dissolved in purified water and concentration was measured using UV-vis spectroscopy and an extinction coefficient of 185200 M-1cm-1, calculated by combining the extinction coefficient of individual monomers (Adenine: 13700 M-1cm-1, Guanine: 11700 M-1cm-1, Thymine: 8600 M-1cm-1, Cytosine: 6600 M-1cm-1). The sequence of PNAs synthesized is provided below. Perfect Match PNA 33 (17 mer): 5' ATGCAACTACAATGCAA-Cys-SH-3'. Scramble PNA 33 (17 mer): 5' TACGCTAATCACAAAGA-Cys-SH-3'

Gel shift assay.

The binding of PNA with the target miR-33 was studied according to a previous protocol (15). The custom-designed target miR-33 (1 μM), purchased from Midland Certified Reagent Co (Texas, USA), was annealed with the PNA at different ratios in buffer simulating physiological conditions (10 mM NaPi, 150 mM KCl, 2 mM MgCl2). The samples were run on a 10% nondenaturing polyacrylamide gel using tris/boric acid/EDTA (TBE) buffer at 120V for 35 minutes. Further, the bound and the unbound fraction of target miR-33 was visualized by staining the gel with SYBR-gold (Invitrogen, USA) for 2 minutes followed by imaging using Gel Doc EZ Imager (Bio-Rad, USA).

Supplemental Figures.

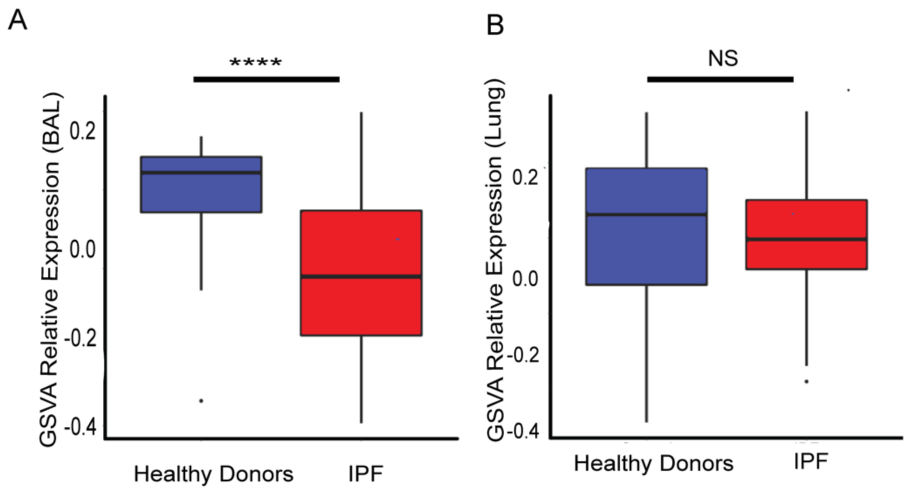

Figure S1. miR-33 target gene expressions decrease in IPF BAL.
(**A**) Gene Set Variation Analysis (GSVA) of miR-33 3-p targets in BAL dataset (GSE70866) 212 IPF patients and 20 healthy donors. (**B**) Gene Set Variation Analysis (GSVA) of miR-33 3-p targets in Lung LTRC dataset (GSE47460) 254 IPF patients and 108 healthy donors. All data is presented as Mean+/-SEM. **** indicates P ≤ 0.0001.

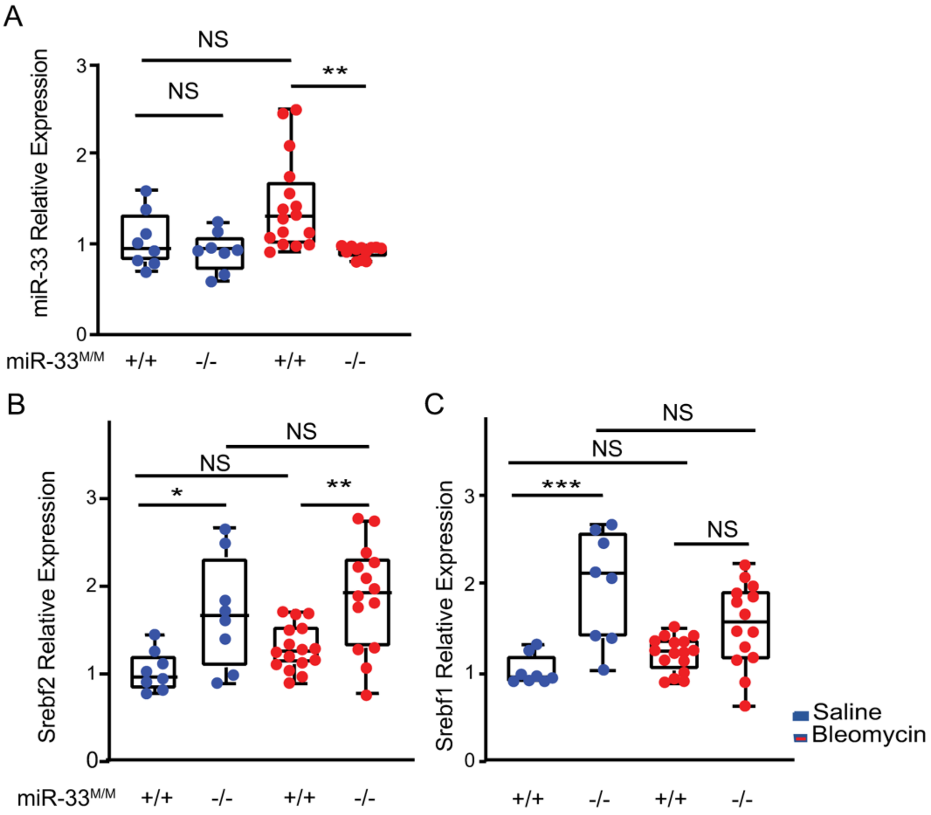

Figure S2. Characterization of miR-33, *Srebp1,* and *Srebp2* expression levels in lungs isolated from the macrophage-specific miR-33 deficient and control mice.
(**A**) miR-33 relative expression in macrophage-specific miR-33 knockout mice ($miR33^{M/M}$-/-) vs controls ($miR33^{M/M}$+/+) in bleomycin (red) compared to saline (blue) n=8 for saline and n=16 for bleomycin groups. (**B** and **C**) miR-33 host genes (*Srebf1* and *Srebf2*) relative expression of $miR33^{M/M}$-/- vs $miR33^{M/M}$+/+ in bleomycin (red) compared to saline (blue) n=8 for saline and n=16 for bleomycin groups. All data were analyzed by ANOVA or Kruskal-Wallis tests followed by posthoc analysis and is presented as Mean+/-SEM. * P ≤ 0.05, **P <0.01, [illegible]

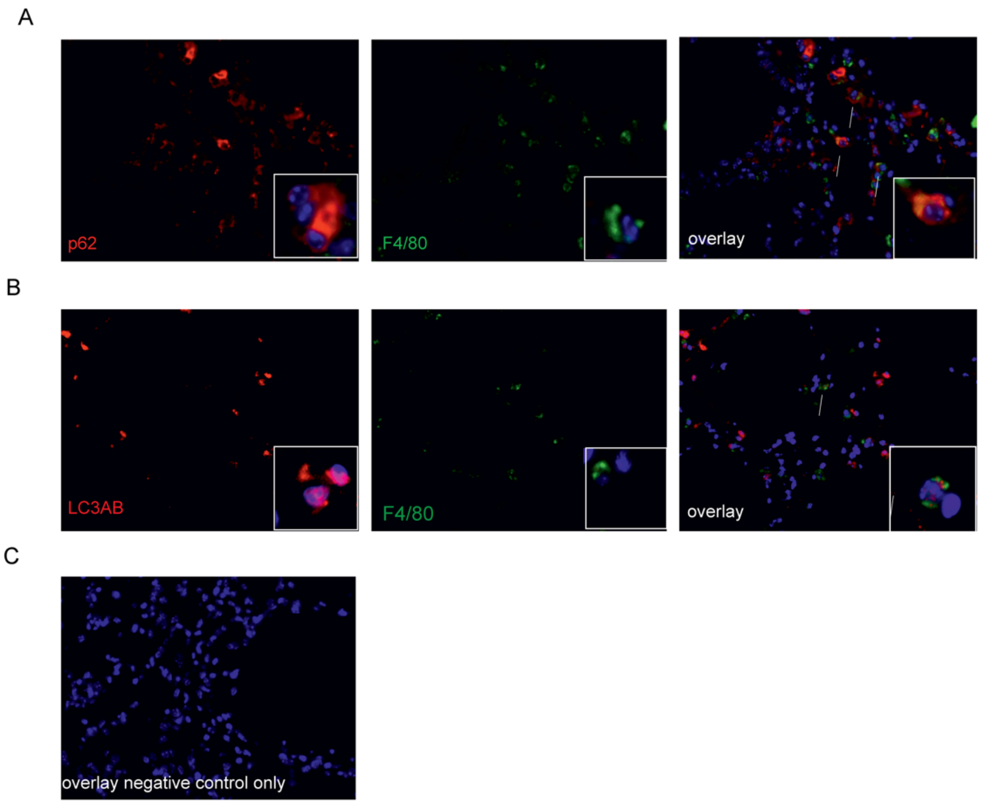

Figure S3. Co- Immunofluorescence (Co-IFC) of LC3A/B and P62 with F4/80 in mouse lung sections
(**A**) Representative images of Co-IFC staining of LC3A/B (red) and F4/80 (green) in lung tissues isolated from miR33$^{M/M}$-/-. (**B**) Representative images of Co-IFC staining of P62 (red) and F4/80 (green) in lung tissues isolated from miR33$^{M/M}$-/-. (**C**) negative control.

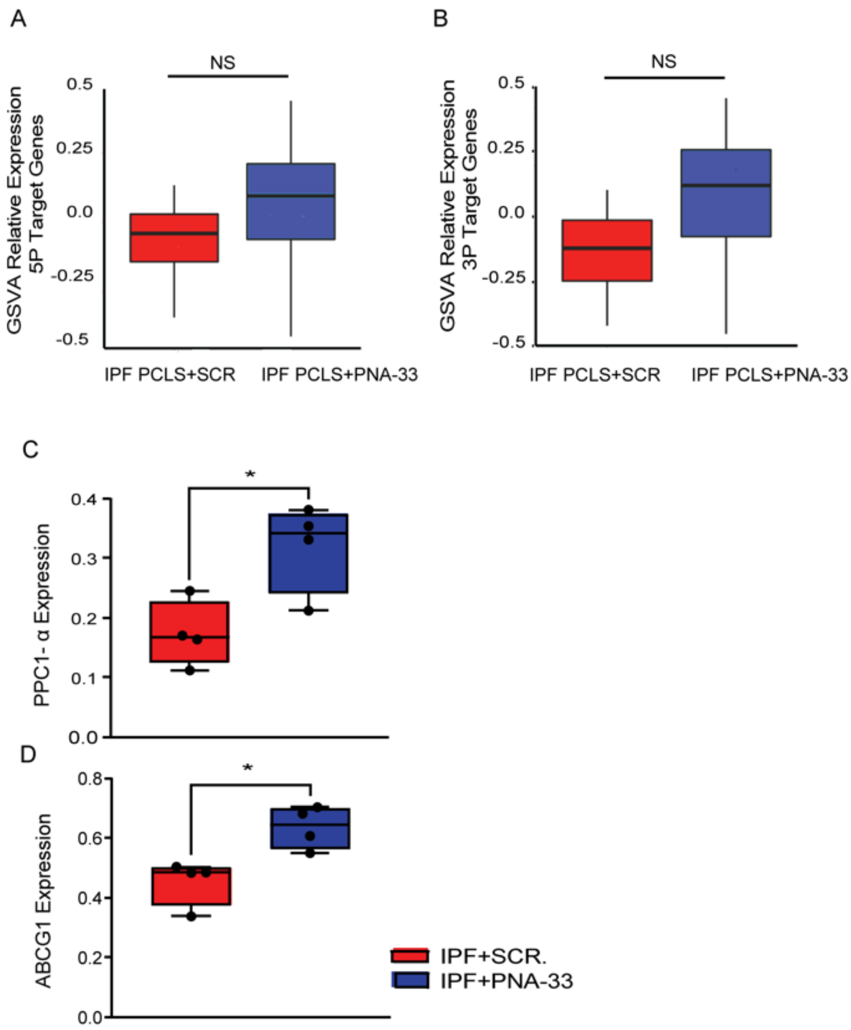

Figure S4. The effect of PNA-33 versus scramble control on the expression of miR-33 target genes in IPF PCLS.
(**A**) Gene Set Variation Analysis (GSVA) of miR-33-Tragets 5p. (P-Value=0.7, F.C.=1.2.) (**B**) Gene Set Variation Analysis (GSVA) of miR-33 targets 3p (P-Value=0.7, F.C.=1.2.). (**C**) The expression of *PPARGC1A* in IPF PCLS treated with PNA-33 versus scramble (P-value=0.01). (**D**) The expression of *ABCG1* in IPF PCLS treated with PNA-33 versus scramble (P-value=0.01).

**Characteristics:**	**IPF- Freiburg**<br>**N=62**
**Age-Year**	**67.4 ± 9.1**
**Sex-%**	**Male-85**
**FVC percent predicted value -%**	**66 ± 20**
**Smoking Status**	**Never smoked -% 42 ± 30**<br>**Former smoker-% 56 ± 70**<br>**Current smoker-% 2**
**Bronchoalveolar Lavage (BAL)**	**Cell count –×106 cells 12.1 ± 7.2**<br>**Alveolar macrophages -% 71 ± 17**<br>**Lymphocytes -% 11 ± 9**<br>**Neutrophils -% 12 ± 14**<br>**Eosinophils -% 4**

Table S1. Clinical and demographic data of IPF (BAL) human samples.

Baseline Characteristics of IPF Patients used for BAL cell isolations. Plus/minus values are means ±SD unless otherwise indicated. FVC denotes forced vital capacity.

Characteristics:	IPF- Explant lungs<br>N=9
Age-Year	66 ± 6
Sex-%	Male-75
FVC percent predicted value -%	42 ± 3
Smoking Status	Non-Smoker -% 37.5<br>Smoker-% 62.5

Table S2. Clinical and demographic data of IPF (lung) human samples used for lung cell isolation.

Baseline Characteristics of IPF Patients used for lung cell isolations. Plus–minus values are means ±SD unless otherwise indicated. FVC denotes forced vital capacity.

Gene	P-Value	Fold Change	Adj. P-Value
COL14A1	0.003657057	0.913686468	0.097513957
COL27A1	0.00436397	0.836792033	0.097513957
COL16A1	0.006588107	0.725660967	0.097513957
COLCA2	0.006882934	0.866285127	0.097513957
ACTA2-AS1	0.007093664	0.666475286	0.097513957
COLEC11	0.008283174	0.872105841	0.097513957
ACTA2	0.010441251	0.739358472	0.097513957
COL8A2	0.010496326	0.841627257	0.097513957
COL24A1	0.010744459	0.951878734	0.097513957
COL6A4P2	0.010811781	0.835012578	0.097513957
COL4A4	0.011702493	0.857330048	0.097513957
COL6A6	0.012291893	0.827629148	0.097513957
COL6A5	0.014543633	0.848601582	0.097513957
COL5A1	0.01543851	0.854025968	0.097513957
COL6A4P1	0.017324939	0.779901084	0.097513957
COLGALT2	0.017826553	0.869872811	0.097513957
COL21A1	0.018568542	0.978401426	0.097513957
COL9A2	0.018928193	0.719679166	0.097513957
COL11A2P1	0.019483983	0.618700077	0.097513957
COL9A1	0.020616238	0.843752613	0.097513957
COL22A1	0.021754537	0.887309823	0.097662183
COL25A1	0.021796439	0.965377346	0.097662183
COL19A1	0.025804708	0.937210203	0.098552275
COL4A3	0.027774553	0.905153236	0.099381344

COL23A1	0.028343011	0.957836919	0.09966254
COL15A1	0.031035743	0.869613895	0.101629223
COL4A6	0.032382904	0.9490343	0.102859656
COL11A2	0.032695671	0.668134255	0.103058152
COL7A1	0.038094407	0.777578416	0.10776195
COLEC10	0.038648521	0.927896261	0.108290082
COL8A1	0.038769142	0.91119589	0.10834367
COL25A1-DT	0.039902134	0.710033721	0.109608391
COL20A1	0.04186542	0.777701121	0.111360997
COL9A3	0.042809652	0.813016257	0.112111707
COL26A1	0.04326319	0.969645156	0.112542376
COL5A3	0.044168657	0.793338223	0.113290577
COL11A1	0.044999981	0.913747535	0.114041642
COL28A1	0.048558429	0.929652628	0.118171044
COL4A2-AS2	0.049266558	0.716957003	0.119012003
COL13A1	0.049448399	0.866451404	0.1191535
COL2A1	0.054345805	0.69599097	0.124294171
COL18A1-AS2	0.057719316	0.75751083	0.12808448
COL5A1-AS1	0.059317838	0.810730854	0.130036864
COL5A2	0.089344476	0.895051102	0.164329423
COL18A1-AS1	0.090621347	0.781881917	0.165887854
COLCA1	0.091147092	0.816081081	0.16652523
COL12A1	0.106596352	0.849721426	0.184515306
COLGALT1	0.110945195	0.777220719	0.189453818
COL10A1	0.151515426	0.962512451	0.234912723
COL4A2-AS1	0.181830072	0.860899925	0.268602533
COL18A1	0.21089781	0.927396749	0.30112969

COL6A1	0.255057575	0.706505359	0.350206284
COL4A5	0.354913433	0.981838547	0.451802002
COL4A2	0.391964864	0.91360773	0.488730841
COL1A1	0.427867054	0.67248122	0.524709939
COL6A2	0.486329594	0.772790235	0.582902954
COL1A2	0.515798823	0.781192217	0.612428505
COL6A3	0.770992387	0.912478394	0.84942215
COL4A1	0.825815793	0.977867983	0.897943027
COL17A1	0.923917837	1.008812734	0.981502295
COL3A1	0.939784375	0.966507305	0.994335237
COLEC12	0.98285696	0.999337305	1

Table S3. Differentially expressed genes (*ACTA2* and Collagen genes only) comparing IPF-PCLS + miR33 inhibitor (PNA-33) Vs. IPF-PCLS + Scramble Control.

Genes	P-value	F.C.	Adj. P-value
CTSK	0.0004	0.5654	0.0975
SLC1A3	0.0005	0.7703	0.0975
MT-ND3	0.0012	0.6965	0.0975
ATOX1	0.0012	0.7156	0.0975
PGK1	0.0016	1.3798	0.0975
SDS	0.0016	0.6995	0.0975
LGMN	0.0016	0.5757	0.0975
LAP3	0.0018	0.6631	0.0975
MNDA	0.0018	0.6290	0.0975
VAMP5	0.0023	0.5877	0.0975
LDHA	0.0024	1.5169	0.0975
CD84	0.0027	0.6301	0.0975
PLEK	0.0028	0.5358	0.0975
TNFSF13B	0.0030	0.7673	0.0975
CD48	0.0031	0.7765	0.0975
CHI3L1	0.0033	0.4880	0.0975
ADGRE5	0.0036	0.7064	0.0975
APOC1	0.0038	0.3632	0.0975
NR1H3	0.0061	0.7249	0.0975
SMIM3	0.0074	0.8024	0.0975
SGK1	0.0075	0.7918	0.0975
GBP1	0.0076	0.5670	0.0975
PALLD	0.0077	0.8718	0.0975
FYB	0.0080	0.8556	0.0975
CDC42	0.0090	0.8551	0.0975

LILRB4	0.0095	0.6768	0.0975
EVL	0.0099	0.8517	0.0975
FCGR2B	0.0107	0.7527	0.0975
S100A9	0.0110	4.8843	0.0975
MERTK	0.0113	0.8839	0.0975
ATP6V1B2	0.0116	0.7910	0.0975
FCGR3A	0.0120	0.6059	0.0975
MT-ATP6	0.0124	0.6711	0.0975
FGL2	0.0129	0.7042	0.0975
NPC2	0.0131	0.8496	0.0975
MMP7	0.0144	1.5333	0.0975
HMGN1	0.0147	1.2931	0.0975
CCL2	0.0149	0.5970	0.0975
NHSL2	0.0159	0.9391	0.0975
CD36	0.0159	0.9053	0.0975
CD4	0.0166	0.8039	0.0975
C15orf48	0.0175	1.1835	0.0975
BCAT1	0.0180	0.8607	0.0975
PPT1	0.0186	0.7457	0.0975
CTSL	0.0190	0.7232	0.0975
CHIT1	0.0195	0.8125	0.0975
FPR3	0.0198	0.7632	0.0975
ANXA2	0.0198	1.3635	0.0975
HCK	0.0202	0.8290	0.0975
MATK	0.0203	0.7275	0.0975
TMEM176B	0.0204	0.6485	0.0975
PLAC8	0.0226	1.3391	0.0977

MS4A6A	0.0229	0.6891	0.0977
MT-ND6	0.0239	0.6292	0.0980
ABHD2	0.0259	0.9042	0.0986
CSF1R	0.0260	0.8318	0.0986
IGSF6	0.0262	0.6440	0.0986
LITAF	0.0274	0.8317	0.0991
APOE	0.0297	0.5768	0.1006
CRIP1	0.0300	0.7063	0.1007
ITGB8	0.0313	1.2600	0.1019
LST1	0.0314	0.5417	0.1019
S100A8	0.0320	7.0417	0.1026
GPNMB	0.0335	0.7171	0.1038
CCL4	0.0350	0.5434	0.1049
PLA2G7	0.0353	0.7770	0.1052
GM2A	0.0365	0.7410	0.1064
TPP1	0.0388	0.7543	0.1084
IGHG3	0.0391	0.6021	0.1087
WARS	0.0427	0.9476	0.1120
SLA	0.0428	0.8530	0.1121
FABP1	0.0449	0.5901	0.1139
GLIPR1	0.0473	0.7679	0.1165
RALA	0.0476	0.8253	0.1169
NRP2	0.0500	0.8598	0.1198
IGHG1	0.0507	0.3218	0.1204
HLA-DQA2	0.0518	0.7711	0.1214
A2M	0.0533	0.7454	0.1231
SLC16A10	0.0593	0.9591	0.1300

KIAA0930	0.0594	0.7320	0.1301
IGHG4	0.0607	0.5983	0.1317
LIPA	0.0617	0.8060	0.1327
LHFPL2	0.0619	0.9022	0.1330
FCGR2A	0.0647	1.1493	0.1360
ITGAX	0.0662	0.7027	0.1376
TGFBI	0.0681	1.4634	0.1398
MMP9	0.0716	0.6436	0.1438
CTSZ	0.0765	0.7327	0.1494
IGKC	0.0789	0.3176	0.1521
FDX1	0.0817	0.8418	0.1556
ITGB2	0.0819	0.7999	0.1557
FXYD5	0.0891	0.7450	0.1641
CMTM3	0.0942	0.7744	0.1703
FUCA1	0.0971	1.1607	0.1739
ITGAM	0.1023	0.8935	0.1798
SPP1	0.1041	1.3908	0.1818
GSN	0.1068	1.1833	0.1848
CYBB	0.1138	0.7770	0.1928
IGLC2	0.1168	0.4406	0.1961
CD9	0.1172	1.2218	0.1966
LSP1	0.1191	0.8224	0.1989
MT-ATP8	0.1264	0.6940	0.2071
STAT1	0.1317	0.8470	0.2131
IGF2R	0.1352	0.8411	0.2167
ACP2	0.1410	0.9422	0.2230
LPL	0.1492	0.8782	0.2324

SCGB3A1	0.1535	4.0443	0.2372
TREM2	0.1562	0.7694	0.2401
EMP1	0.1574	0.8478	0.2415
CMTM6	0.1624	1.1010	0.2470
FKBP1A	0.1677	0.8701	0.2528
LASP1	0.1694	0.7882	0.2546
MT-CO3	0.1731	0.7852	0.2589
HLA-A	0.1765	0.9030	0.2626
MARCKS	0.1830	0.8392	0.2699
PSAP	0.1832	0.7961	0.2701
ARHGDIB	0.1860	0.7905	0.2733
TMSB10	0.1971	1.1295	0.2855
SOD2	0.2171	0.9368	0.3080
TTYH3	0.2185	0.7825	0.3097
TGM2	0.2189	0.8046	0.3101
MS4A4A	0.2214	0.9365	0.3129
DPYSL2	0.2272	0.8721	0.3195
LCP1	0.2347	0.8832	0.3278
C6orf62	0.2406	1.1840	0.3343
SPARC	0.2457	0.7606	0.3397
ATP13A3	0.2555	1.1115	0.3507
PEA15	0.2569	0.8798	0.3522
OLR1	0.2621	1.0982	0.3578
RNASE1	0.2738	0.9012	0.3700
MAFB	0.2771	0.8105	0.3734
COTL1	0.2848	0.8511	0.3815
CD14	0.2926	0.8135	0.3899

ITGB1	0.2963	1.1265	0.3937
FABP5	0.3075	0.8166	0.4056
TPM3	0.3292	0.8785	0.4285
TLN1	0.3346	0.8024	0.4343
SAMHD1	0.3379	0.9156	0.4378
VMP1	0.3380	1.0739	0.4379
HCST	0.3522	0.8664	0.4518
RGS1	0.3961	0.8718	0.4929
CTSB	0.4077	0.8923	0.5046
WASF2	0.4080	0.9147	0.5048
PDIA4	0.4268	1.0937	0.5237
CD44	0.4517	1.0970	0.5490
P4HB	0.4654	1.1530	0.5626
ABCA1	0.4773	0.9591	0.5742
HIF1A	0.4824	1.0752	0.5791
RAB31	0.4926	1.0269	0.5892
TUBA1B	0.4976	0.8963	0.5942
GPC4	0.5194	1.0106	0.6157
CYFIP1	0.5249	0.9393	0.6211
CLTC	0.5261	1.0873	0.6223
BASP1	0.5583	0.9740	0.6535
UCP2	0.5794	0.9289	0.6734
CFL1	0.6118	0.9431	0.7042
SRSF2	0.6142	1.0604	0.7066
CAP1	0.6368	1.0505	0.7282
CCL18	0.6484	1.1910	0.7393
FN1	0.6498	0.8653	0.7406

TIMP1	0.6634	1.1169	0.7530
ACTG1	0.6833	1.0777	0.7711
SRGN	0.6917	0.9505	0.7788
SH3BGRL3	0.7148	0.9440	0.8000
FLNA	0.7211	0.8861	0.8056
ACTB	0.7261	0.9237	0.8099
IL7R	0.7715	0.9760	0.8499
IL1RN	0.7802	0.9699	0.8573
TMSB4X	0.8345	0.9846	0.9055
CALM3	0.8359	0.9765	0.9067
QSOX1	0.8695	0.9759	0.9357
ARID5B	0.8709	0.9931	0.9370
SDC2	0.8716	1.0109	0.9375
TYMP	0.8785	0.9808	0.9435
CALR	0.8819	0.9782	0.9463
ZFP36L1	0.9677	0.9878	1.0000
TPM4	0.97078	0.99609	1
CPM	0.98923	0.99959	1

Table S4. A signature containing 179 profibrotic macrophage genes which are previously reported in IPF (16-18). PNA-33 treatment significantly reduced the expression of 74 of these genes in IPF PCLS.

Gene	LogFC (PCLS)	LogFC (S.C.)	P-value (PCLS)	P-value (S.C.)
TMEM234	-0.420607295	0.008737295	4.48E-05	0.011316413
LILRB4	-0.860353049	0.168172791	0.000185872	4.04E-05
LINC01556	-1.034366191	0.001029855	0.000208721	0.019615969
TRG-AS1	-0.290923526	0.028039102	0.000308948	0.014790454
HTRA4	-0.542845733	0.033547026	0.000326916	0.049560078
LINC01857	-0.710758362	0.02547594	0.000354584	0.002617068
CTSK	-0.822638154	0.19991798	0.000417663	3.21E-06
SLC1A3	-0.376488978	0.530620448	0.000468495	2.70E-05
CEP128	-0.073632849	0.08781842	0.00102685	0.032113583
CDC20P1	-0.639192124	0.000117513	0.001122474	0.039495537
PHGDH	-0.400455259	0.004775575	0.001198979	0.030887618
TMEM177	-0.455805208	0.001992995	0.00122758	0.033784199
KCNJ5	-0.658293652	0.029879264	0.001318167	0.003309044
EMP3	-0.647480948	0.350267927	0.001361789	0.001036653
MEAF6	-0.328107305	0.068036778	0.00157783	8.00E-05
LGMN	-0.796532275	0.26511484	0.001626568	0.00163986
MTMR11	-0.523062641	0.022758292	0.001706565	0.016945668
GNG5	-0.24222836	0.335672209	0.00185478	5.48E-06
BAALC-AS2	-0.280877564	0.003527678	0.001911177	0.000996656
LINC01160	-0.665263231	0.000346914	0.001943808	0.037086849
VAMP5	-0.766797557	0.045880277	0.002288661	0.028478573
KAT8	-0.409265073	0.050846091	0.002309202	0.019802414
OPN1SW	-0.858978785	0.008510073	0.00251618	0.001637572

MT-CYB	-0.732036401	0.332578336	0.002597274	0.000446885
SMIM19	-0.246799807	0.055675717	0.002694579	0.001930638
CD84	-0.666311613	0.181686876	0.002734616	2.70E-05
APOBEC3C	-0.430925304	0.041324399	0.002794393	0.034002969
LINC01705	-0.157235321	0.022409245	0.002795357	0.00013993
MINCR	-0.807183682	0.006802415	0.002803298	0.006756917
EBAG9	-0.307606647	0.026383052	0.002982817	0.00943442
TNFSF13B	-0.382184384	0.116045488	0.003014721	0.000974479
TARBP2	-0.560229521	0.010847747	0.003020918	0.006268798
ABCA6	-0.433976198	0.008833057	0.003047022	0.008459071
TNFAIP8L3	-0.127473915	0.002375389	0.003135903	0.042796605
THRA	-0.39011259	0.01984391	0.003223166	0.004384822
CLEC11A	-0.626665753	0.051390802	0.003229214	0.001640175
CHD1L	-0.06888536	0.041141667	0.003306139	0.042866087
CHI3L1	-1.035157545	0.164190565	0.003329363	6.41E-05
PRXL2B	-0.518789741	0.016793399	0.003481347	0.000724368
KCNMB1	-0.606977835	0.025291871	0.003495855	0.000131897
RCCD1	-0.471019594	0.008676462	0.003528368	0.007029126
ZMIZ1-AS1	-0.184707557	0.049043273	0.003542878	4.12E-06
CORO6	-0.545493839	0.002077292	0.003558868	0.002037007
SLC35C1	-0.421367409	0.008895522	0.003561384	0.034830468
TRAF5	-0.318038848	0.036086545	0.003582041	1.63E-06
ARHGEF9	-0.143580592	0.011960975	0.003646253	0.04913075
ADGRE5	-0.501467623	0.239124574	0.003649666	0.008187866
EHMT2	-0.536460038	0.010688339	0.00365538	0.04909033

APOC1	-1.461164667	0.674173294	0.003762369	0.015720733
FAM20C	-0.220568824	0.068544654	0.003785097	0.034454387
DYRK4	-0.467670444	0.016483261	0.003834709	0.026545016
CDCA7L	-0.246190742	0.007608281	0.003865097	0.009357412
THY1	-0.986195142	0.002062947	0.003885367	0.029246899
UBE2L6	-0.400745422	0.080144467	0.003968135	0.001332464
DHRS4-AS1	-0.121825261	0.019067602	0.004181786	0.012723744
SH3RF3	-0.067236599	0.242061555	0.004307768	5.51E-07
ARMC10P1	-0.907218968	0.004757862	0.004346763	0.039753655
SMIM2	-0.106020254	0.000335461	0.004362355	0.011723418
LY86	-0.166239956	0.097250623	0.004392602	0.008466663
LRRN4	-0.486740818	0.002428316	0.004497658	0.023811039
ADCK2	-0.223749987	0.02007843	0.004670504	0.004094997
TFAMP2	-0.791304781	0.000144294	0.004700981	0.045711006
GBA	-0.611738213	0.033859102	0.004706169	0.025285511
EXTL2	-0.339376421	0.008047448	0.00473024	0.049517245
NHSL1	-0.095721593	0.171475424	0.00492933	0.018244856
BTBD19	-0.614548579	0.053316794	0.004954418	0.001094519
KCTD17	-0.501040487	0.013453409	0.004971831	0.024315978
BTK	-0.300158564	0.042803048	0.005053133	0.002660775
IRF8	-0.529327718	0.084903519	0.005148752	0.031614914
DPP4	-0.391913048	0.015079543	0.00515861	0.000123693
LY9	-0.653557238	0.014853306	0.005174408	3.16E-05
C1QTNF8	-0.432367838	0.000166153	0.005254365	0.049062908
RN7SKP161	-1.145679371	0.000606803	0.0052547	0.041862491

HFE	-0.401442727	0.010748391	0.005320162	0.000923425
SLC29A3	-0.274563479	0.033416955	0.005451326	0.019108372
TMEM251	-0.622254292	0.050915995	0.005471879	0.03821599
ZNF32	-0.16788847	0.006387843	0.005523268	0.011327402
BOD1	-0.334538955	0.015744758	0.005564039	0.006313753
MIR3945HG	-0.40282254	0.080924308	0.005731888	0.045766612
CCDC43	-0.19726907	0.017829635	0.005857496	0.000313564
MTCO3P5	-1.148058459	0.001006092	0.00592337	0.044457952
SDCBP	-0.334360104	0.159588871	0.005970807	0.037088593
TRDC	-0.689548168	0.000712862	0.006066704	0.027772418
TROAP	-0.531491297	0.004865151	0.006106703	1.52E-06
NR1H3	-0.464199132	0.114428338	0.00611876	1.26E-06
DYNLRB1	-0.272197192	0.113522413	0.006161321	0.003659332
CD53	-0.501835905	0.096804378	0.006254436	0.039911459
SULT1C2	-0.605284244	0.015669165	0.006298991	0.001980029
CFAP20	-0.290871093	0.011438207	0.006349676	0.008082248
KLHL8	-0.122732682	0.026691588	0.006360035	0.010421904
CCR5	-0.875702011	0.014962018	0.006381583	0.012360168
TBRG1	-0.332066796	0.074513012	0.006517014	0.016156079
SRGAP2	-0.166491606	0.213105919	0.006554836	0.021848657
PGA3	-0.718070149	0.00461323	0.006580043	0.049618414
CSNK2B	-0.228595802	0.094351856	0.006614505	0.001473023
CRTAM	-0.445525423	0.024105329	0.006621852	0.000208319
GMPR	-0.327364067	0.024899872	0.006635935	0.008598772
B2M	-0.301547363	0.45453599	0.006746368	0.003279319

CD37	-0.675309144	0.114094223	0.006937159	0.026487538
CCNB1IP1	-0.294140768	0.013379673	0.006959232	0.008721732
GPX8	-0.500399904	0.001973743	0.006993764	0.001195142
KCNA2	-0.410734922	0.031429787	0.007050084	0.000158082
AFDN-DT	-0.85624347	0.000235727	0.007122716	0.022648018
CCR1	-0.699442837	0.10884572	0.007231469	6.41E-05
SHOX2	-0.633746404	0.007289357	0.007388527	0.000859981
B3GALT4	-0.421846968	0.0146744	0.007391003	0.001699962
SMIM3	-0.317558122	0.271253863	0.007441763	1.97E-05
TRIM32	-0.368372455	0.003651549	0.007464497	4.69E-05
DUSP12	-0.32825922	0.015976021	0.007487981	0.001435531
POLR1E	-0.410801441	0.006335799	0.007535991	0.004341184
SGK1	-0.336749695	0.466439394	0.007538419	1.65E-06
KLHDC9	-0.822592629	0.00148496	0.007557373	0.000897346
ERAP2	-0.332752354	0.033089786	0.00757859	0.035964798
ISCU	-0.343449646	0.136936593	0.007617201	0.003528238
MT-ND4L	-0.428369157	0.192509115	0.007810107	0.012722673
CD40	-0.369189432	0.046622443	0.007847226	0.00022565
CYP19A1	-0.203045253	0.002776385	0.007892106	0.037577123
SCIN	-0.235840213	0.029687876	0.007966127	0.000911703
TSEN15	-0.248122218	0.012308011	0.008072101	0.045226645
DCLK2	-0.086432229	0.010921862	0.008118371	0.017054315
FCGR1A	-0.619968477	0.094686292	0.008178868	0.007021508
ZKSCAN3	-0.462977204	0.00289858	0.008303768	0.002341293
PSME1	-0.286649502	0.089366282	0.008308675	0.021420951

HIRIP3	-0.824776574	0.01067218	0.008322286	5.75E-05
KLLN	-0.780494368	0.002322014	0.008403606	0.000550018
EMC7	-0.291133026	0.05857526	0.008432214	0.001685726
DCSTAMP	-0.555860899	0.018805756	0.008456701	0.008089273
SNPH	-0.196398937	0.002213942	0.008457748	0.001372647
PDE6G	-0.389829112	0.006072119	0.00846602	0.022964018
SS18L2	-0.285528723	0.039218989	0.008466078	0.008250989
VSIG2	-0.563064855	0.001705784	0.008491183	0.003232646
SF3A3P2	-1.358970274	0.000540435	0.008540192	0.049517245
PMFBP1	-0.405651808	0.015043299	0.008551568	0.009871039
JADE1	-0.243733797	0.033283183	0.008556417	2.48E-05
BABAM1	-0.367590229	0.047503742	0.008556882	0.001244482
ATP6V1C1	-0.231300608	0.056910434	0.008643269	0.019350941
RARRES1	-0.098367115	0.071775366	0.008663558	3.72E-05
PIGT	-0.376634369	0.040788687	0.008704163	0.002730595
KLHL6	-0.356561012	0.090459783	0.00878771	0.043929821
PGAM1P8	-0.621344933	0.006248124	0.00885744	0.024139217
APH1B	-0.283059103	0.068363142	0.00891902	0.000809253
FDX1P1	-1.042944423	0.110494549	0.00895351	1.29E-06
THRB	-0.088106563	0.098380015	0.008978544	2.74E-05
CSPG4	-0.285351983	0.011428756	0.008991543	1.37E-06
RNASEL	-0.457932499	0.009322266	0.009181777	0.014245269
RNASEK	-0.563969267	0.24127564	0.009239249	0.011120103
LINC01281	-0.22613607	0.000680411	0.009301899	0.024280688
IMP3	-0.214146035	0.039625589	0.009383098	0.001662548

FBXO7	-0.232021886	0.033790046	0.009487207	0.006451932
COMMD9	-0.570198515	0.049443821	0.009593174	0.00766107
RTL8A	-0.313052158	0.025168236	0.009680162	0.000130149
GALM	-0.20036503	0.093751824	0.009731897	0.001647652
LMCD1	-0.247027458	0.00262517	0.009738989	0.005038494
CYP27A1	-0.633740627	0.225401737	0.00985102	0.017818016
HPS1	-0.371003292	0.050852213	0.009949112	0.004092157
CHST13	-0.22401247	0.010405479	0.009969038	0.021638191
FKBP5	-0.168807255	0.475776221	0.010005732	0.033395166
TMEM121B	-0.839136372	0.002018824	0.010120208	0.037442856
PARP1	-0.46141556	0.038650581	0.010166248	0.00098365
SH3RF3-AS1	-0.730473651	0.001018513	0.01021707	0.001499837
DNAJC30	-0.695616173	0.010580292	0.010302451	0.002184223
DLGAP1-AS1	-0.665721178	0.016420821	0.010339418	0.004930912
ZBED5-AS1	-0.214985038	0.029111866	0.010465268	0.000263289
GPR150	-0.772539561	0.005887551	0.01048809	0.008774066
COL8A2	-0.248746666	0.049189336	0.010496326	6.06E-05
MORN4	-0.230808254	0.002783248	0.010514446	0.000288128
CCNQ	-0.298552512	0.02235601	0.010517379	0.000350076
SNAPC2	-0.385514854	0.014871868	0.010652402	0.020855997
UCHL1	-0.759232729	0.007093085	0.010656366	0.006600329
FCGR2B	-0.40991448	0.079968046	0.010672774	0.028116864
CDKN2A	-0.324243089	0.007066576	0.010687534	7.90E-05
MKS1	-0.708237834	0.005040479	0.010707841	0.00313562
ACP2	-0.485484324	0.094706641	0.010835829	1.37E-06

C7orf69	-0.136201361	0.001376958	0.010919508	0.002651954
TNFSF18	-0.488913314	0.000434723	0.010983474	0.017070324
ZBTB16	-0.211896626	0.409682251	0.011073936	3.93E-05
MKKS	-0.203848916	0.063174659	0.011171792	0.000638084
KCP	-0.425149615	0.019604147	0.011270273	0.020781058
MERTK	-0.178073638	0.124498863	0.011281555	0.040794441
C12orf73	-0.278654391	0.016155356	0.011342453	0.000692538
KRTCAP2	-0.542682207	0.132825357	0.011352527	0.016650805
DOLK	-0.683348923	0.003917714	0.011353722	0.030991695
CBR1	-0.423869314	0.054870129	0.011428378	0.000959123
HTR2A	-0.220024605	0.003012156	0.011441285	0.033809292
ABCF2	-0.295211351	0.008893696	0.011593618	0.021906235
HNRNPA1P50	-0.816295997	0.000875844	0.01163884	0.029142149
LINC02482	-0.353716138	0.006334402	0.011666002	0.002796535
SRGAP3-AS2	-0.537727583	0.000730364	0.011781078	0.034118839
YBX1P1	-0.54068845	0.194947057	0.011878294	1.81E-06
RXYLT1	-0.325979662	0.009408073	0.012112182	0.012384216
SLC29A1	-0.497084179	0.054367711	0.012147929	0.000339238
ANKRD29	-0.289226434	0.029595312	0.01223294	5.65E-05
FCRLA	-0.634288236	0.000697424	0.012257573	0.008082248
SIGLEC15	-0.435137555	0.014452114	0.012527668	0.000139834
MCRIP1	-0.301376818	0.131586583	0.012626919	1.28E-07
SPAG8	-0.385490603	0.004404487	0.012693142	6.56E-06
SLAMF6	-0.29511908	0.006672744	0.012710746	0.000192969
ZCCHC9	-0.344685112	0.021717032	0.01272077	0.000174442

ROR1-AS1	-0.1367911	0.000506506	0.012724218	0.007831162
RECK	-0.170017752	0.022863804	0.012733431	0.008836431
MRAS	-0.368393979	0.085381263	0.012799175	1.75E-06
LINC00963	-0.440971936	0.064833663	0.012867427	0.006071155
ITSN1	-0.180737689	0.012319641	0.012888441	0.030135615
NPC2	-0.235062558	0.384176027	0.013056061	0.017325353
SPOCD1	-0.432117245	0.043146472	0.013076845	0.005384915
PIGW	-0.401965904	0.004152489	0.013343143	0.046011047
SLC17A9	-0.47495538	0.013233915	0.013407549	0.001867557
MTHFD2	-0.277430375	0.082534812	0.01341039	0.000807575
FAR2	-0.078114304	0.13250288	0.013481733	0.000222377
SDHD	-0.129398796	0.07250699	0.01358806	0.005049799
LAMP5	-0.342394024	0.001350927	0.013679556	0.041280881
GSDME	-0.238885908	0.029310063	0.013680844	0.021463563
GABARAP	-0.785642862	0.395619297	0.013702825	0.000547606
CD28	-0.189601257	0.008059421	0.013815295	0.00313562
ISCA1	-0.207836721	0.034121291	0.013815979	0.002984143
THAP11	-0.394068299	0.021098549	0.013869018	0.000265985
LINC02580	-0.301798815	0.001082697	0.013917072	0.043975245
OR2A42	-0.319077192	0.000464987	0.014048052	0.034666092
NLN	-0.203266332	0.035424586	0.014052482	0.006359644
GSTCD	-0.08564151	0.002969368	0.014054409	0.016795334
MFSD1	-0.20102135	0.177277791	0.014081168	2.90E-05
PIGZ	-0.320913509	0.005179322	0.014134454	0.010780964
RECQL4	-0.403023934	0.002475878	0.014243214	0.006617986

SPG21	-0.276383872	0.182533726	0.014315446	1.17E-05
PRSS2	-0.424549723	0.001132103	0.014506809	0.009495991
PTGS1	-0.392390935	0.024624329	0.014555255	0.004735645
VPS4A	-0.221634028	0.021507878	0.014627474	0.004280349
QDPR	-0.17606235	0.032434781	0.014668988	0.019424536
TCTA	-0.518471037	0.013652441	0.014689079	0.020578785
RBM19	-0.236636036	0.031421537	0.014709968	0.019338483
SFRP2	-0.785983538	0.010376646	0.014724052	0.024349607
PVT1	-0.167753685	0.10362594	0.014854985	0.004050353
NT5DC3	-0.267723077	0.013575145	0.014971288	0.022336359
KEL	-0.661382918	0.016624703	0.0153393	0.014908559
NEFL	-0.609997783	0.001358918	0.015384764	0.019068096
LSM2	-0.386925795	0.062693415	0.015402307	0.000625057
ALK	-0.061026054	0.049100681	0.015458976	0.040794441
CISH	-0.628167636	0.016429457	0.015473308	0.043641882
TXNDC15	-0.238631259	0.022845266	0.015504945	0.008380314
LEPROTL1	-0.131520953	0.096313689	0.015577242	0.004590669
HS3ST4	-0.037924469	0.112293639	0.015635561	0.008923153
SMIM27	-0.404547439	0.021161181	0.01568878	0.00891651
MMP8	-0.486462187	0.011248736	0.015705103	0.000631492
UROS	-0.410917513	0.033953598	0.015721442	0.012416159
PAQR5	-0.086063344	0.054343473	0.015777957	4.72E-06
FUCA2	-0.256067175	0.041380711	0.015798596	0.005922834
STX3	-0.211682723	0.502254603	0.015820291	2.54E-06
LYRM4	-0.158518118	0.036469531	0.015832634	0.017657667

MFSD4B	-0.112320814	0.011564215	0.015874705	0.011498693
CD36	-0.143520007	0.270036758	0.015922695	6.21E-06
B3GNT8	-0.371829385	0.005631012	0.015985883	0.006336083
COMTD1	-0.1812975	0.030000061	0.016026095	8.61E-05
TBCAP1	-1.356492526	0.089886238	0.016051594	0.02641287
TIMM8A	-0.399301735	0.014874018	0.01608852	2.31E-05
GOLT1B	-0.180731733	0.032784915	0.016164133	0.004246778
TYROBP	-0.616618772	0.481655776	0.016167687	0.001324425
PARVB	-0.211219697	0.122116035	0.01623242	0.024269887
B9D2	-0.204268924	0.008156097	0.016296757	0.020458332
TPRG1	-0.12023171	0.206467516	0.016352097	0.026788241
PES1	-0.341234978	0.034785846	0.016507599	0.011996535
CDKN2C	-0.441123455	0.008215964	0.016529828	0.006964287
STMP1	-0.158399657	0.166570991	0.016577312	2.26E-06
CD4	-0.314974891	0.083792489	0.016601952	0.022016023
FOXN3-AS1	-0.652933926	0.003608742	0.016658498	0.00372611
FCMR	-0.472018193	0.030182319	0.016696113	4.13E-05
NAV2-AS3	-0.290165743	0.000152733	0.016726958	0.037861118
CHRNA1	-0.362155128	0.001682182	0.016738222	0.001617801
USP2	-0.303892908	0.017514168	0.016747641	0.000235093
CTSF	-0.546788478	0.007293026	0.01691681	0.005432351
TMEM183A	-0.259572847	0.019896895	0.016922142	0.005059736
ECI2	-0.226722703	0.013043918	0.016922577	0.001676202
DDX31	-0.151986193	0.014205598	0.016925765	0.019458483
PPM1M	-0.597881463	0.039115617	0.017015058	8.41E-05

C1orf198	-0.271110187	0.010277089	0.017027572	9.90E-07
HPGDS	-0.138989774	0.021783246	0.017081025	0.03634731
GOLGA7B	-0.402512697	0.008354252	0.017118836	3.36E-05
GPR137	-0.339972411	0.010687875	0.017186956	0.022897833
CIPC	-0.329343112	0.018161412	0.017223373	0.001596085
SLC1A4	-0.401931897	0.030450351	0.017348938	0.046215435
ZNF428	-0.267519184	0.050741765	0.017473218	8.92E-05
AK2	-0.189290157	0.038879051	0.017564534	0.000669978
MTCO1P5	-1.176645572	0.000155539	0.017651367	0.04909033
ASB1	-0.44117064	0.018343738	0.017666439	0.000242459
CORIN	-0.116430039	0.100416998	0.017744777	0.041271564
CDCA3	-0.740783205	0.003561514	0.017832167	4.35E-07
UCHL3	-0.04160592	0.025407561	0.017854009	0.030533908
MAP7D3	-0.265138565	0.01408782	0.017856443	0.024376923
FZD1	-0.387174479	0.007653678	0.017910276	0.019675205
QPCT	-0.273880351	0.066473222	0.017922415	3.47E-05
SPON2	-0.19574811	0.015362916	0.017969451	0.002838734
ATP6V1F	-0.413163692	0.318993646	0.018308625	0.01066403
SLC1A2	-0.262274365	0.028484628	0.018438429	0.005342796
TMEM106C	-0.245281791	0.01291065	0.018492138	3.31E-05
CHTOP	-0.167688553	0.027121645	0.018499701	0.001304833
FAHD2B	-0.353727628	0.005907168	0.018513823	0.016385459
PPT1	-0.423409019	0.188913283	0.018626474	0.000481761
SDCBP2-AS1	-0.20090725	0.007281456	0.018700727	0.00228104
TMEM208	-0.490273992	0.062541328	0.0187687	0.013016973

ATP6V1E2	-0.204046446	0.017188769	0.018806276	0.020317534
CPNE8-AS1	-0.573284239	0.001035419	0.01886661	0.019990111
FGR	-0.557305732	0.212414923	0.018866846	0.000590512
RNF227	-0.518388528	0.004950862	0.018871805	0.015470083
LGALS9	-0.582317244	0.152415359	0.018953647	0.000812119
C15orf40	-0.457879984	0.019811966	0.019283249	0.018015285
LINC00339	-0.305455689	0.015750912	0.019296302	0.00614342
CFAP58-DT	-0.764612537	0.007306773	0.019436284	5.42E-06
CHIT1	-0.299570596	0.28170429	0.019503121	8.15E-06
HSD3BP4	-0.429686072	0.000592977	0.019520768	0.011326774
LIX1L	-0.30146672	0.014508336	0.019558786	0.019846759
TBL2	-0.446398227	0.009435682	0.0197895	0.005730257
C11orf45	-0.647903669	0.008335415	0.019902405	0.037130836
UTP25	-0.418841335	0.009107947	0.02007954	0.00747634
MUC12	-0.621907445	0.01983152	0.020082123	0.001428417
ARHGAP4	-0.2823648	0.080984412	0.02011019	9.51E-06
KRT79	-0.338095889	0.002541902	0.020152023	0.001403075
RTN4R	-0.17126145	0.008061944	0.020254637	0.000251497
MATK	-0.458915391	0.159095192	0.02027631	1.18E-09
TSR2	-0.332889175	0.012890421	0.020329095	0.006262833
TP53I11	-0.353896073	0.028876518	0.02038638	7.06E-06
PPP1R3G	-0.405797714	0.004434164	0.020421141	5.23E-05
TMEM52	-0.604302646	0.001110651	0.020438947	0.034997303
GAR1	-0.316033987	0.020703099	0.020493226	0.002531181
SLAMF8	-0.402806638	0.077233697	0.020501158	5.26E-05

IL2RG	-0.394183644	0.073725937	0.020508248	0.00024838
CSF2RB	-0.345216619	0.025735359	0.020521564	0.043146953
KIF13B	-0.125404066	0.260637322	0.020681919	0.000115839
PIGP	-0.506162472	0.045306246	0.020734727	0.000695414
ITGB5	-0.24762483	0.071671665	0.020895845	1.33E-05
SAMD13	-0.083147883	0.008823067	0.02090717	1.76E-07
LINC02185	-0.553213915	0.010740452	0.020977528	0.000582809
PIP5K1B	-0.055066077	0.051439035	0.021008126	0.020837737
ABCB10	-0.185794688	0.028464404	0.021012444	0.023579118
PSEN2	-0.341746453	0.018974628	0.021070646	6.91E-05
DPAGT1	-0.449831591	0.014435238	0.021132572	0.002737341
IBSP	-0.474131603	0.005200688	0.02118107	0.00011894
STK16	-0.385900844	0.011282039	0.021236872	0.025755549
TRPV2	-0.505894538	0.053598957	0.021248483	0.014957711
LSM10	-0.450604829	0.071669899	0.021508871	0.000598987
PAPLN	-0.233991764	0.026003816	0.021563132	0.000932714
PLK3	-0.502131286	0.065499907	0.021631369	0.001928248
CRYM	-0.207456535	0.042137553	0.021740002	7.43E-06
AKAP1	-0.281042471	0.014470105	0.021747751	0.004053176
COL22A1	-0.172490155	0.031711965	0.021754537	0.000446615
SELENOM	-0.396690917	0.02223685	0.021766451	0.017102286
DNASE2B	-0.540967831	0.053743235	0.021869829	1.55E-06
E2F1	-0.379069784	0.004652247	0.021956714	1.82E-05
MCOLN3	-0.377638852	0.029755439	0.021976246	0.000198155
BST1	-0.25023957	0.03416122	0.021983011	0.013475628

LINC02577	-0.152876421	0.009740223	0.022008309	1.23E-05
LINC00092	-0.734027889	0.001713419	0.022042788	0.037146572
CBWD5	-0.099687649	0.064617936	0.02216039	0.012822114
LILRA6	-0.430379235	0.035009187	0.022164825	0.003355862
MYL9	-0.669664463	0.018272724	0.022344205	0.002737341
CEBPA-DT	-0.802013661	0.00236519	0.022362873	0.032530852
TMEM140	-0.37853816	0.012742638	0.02236962	0.008178453
LINC00504	-0.027051095	0.022147741	0.022451621	0.00100548
NUDT4	-0.268572046	0.04613592	0.022507289	0.017524422
OR6N1	-0.333710724	0.005961311	0.022730452	0.019258028
HLA-DMB	-0.588847816	0.144025729	0.022750122	0.028378156
ATG4A	-0.106593533	0.009850906	0.022828892	0.000466588
UNG	-0.288575437	0.011296612	0.022864079	0.037828328
RRAGD	-0.160778894	0.037391038	0.022869558	0.004844182
EMILIN1	-0.484885971	0.013242917	0.022880879	0.00014775
FCRL6	-0.442319171	0.003284972	0.022892969	0.0028429
LINC02244	-0.648381492	0.053724951	0.022915399	4.14E-06
NUDT16	-0.584145922	0.110121424	0.022931555	1.83E-07
B3GNT2	-0.361756027	0.0554738	0.022967687	0.000119688
ZDHHC3	-0.228991886	0.043736742	0.02304289	0.003477293
PDCD6	-0.241561203	0.042261022	0.023044084	0.048874488
B4GALT1-AS1	-0.424884387	0.003809716	0.023123703	0.003469544
SMYD5	-0.393419981	0.004862066	0.023160681	0.001869546
CYB5R2	-0.378541251	0.005517899	0.023166034	0.004094997
MGME1	-0.233993904	0.017490351	0.023199933	0.000158195

SH3BGRL	-0.172187868	0.234288097	0.023217373	5.45E-05
TBX1	-0.14633161	0.005935414	0.023233532	0.01058529
PCYOX1L	-0.501856118	0.00981264	0.023368241	0.000295009
LY96	-0.274712316	0.124253156	0.023677794	0.033809292
MVB12A	-0.198187188	0.027018299	0.023716854	0.00433547
SV2C	-0.114444424	0.15544616	0.023778813	3.15E-07
RNPS1	-0.41769698	0.038612408	0.023904253	0.004064788
CA3-AS1	-0.242140231	0.003085925	0.023989599	0.008058483
TFCP2L1	-0.382267249	0.070385405	0.024075462	0.0039181
PYCR3	-0.392407866	0.004221776	0.024188553	5.34E-05
HS3ST2	-0.132998248	0.121958656	0.024208953	0.000906294
FUOM	-0.423646292	0.126935229	0.024221524	3.37E-05
PPM1B	-0.078545965	0.119562557	0.024370042	0.017506384
HSPD1P8	-1.136952824	0.00027896	0.024414779	0.008498858
SNHG28	-0.187422601	0.01839387	0.024535053	3.60E-05
CBX2	-0.613550639	0.001858761	0.024542762	0.007473927
CHST11	-0.094577958	0.220966391	0.024545198	0.014416299
ARL10	-0.337166193	0.128065056	0.02465511	0.049732289
PTENP1	-0.492615133	0.014956766	0.024759722	0.016186834
LINC02345	-0.16041428	0.132671516	0.024776384	3.47E-07
MIF4GD	-0.337075241	0.01619744	0.024798049	0.034050405
PNO1	-0.304745244	0.015442274	0.024886009	0.006487297
AP1S2	-0.364697648	0.154934212	0.025006676	5.26E-05
HPS5	-0.250877239	0.026107387	0.025121857	0.0460425
RRP8	-0.483864707	0.009005218	0.025179941	0.0416385

PRKCD	-0.326844862	0.062715118	0.025200833	0.000245596
F5	-0.350511693	0.011451068	0.025273125	0.038903528
PLOD3	-0.487525971	0.033681048	0.025342228	0.007121446
ZNF501	-0.474598663	0.001313432	0.025465835	0.029012562
MYOSLID	-0.116228213	0.000559242	0.025519706	0.036214767
ARHGEF3	-0.066735012	0.165169377	0.025534433	0.000140996
TIE1	-0.479332025	0.002444242	0.025646825	0.033120129
TMEM101	-0.349765117	0.01664077	0.025674299	0.010464403
HDHD5	-0.427970993	0.0366387	0.025724223	0.000763213
GTSE1	-0.327356527	0.006675656	0.025765555	0.003108424
GCG	-0.514042136	0.000746714	0.026028274	0.049204209
ABCB8	-0.422009921	0.013210155	0.026045857	0.000549493
L3MBTL4-AS1	-0.471012989	0.015174259	0.026056685	4.03E-05
LINC00607	-0.14077192	0.057255282	0.026076854	0.003802234
INSYN1	-0.338195066	0.001155789	0.026108487	0.001406243
IGSF6	-0.634889921	0.182657059	0.026230567	0.003676732
PEBP1	-0.265848573	0.111192598	0.026330134	0.043582536
PPM1N	-0.350191454	0.011591202	0.026334404	0.024849641
HPGD	-0.190578875	0.184159134	0.026435202	0.000894484
TPST2	-0.203027832	0.057389421	0.026507727	0.019338483
BHLHE41	-0.581780588	0.074999321	0.026753286	0.042843077
TMEM218	-0.400416263	0.028885815	0.026775247	0.000258852
LINC00997	-0.657084121	0.001003822	0.026809907	0.008053752
BAALC	-0.20155008	0.006665684	0.02683008	0.000208319
GAREM1	-0.047387653	0.099864161	0.027030679	0.020508825

LINC01010	-0.104073756	0.059598415	0.027312872	2.29E-09
PLA2G2D	-0.600921353	0.006274122	0.027338329	0.006732681
ABHD10	-0.254839101	0.024096297	0.027374583	9.09E-06
WDR5B	-0.308900692	0.002262069	0.027409541	0.046078938
DLG5-AS1	-1.164851637	0.000397554	0.027431238	0.030411464
FUNDC2	-0.244263005	0.04900879	0.027443571	0.000362733
TNNI3	-0.551618322	0.001031618	0.027730878	0.010024245
MRPL35	-0.218879592	0.022140701	0.027789312	0.008043751
PARP3	-0.273587398	0.005808647	0.027842242	0.000894484
TMEM74B	-0.358101009	0.005773016	0.027876154	0.002245142
VNN1	-0.268990878	0.026064733	0.028065069	0.024349607
SLC35E3	-0.479104536	0.067108318	0.028124762	0.017325353
C17orf75	-0.243430726	0.005425839	0.028240271	0.043510718
HGH1	-0.54453823	0.009638541	0.028274171	4.14E-06
TEX2	-0.194788049	0.094792191	0.028475176	0.021638191
NOP10	-0.454419256	0.254815135	0.028527022	0.023195292
C9orf78	-0.242756111	0.053946242	0.028614425	5.55E-05
DDO	-0.306522461	0.005655168	0.028632013	0.009705302
ZCRB1	-0.186099717	0.06790416	0.028718575	0.000604812
SPPL2B	-0.237299217	0.009152183	0.028724468	0.013122759
ARL11	-0.481246911	0.02091647	0.028772484	0.006316112
FKBP15	-0.374352048	0.082082807	0.028855033	0.00163986
ZGLP1	-0.480952636	0.013434867	0.02891202	3.93E-05
DMP1	-0.595621092	0.001436285	0.029066621	0.000421464
C1orf43	-0.19503381	0.121654236	0.029067142	0.00011404

URM1	-0.297401873	0.034277364	0.029070538	0.013854136
AP2A2	-0.269887543	0.118059409	0.029084548	0.004487013
G6PC3	-0.430022996	0.023166564	0.029150418	0.002063644
PRADC1	-0.505826047	0.025979787	0.029254381	6.43E-05
AADACL2	-0.163641312	0.001613443	0.029255727	0.016178985
PRXL2C	-0.313437586	0.054905077	0.02938393	0.020721528
KRT18P52	-0.711394392	0.000551734	0.029635757	0.045293159
PPIAP16	-0.860523956	0.011002932	0.02972111	0.01589525
ATP7B	-0.322272812	0.031185988	0.029737486	0.000744768
CHKB-DT	-0.513165653	0.007241113	0.029761524	0.000123282
TACO1	-0.429122258	0.009351537	0.029909101	0.040072171
CRIP1	-0.501663433	1.198326968	0.029962874	1.63E-09
PDE4DIP	-0.166497252	0.135263177	0.029988934	0.042094911
FXYD2	-0.33583735	0.027002874	0.030209621	0.020781592
CENPM	-0.286720148	0.010995732	0.030571881	1.47E-05
DNAJC19	-0.337203909	0.024822273	0.030597473	0.036155334
DHRS11	-0.633057313	0.004835952	0.030656426	0.019584632
POGLUT1	-0.340059145	0.015895512	0.0306815	0.027826546
OTULINL	-0.365115265	0.06747895	0.030712016	0.000675316
NABP2	-0.331069263	0.017973762	0.030725473	0.000129543
SDSL	-0.249921408	0.050606253	0.030732997	0.004953371
COQ4	-0.268427871	0.029482347	0.030773921	0.000362733
DISC1FP1	-0.024297266	0.088098313	0.030921731	0.038450219
CD207	-0.662995363	0.000697831	0.031012693	0.049560078
STOML1	-0.554459516	0.005567298	0.031073213	0.000258239

LINC00467	-0.26331114	0.027633819	0.031138878	0.00047082
TBC1D9	-0.134360488	0.227311646	0.031245688	0.000725978
OTOAP1	-0.217601856	0.123464576	0.031268429	0.000173073
LST1	-0.884413606	0.286405689	0.031369592	0.005059736
IL36RN	-0.685198952	0.000428138	0.031386459	0.010514128
BTBD6	-0.356766389	0.009410327	0.031517357	0.01514397
C1orf54	-0.46054542	0.060447419	0.031520958	0.000422245
POC1A	-0.141705828	0.004345964	0.031579267	0.034567817
CA11	-0.403154064	0.009609836	0.031869252	0.008319674
SMIM12	-0.160004897	0.032087021	0.031936234	0.028754602
CTSG	-0.991545129	0.002156425	0.032022484	0.01873293
SMAP2	-0.145626454	0.203607221	0.032107258	0.024128439
UNC50	-0.188972421	0.023229417	0.032117805	0.044791781
CNIH3	-0.056441125	0.026716491	0.032130048	0.018027203
PKD2L1	-0.266506739	0.009592708	0.032190251	0.010524488
DERL1	-0.213331351	0.025308837	0.032464142	0.016308565
ARFRP1	-0.518513196	0.018993748	0.032626968	5.65E-05
COPRS	-0.470902814	0.063806017	0.032698424	0.000131323
MND1	-0.07245487	0.004926761	0.03274204	0.037086849
TMEM128	-0.213953717	0.024566126	0.032831632	0.003020478
ESCO2	-0.266361869	0.003601861	0.032886313	0.03487327
TNFSF15	-0.412785835	0.019215239	0.032989748	0.020855997
FAM204A	-0.253764205	0.044366277	0.033209294	0.001469992
XYLB	-0.262827909	0.009407204	0.03335622	0.023527892
RBM28	-0.39255499	0.0239173	0.033414794	0.008929995

LINC00920	-0.591260546	0.00138335	0.033509498	0.024348213
SLC28A3	-0.162805704	0.080193986	0.033514145	6.15E-08
GPNMB	-0.479705367	0.481806332	0.03351724	6.44E-05
YARS2	-0.165288776	0.011647688	0.033701227	0.026783319
TRMT61A	-0.374924266	0.008954371	0.033881015	0.010580578
ZNF436-AS1	-0.391967882	0.001311978	0.033909629	0.005987093
SEPHS2	-0.488784397	0.03909824	0.034003872	0.009045385
CDT1	-0.516170481	0.015795065	0.034389635	2.85E-05
RAB6D	-0.802727835	0.023662684	0.034392241	0.005042307
PLEKHA1	-0.170572835	0.021934741	0.034403471	0.017127889
FKBP1C	-0.727232568	0.22312787	0.034430837	8.87E-06
CREB5	-0.077442838	0.108374407	0.034433511	0.046453006
ERLIN2	-0.282705392	0.01440193	0.034707281	0.009014208
SMIM10L1	-0.399612367	0.015317192	0.034749534	0.043698011
DAB2	-0.419295271	0.013141152	0.034841268	0.001414255
TDGF1	-0.559350911	0.000476454	0.03486874	0.039644694
SLC25A48	-0.096051124	0.001392653	0.034966326	0.01350491
PLA2G7	-0.364097039	0.142533632	0.035302319	0.000579752
DTX3	-0.459059248	0.000846907	0.03532713	0.039382601
AMDHD2	-0.330495261	0.024925137	0.03549907	0.0324415
LINC00667	-0.220343363	0.021411791	0.035641944	0.028796526
BCAP29	-0.08087764	0.037929182	0.035872342	0.000844074
EIF2B2	-0.274824147	0.01285446	0.036192001	0.002588601
ARHGEF5	-0.300128588	0.003346792	0.036255899	0.011649679
GM2A	-0.432389286	0.141680618	0.036505521	0.000422619

USP10	-0.162285464	0.046879184	0.036690198	0.018285705
LINC02166	-0.30332134	0.001585633	0.036848328	0.000779652
EPPIN-WFDC6	-0.186927589	0.001221601	0.036855991	0.002659431
NFYB	-0.151928203	0.010593079	0.03699651	0.01137637
UCK2	-0.185911899	0.040454519	0.037035037	0.001856351
C4orf46	-0.370532785	0.005028477	0.037067728	0.03193608
MTLN	-0.179186557	0.083935984	0.037072927	0.000194328
APOBEC4	-0.289799476	0.000779246	0.03707595	0.04913075
ATP6V0E1	-0.212024332	0.214475269	0.03714278	0.00496148
ID2-AS1	-0.62110634	0.00999441	0.03724746	1.65E-06
SPA17	-0.308428006	0.00415214	0.037518729	0.000538801
SRPRB	-0.155345468	0.023115035	0.037653915	0.023399402
HCFC1R1	-0.367132878	0.105830898	0.037688082	1.26E-06
EXOSC5	-0.324473311	0.023877579	0.037741935	0.001533684
MGST3	-0.270022657	0.18261236	0.037860306	0.03922262
DTWD2	-0.075095889	0.226410462	0.0378613	0.005400328
CDPF1	-0.418605237	0.004522422	0.037930516	0.012827938
TTC39C-AS1	-0.218911885	0.000584099	0.037958444	0.028851934
MEGF6	-0.115711279	0.005485992	0.037961599	0.015900111
C18orf32	-0.450123849	0.182972205	0.037970363	1.03E-05
HSD3B7	-0.536947339	0.104233458	0.03802383	1.93E-08
PTGDS	-0.374584857	0.014517433	0.038100823	0.035193715
TSC22D3	-0.072157871	0.440489854	0.038111716	2.44E-06
ERLIN1	-0.212241535	0.042528519	0.038449752	1.36E-05
LILRB5	-0.499834347	0.029800066	0.038483485	0.037804253

FABP5P11	-1.319449864	0.002184588	0.038624107	0.023093489
TPP1	-0.406848005	0.19061992	0.038832573	4.51E-05
LIN7B	-0.250364232	0.010342234	0.038867086	0.002651954
BRI3	-0.274068779	0.240107214	0.038884105	0.00187403
TSPAN2	-0.181067762	0.003799674	0.038935955	0.007524736
ZBTB7C	-0.058395788	0.023968425	0.038950548	0.033795777
ARPC4	-0.288152173	0.126085132	0.038966007	0.000727609
TDRD6	-0.374576536	0.044708166	0.038983989	0.000455718
YIF1B	-0.279705986	0.035414104	0.039181462	0.010358248
MARCO	-0.342858257	0.318434694	0.03953111	0.020040625
NUBP2	-0.277327339	0.029021099	0.039617801	0.003232646
LINC01282	-0.220829138	0.001751777	0.039736028	0.000246178
NSFL1C	-0.20083756	0.025404663	0.03979331	0.028133943
SPATA12	-0.376707734	0.004954532	0.039968278	0.00024871
CFL1P1	-0.173355057	0.01141188	0.040182319	1.98E-05
TRDMT1	-0.283787327	0.016654523	0.040407782	0.004645968
OR2C3	-0.286618482	0.001765119	0.040438904	0.028407525
DEPP1	-0.490308463	0.009715089	0.040530155	0.030880694
ATP6V0D2	-0.075783939	0.098368982	0.040583462	6.18E-06
FAM72B	-0.181974854	0.005752903	0.04070729	0.012723744
LINC01614	-0.784617134	0.001021406	0.040920075	0.016688981
GPN3	-0.337156164	0.023190076	0.040935435	0.041562723
CCL14	-0.499030765	0.002666484	0.041006919	0.008419694
PTGES2	-0.384216868	0.034529702	0.041167982	2.32E-05
SLC9B2	-0.193799575	0.014887103	0.041346148	0.008202787

AK1	-0.292775363	0.01217271	0.041418208	0.002719928
ZNF514	-0.268295164	0.00352513	0.041558774	0.027194539
IQGAP2	-0.086485093	0.200360773	0.041629634	0.028294119
FCRLB	-0.63150224	0.008731186	0.041720255	0.001157726
MAPKAPK5-AS1	-0.41232526	0.034764547	0.042084358	0.000936637
ESD	-0.260985892	0.074339723	0.04215311	0.045766612
TAF10	-0.341158101	0.023767672	0.042239545	0.024684567
FAHD1	-0.233355932	0.017590378	0.042272766	0.002300605
PRKACB	-0.07036351	0.08886996	0.042297773	2.98E-09
MRPL9	-0.288423674	0.007500173	0.042383615	0.012354721
TMEM178A	-0.065812746	0.001922497	0.042476379	0.035215433
LINC02357	-0.230696399	0.009914625	0.042528384	4.79E-06
SLC52A2	-0.18237819	0.025004367	0.042735623	0.008929995
SLA	-0.229463488	0.124706826	0.042833593	0.011279883
LTBP3	-0.426623088	0.021465168	0.042888087	0.000106952
HAGHL	-0.178357467	0.031784127	0.043511855	0.000637175
B3GAT3	-0.352416954	0.027716558	0.043619357	0.005919396
GPATCH4	-0.50882798	0.029501241	0.043651874	0.000794912
COX16	-0.028122312	0.101568029	0.043681481	0.003182231
OCSTAMP	-0.391319641	0.001287366	0.043692788	0.024112218
PSD3	-0.050768544	0.253462506	0.044194952	7.51E-06
CSF1	-0.537897691	0.066724493	0.044329968	1.93E-08
BATF3	-0.452200993	0.030497734	0.044442586	0.000331585
AGPAT5	-0.172273904	0.013162558	0.044584925	0.013564605

GAPLINC	-0.236560143	0.032799072	0.044622082	0.000273857
AGTRAP	-0.187655888	0.052388667	0.044634106	0.006043431
CCL23	-0.328910891	0.049065069	0.04485927	4.69E-05
UBXN8	-0.269886505	0.014626073	0.044936676	0.022648018
OR52K3P	-0.093802788	0.003485037	0.045012823	0.045766612
CTHRC1	-0.587366106	0.004192914	0.045241317	0.02568025
CDCA7	-0.373359516	0.002669205	0.045270225	0.031587127
GALNS	-0.27451655	0.027340391	0.0453449	0.005283784
TRAM2-AS1	-0.195876486	0.002418255	0.045394783	0.006297575
CRYM-AS1	-0.133223056	0.039451674	0.045473423	0.000482546
RNASEH1-AS1	-0.504638231	0.007214099	0.045563817	5.96E-05
DECR1	-0.106993597	0.076257808	0.045674238	0.030460993
WIZ	-0.347163455	0.006345667	0.045678929	0.003108424
MTX1P1	-0.59019202	0.048887109	0.045727095	0.003609323
TRABD2A	-0.165733473	0.027699912	0.045772762	0.025823059
CUTA	-0.271542542	0.099012464	0.045945861	0.015865137
LINC00881	-0.234093478	0.001021834	0.04610301	0.02526594
MCM3	-0.359962464	0.016060886	0.046131479	1.86E-05
LXN	-0.1662499	0.017612353	0.046344679	2.30E-07
RNF13	-0.094904533	0.137388015	0.046562608	0.001854974
DGUOK	-0.109955559	0.050715749	0.046861257	0.032421095
TLCD1	-0.532546301	0.002490347	0.046992829	5.55E-05
PART1	-0.342887515	0.007826196	0.047026615	0.004676087
GAL	-0.457623966	0.005076461	0.047085853	0.024315978
C2CD2	-0.151878192	0.025828083	0.047110124	0.019284105

GLIPR1	-0.381010434	0.046148247	0.047320274	0.000366637
IPO4	-0.205141901	0.004426826	0.047402335	0.013537712
MCRIP2	-0.273307688	0.070797528	0.0474032	0.018990634
DCTN3	-0.243986775	0.055429443	0.047782914	0.005441855
CLEC12B	-0.568905206	0.005234763	0.047873871	0.010447517
C9	-0.056157749	0.388915794	0.048488981	5.72E-08
TMEM70	-0.350636116	0.093739328	0.048746375	4.58E-05
ZNF295-AS1	-0.293853692	0.003909244	0.048817209	0.019133867
COL4A2-AS2	-0.480041494	0.033916706	0.049266558	0.000406977
B4GALT7	-0.394121436	0.019513721	0.049289847	0.006918857
TMEM107	-0.53627546	0.0325955	0.049306234	0.026888467
LYRM2	-0.098150313	0.022817938	0.049396516	0.030900666
COL13A1	-0.206809259	0.005444208	0.049448399	0.027855824
C9orf40	-0.190377563	0.003716577	0.049708559	0.00689747
BMS1P15	-0.24170264	0.010831855	0.049845121	0.028914214
ALG3	-0.501499462	0.033323532	0.04988911	0.001978199
C20orf197	-0.33588059	0.004402492	0.049908133	0.02767335
NOM1	-0.295904841	0.019531104	0.050028514	0.001779047
TMEM75	-0.55281444	0.000334217	0.050107963	0.021358978
GRM7-AS1	-0.122658911	0.001729851	0.050202595	4.90E-06
RGS10	-0.248349986	0.153448179	0.050337305	0.00086263
ALKBH2	-0.511848388	0.010094303	0.050359998	0.0007883
CCDC115	-0.292113771	0.056506028	0.05044126	0.000125596
LSM6	-0.234010026	0.090106312	0.050473317	0.00970534
PRMT6	-0.308611457	0.008702093	0.050516207	3.34E-06

PDCD4-AS1	-0.339268381	0.004485174	0.050625252	0.000833978
USP11	-0.421477276	0.018897197	0.050634649	0.0278017
ZNF330	-0.278856288	0.037607053	0.050861948	0.002279495
SMUG1	-0.216066752	0.023380766	0.050994826	0.001773856
PFN1	-0.322282457	0.305124404	0.051124896	0.034565904
METTL1	-0.628294914	0.00594382	0.051187271	0.000220059
GTF2H3	-0.104254014	0.023214759	0.051302325	7.27E-06
MAPKAPK5	-0.182896092	0.02011665	0.051348376	0.008064718
CCDC86	-0.368605343	0.001372695	0.051412148	0.020220095
COX20	-0.343348761	0.027473906	0.051511257	0.007731782
RRS1	-0.479187797	0.02260727	0.051792613	2.41E-05
ORMDL2	-0.299902546	0.046175159	0.051806923	0.000356533
HENMT1	-0.347101519	0.009495677	0.052396171	0.013997666
ISCA2	-0.458366013	0.02986004	0.052621386	0.004138411
ITLN1	-0.45993741	0.005914675	0.052894477	0.03987732
HSD11B1	-0.252629252	0.011991082	0.053094542	0.001844927
UBE2D4	-0.212086892	0.025394541	0.053301254	0.012108253
A2M	-0.423971751	0.149095727	0.053336691	8.96E-08
ADGRE3	-0.146892993	0.099163283	0.053356617	0.001087009
CCDC22	-0.192271081	0.01045503	0.053409853	0.022166878
MESD	-0.245871532	0.048293928	0.05348887	0.000343352
RNF14	-0.168632665	0.013930042	0.053856398	0.030219839
RAC2	-0.400600894	0.172663211	0.054176424	1.94E-08
C1D	-0.107089666	0.040616118	0.054458359	0.003443511
KRT80	-0.362587424	0.000528346	0.054512326	0.00927389

ODF3L1	-0.382393159	0.006367841	0.054626607	0.000130727
CD300C	-0.534084838	0.035917256	0.054818063	0.012053117
TMEM219	-0.150482685	0.124405431	0.054914401	0.008662181
ASAP1	-0.109189404	0.269588382	0.05496754	0.003467907
OGFRL1	-0.139969598	0.085109577	0.055008595	1.30E-05
TMEM109	-0.48202342	0.046384547	0.055121644	8.55E-08
ADTRP	-0.183422898	0.044196517	0.055398478	0.002523176
TFPT	-0.348374532	0.041318231	0.055435379	0.009806721
ZDHHC24	-0.28159234	0.034558892	0.055544177	0.002215257
CCDC85B	-0.487910081	0.141904112	0.05560298	2.34E-06
ANKRD66	-0.305857413	0.002082339	0.055884538	1.33E-05
RRP1	-0.364432813	0.020682271	0.056047383	0.004439011
CDK5R1	-0.49488489	0.00144919	0.056319353	0.007120988
DCTPP1	-0.26087494	0.040109074	0.056327819	2.31E-05
AP1AR	-0.217454159	0.01859074	0.056493947	0.002308438
SLC25A11	-0.263656053	0.054551758	0.056644171	0.001216819
GLIPR2	-0.337661459	0.199330442	0.056676855	0.003146983
CD52	-0.737963366	0.674533211	0.056696353	7.27E-06
OGFOD3	-0.243274768	0.024017736	0.056929634	0.000927117
SNX12	-0.206134713	0.015127304	0.057359825	0.021332781
CD1B	-0.771276147	0.003518144	0.057723242	0.036092069
ME3	-0.082662719	0.01652267	0.0583339	0.020526474
ARL14EP	-0.157881989	0.023279123	0.058461845	0.012011136
TMEM19	-0.287666386	0.012692652	0.058707197	0.035667136
ARHGEF16	-0.151955859	0.000710589	0.059012249	0.032913681

KIAA0930	-0.449996324	0.091242064	0.059377333	0.000179594
PVALB	-0.257102056	0.007899406	0.059691952	0.000980831
EXOSC2	-0.290117016	0.008032835	0.059783053	0.012053117
SORT1	-0.157874034	0.163765636	0.059873299	0.000368437
SETD7	-0.177395371	0.035266405	0.059885858	0.00461917
CKAP2L	-0.241760206	0.003457107	0.060240401	0.017965321
SPRYD7	-0.200739233	0.024174011	0.060375438	4.90E-07
KIF20A	-0.389648638	0.00254593	0.060552078	2.56E-06
NDUFB9	-0.276810678	0.18138275	0.060708569	0.002187849
KCNA10	-0.723554716	0.000480729	0.061464134	0.008079963
ZG16B	-0.378453059	0.006987964	0.061592245	0.011341397
ACADM	-0.134533401	0.027539628	0.061638407	0.033395166
LIPA	-0.311074221	0.445734182	0.061696386	1.94E-08
C16orf86	-0.371022372	0.004648505	0.062932769	0.008899456
REXO2	-0.12784173	0.051640246	0.062989759	4.14E-05
FAM217B	-0.27003581	0.007763531	0.063029674	0.040342775
ARHGAP11A	-0.219517315	0.007150967	0.063689802	0.001828162
FXYD1	-0.184349307	0.006298567	0.063817127	5.92E-05
KLK1	-0.569424269	0.008034653	0.063974821	4.38E-05
FABP5P1	-0.891512222	0.02897254	0.064155743	0.001117323
NUDT19	-0.194146953	0.025282891	0.064330126	0.008679379
ERCC6L	-0.10095013	0.000824333	0.064668437	0.015843487
CHCHD6	-0.042258214	0.029170267	0.065149232	0.008079963
STX2	-0.185068211	0.044660663	0.065262913	0.000387947
ZNF561-AS1	-0.238465132	0.006574386	0.065353646	0.002010772

KLK4	-0.578628138	0.003489566	0.065407627	5.48E-06
ATG3	-0.271878475	0.056395681	0.065562862	0.002242712
FABP3	-0.445560473	0.150685883	0.065709909	5.92E-09
FAM72A	-0.227153309	0.014710711	0.067248529	6.44E-05
AZI2	-0.097165848	0.092539306	0.067556456	0.000783092
CKLF	-0.076001509	0.091585752	0.067649952	0.013171995
SLC18B1	-0.115110108	0.037066747	0.067739514	0.00058423
DNLZ	-0.359126584	0.01627117	0.067925291	0.017181315
SLC2A5	-0.216460961	0.018301494	0.068115085	0.038527539
VANGL1	-0.205881175	0.004884131	0.068508491	0.006778105
B9D1	-0.182215466	0.009159384	0.06854005	0.00120881
FABP5P2	-0.924211799	0.130396509	0.068604242	1.25E-07
FRAT1	-0.55890192	0.013057723	0.068607405	0.020240629
SCARB2	-0.239531629	0.081563981	0.068701733	0.002464522
KIF23	-0.263231687	0.006476177	0.068804864	0.000429599
MIEF1	-0.332452096	0.010932779	0.069036971	0.01805748
SYCP1	-0.05695236	0.013516191	0.069251054	0.00320391
NXPH3	-0.574317897	0.000321883	0.070140404	0.036155334
GLOD4	-0.219969827	0.043460656	0.070154903	0.001868149
BYSL	-0.314622136	0.005208846	0.070282875	0.016317127
AMACR	-0.199565491	0.004936711	0.070712156	0.004628879
OR7E37P	-0.426806898	0.006261626	0.070721259	0.012108253
S100A4	-0.399674877	0.443605749	0.070840067	0.012493375
FAM72D	-0.063761433	0.011326369	0.070999137	1.37E-06
SLC39A11	-0.046681137	0.217211139	0.071470828	0.013997666

MMP9	-0.63578063	0.143795033	0.071605302	0.001014606
SUMO2P1	-0.909863785	0.023917027	0.07180941	0.032710612
ZNF462	-0.158874812	0.009947519	0.071904977	0.001121003
MTX1	-0.243412686	0.014456489	0.071906028	0.01351409
CAPG	-0.312446779	0.286961366	0.071950511	0.010737521
SCP2	-0.082612277	0.148751268	0.072219912	0.000186224
KIF1B	-0.193793236	0.175414904	0.072332288	0.006756917
LINC00520	-0.400931506	0.015952987	0.072448605	8.89E-05
HDDC2	-0.10535951	0.06400875	0.072565171	0.010928738
PRR13	-0.317433309	0.202792269	0.072582369	3.16E-05
LINC01271	-0.283493086	0.014555795	0.072693316	0.016673794
RNASEH2C	-0.348670734	0.054982957	0.072703876	0.016504253
VIM	-0.516627078	0.770496618	0.072852135	7.71E-08
DPH2	-0.436508302	0.004957139	0.072879341	0.009689603
DYRK2	-0.319787082	0.01045989	0.072948332	0.045058817
E2F8	-0.33199815	0.001860018	0.073159489	4.01E-05
LPCAT2	-0.074859029	0.06790251	0.073195209	0.019720243
MRPS14	-0.151532228	0.035457138	0.073369058	0.03009733
POLR3D	-0.375240758	0.0082926	0.073463987	0.002026998
EIPR1	-0.085543293	0.028412303	0.073627561	0.01429668
LSM8	-0.261557234	0.054786667	0.073920857	0.018761681
IFI16	-0.246847625	0.096367121	0.073996018	0.011939181
LINC01492	-0.06354613	0.027993455	0.074144906	0.020526369
RBP4	-0.35527364	0.065492559	0.074393231	0.000106936
GUSB	-0.223775304	0.119086129	0.074410777	3.90E-06

DIMT1	-0.093017944	0.023995399	0.074519367	0.000783092
HACD4	-0.192185291	0.054926335	0.074647343	0.01266682
CHCHD5	-0.519120146	0.068412338	0.074943676	0.004152905
DOHH	-0.290541381	0.011662407	0.075308816	0.004838645
TCF19	-0.547134859	0.006911854	0.075470784	3.60E-07
CAMK1D	-0.0325755	0.26719096	0.07569518	0.044216523
ACOT7	-0.12838497	0.133487918	0.076015905	0.000577728
TOMM40	-0.302691632	0.07370928	0.076037809	0.006654719
REEP6	-0.247277944	0.003973291	0.076124578	5.84E-05
CTSZ	-0.448679861	0.321891452	0.076471446	0.000193427
OR7E66P	-0.926341316	0.007592373	0.076654956	8.75E-05
GATD3A	-0.021751447	0.03602157	0.076834235	0.000931461
NUDT2	-0.21828964	0.018112279	0.076980458	0.031418753
PHETA2	-0.298987446	0.002456561	0.07736074	0.020871889
ANKRD28	-0.089310132	0.558777836	0.077678558	2.33E-06
BLOC1S2	-0.302363592	0.111204516	0.078215429	1.19E-05
MAP1LC3B	-0.199256143	0.079554607	0.07830151	0.038814386
DSCC1	-0.154641144	0.003003712	0.078580592	0.004843516
CYP27B1	-0.428976985	0.031711359	0.078613396	2.00E-07
LDHD	-0.491915842	0.006183118	0.079017459	0.042866087
UTP15	-0.254540853	0.005422929	0.079088264	0.01621622
BBS10	-0.251453369	0.006215323	0.07917462	4.13E-05
NMBR	-0.076804513	0.003990543	0.079182964	0.009758689
TMEM179B	-0.302475171	0.014053746	0.079467486	0.034144346
TXNL4A	-0.188463399	0.054870227	0.079634449	0.006584247

MYOZ1	-0.432040687	0.003148655	0.079843468	0.004115793
TMX4	-0.119364077	0.071315927	0.080103592	3.45E-05
ZNF706	-0.210264353	0.252257154	0.080625083	5.93E-05
RAD51AP1	-0.248878751	0.01385582	0.08085015	0.000562186
PROS1	-0.133587252	0.017133952	0.081048674	0.017961717
ENHO	-0.32787795	0.005494234	0.081117724	0.009777148
LINC02603	-0.064982748	0.003502858	0.081177089	0.000967768
VAT1	-0.501140134	0.129484922	0.081500774	0.000202721
FDX1	-0.248440625	0.309841195	0.081746974	1.76E-07
RIOX2	-0.203922202	0.008337684	0.081748416	0.00927389
THEM6	-0.240742681	0.007510137	0.081825776	0.032343005
WFDC6	-0.417141526	0.000639853	0.081939703	0.008133841
FAM120AOS	-0.302506174	0.019322638	0.082097641	0.010603032
GRWD1	-0.374051544	0.014438778	0.082551669	0.002731325
RTL8B	-0.478985784	0.010128102	0.082606867	5.04E-05
NAGA	-0.273746193	0.05931942	0.083248207	5.04E-05
NDUFS7	-0.212698273	0.027538631	0.083461203	0.034981431
ATPAF1	-0.175761564	0.017078622	0.083608372	0.000427462
TIMP2	-0.210618	0.098720378	0.083673509	0.041620283
FOLR2	-0.396221803	0.069337241	0.083759944	0.01439567
RABL2B	-0.197605406	0.012680705	0.084623819	0.008842896
PUS10	-0.081223489	0.081934043	0.084864093	0.014620748
TFAM	-0.25945615	0.017798766	0.084980609	0.008222124
CLPP	-0.301794171	0.026824516	0.085024166	0.01205479
GYG1	-0.172478187	0.037132107	0.085236982	0.002199262

DTD1	-0.058977485	0.024634263	0.085537435	0.021606787
LINC01765	-0.370126873	0.001184066	0.086071346	0.002588601
ARL2	-0.278209059	0.075145275	0.08623311	0.000489314
LINC02285	-0.68760777	0.006103417	0.086244862	0.036944348
NOP16	-0.341254088	0.029408525	0.086394901	7.58E-05
KLF6	-0.450585003	0.334737388	0.086585969	0.010464403
UBE2A	-0.19417045	0.041706625	0.086664815	0.021012947
CHAC2	-0.438406996	0.002964288	0.087182224	0.013927177
WRNIP1	-0.29402593	0.01347193	0.087411157	0.025778908
MRPL50	-0.158139597	0.034992322	0.087791202	0.002396772
JAGN1	-0.301190395	0.018872592	0.088431622	0.038330547
DHRS4	-0.19688067	0.045289065	0.088960847	0.007016829
DYRK3	-0.221402539	0.014236215	0.089193396	0.013405767
AP1S1	-0.158534066	0.029916426	0.089709249	0.017102286
AADACP1	-0.134125736	0.000880195	0.089923843	0.017383616
KRT42P	-0.462189971	0.001138026	0.090362258	0.003521387
NAGS	-0.34403766	0.00167128	0.090771275	0.006518002
SMIM29	-0.430685132	0.039914409	0.090862543	0.000652242
TPRN	-0.200019977	0.023693625	0.091327895	0.000515713
CYP4V2	-0.246513917	0.016991146	0.091471406	0.029635547
EMG1	-0.178503344	0.039978141	0.091543468	0.000694796
MMAB	-0.215868409	0.018325077	0.09182462	0.006144256
PEMT	-0.055950624	0.020333673	0.092909585	0.003136465
ARPC5	-0.226761209	0.243170059	0.092916066	0.00026263
MREG	-0.090070644	0.083418261	0.092964834	1.70E-05

GAS2L2	-0.215098062	0.001015746	0.09338129	0.021336695
CISD1	-0.193940694	0.029057179	0.093692753	0.001531541
SAAL1	-0.138543768	0.005725358	0.093841653	0.041622747
ENY2	-0.265515442	0.109920321	0.093856389	0.009487564
CMTM3	-0.368922617	0.03723034	0.094162305	0.014304052
CCDC90B	-0.186939757	0.040879967	0.094228733	0.000308925
FUNDC1	-0.138060702	0.011314644	0.09453163	0.020108813
ACTR10	-0.135666811	0.034400887	0.094623046	0.003050259
LNPK	-0.097039607	0.030931575	0.095263473	0.008318241
NDUFA5	-0.27846266	0.045136006	0.095413503	0.03658617
DDX39BP2	-0.812828068	0.000982253	0.096331567	7.27E-06
GMFG	-0.307788947	0.168148164	0.096468542	0.025329868
MYO1E	-0.124522263	0.239936951	0.097231184	0.000474312
MRPL16	-0.304279618	0.023825063	0.097258046	0.005117164
CENPF	-0.2592751	0.007878329	0.097450581	0.039046193
IDH1-AS1	-0.60128036	0.002050395	0.097548625	0.042992346
PIGF	-0.114592392	0.049751404	0.097644502	0.028553571
FAM86DP	-0.318367774	0.013242896	0.098211939	0.003741671
ITPA	-0.177027819	0.06326295	0.098302804	0.000544751
FAM111B	-0.138113363	0.003942721	0.098564413	0.002555353
EMC8	-0.132411912	0.019825386	0.098771841	0.020155684
LAMP1	-0.431301297	0.154074289	0.098913794	0.018883545
MAPKAPK3	-0.294722209	0.036609591	0.099264131	0.044332148
NDP	-0.180632841	0.005410857	0.099607267	8.04E-06
DCLRE1A	-0.236154236	0.002303068	0.099680853	0.039766569

MCUR1	-0.204171239	0.030490147	0.099801024	0.010686231
NIP7	-0.258830407	0.018795087	0.100834223	3.39E-05
ERI3	-0.058037917	0.028068992	0.101701862	0.013837741
MTIF3	-0.181619887	0.048032025	0.101787231	0.027070923
KDSR	-0.165115094	0.028983395	0.102055665	0.038297119
C20orf27	-0.22200326	0.07044469	0.102101078	0.008464391
ITGAM	-0.162395054	0.161346042	0.102280853	1.42E-06
WEE1	-0.2911145	0.022224408	0.1023945	5.67E-07
UOX	-0.149792246	0.002767257	0.10252776	1.48E-05
DPM2	-0.417087235	0.047068054	0.102761714	5.81E-06
FAM98C	-0.353772944	0.007667621	0.102867337	0.006876027
APOC1P1	-0.446382129	0.091561323	0.102994374	0.006592595
PLBD1	-0.134618658	0.129618186	0.103085934	0.00082618
SEM1	-0.068869029	0.095944941	0.10318772	0.046354874
TERF2IP	-0.046675946	0.030729189	0.104185326	0.020935273
RHEB	-0.179096176	0.123260617	0.104295044	0.005609006
PPP1R11	-0.189153899	0.056839874	0.104310265	4.53E-05
UBE2T	-0.34277111	0.008909496	0.104328122	0.000593677
BOLA3	-0.334044584	0.078762893	0.104615655	1.65E-09
HK3	-0.409525395	0.03485647	0.104815179	0.008297889
OXLD1	-0.183877438	0.023824655	0.105251905	0.00233953
COQ9	-0.14432057	0.01122663	0.105280329	0.022701852
ZNF598	-0.416363948	0.003314438	0.105892613	0.000878212
MRPL34	-0.262082488	0.052327594	0.10610273	0.03885912
APOC4-APOC2	-0.113400965	0.179061312	0.106202837	0.009806721

TAB2-AS1	-0.196958414	0.000543183	0.10645575	0.004587386
ZNF812P	-0.219377518	0.00285049	0.106592544	0.006330729
DAGLB	-0.135300448	0.096530952	0.106665771	0.004183181
VEGFB	-0.459093177	0.02773682	0.106873296	0.021463563
TINAGL1	-0.262923676	0.002802828	0.107136805	0.003182231
IMP4	-0.219695433	0.066338754	0.107443016	1.75E-05
TIGD2	-0.281214159	0.002868028	0.107461298	0.0369359
KIF22	-0.289336195	0.018145934	0.107577746	0.041997006
BOP1	-0.163816183	0.016677394	0.107693467	0.010705818
ZNHIT1	-0.171850869	0.145592029	0.108078894	7.70E-05
MLX	-0.264739535	0.035949033	0.108340348	1.36E-05
BET1	-0.111375333	0.021877177	0.108547164	0.011781953
NIPSNAP2	-0.066960309	0.042319145	0.108857123	0.010799029
MRPS30	-0.221989878	0.015769631	0.108910308	0.041254423
SHARPIN	-0.172074698	0.043755827	0.109475386	0.000325612
NUTF2	-0.203624564	0.105652681	0.109507363	0.000229363
CYB561	-0.201744296	0.007310826	0.110505379	0.00233953
POLE4	-0.290580558	0.141628077	0.110633546	2.74E-05
CD276	-0.318101689	0.116391984	0.111370488	1.22E-14
SLC39A4	-0.223572116	0.023733289	0.111915078	0.009464901
RNF187	-0.284660499	0.025384124	0.112301164	0.041404247
TMEM126B	-0.331841229	0.052594779	0.112805647	0.000231401
DHRS4L2	-0.120610722	0.066936267	0.112831475	0.000202066
SENCR	-0.376295192	0.005194157	0.112993011	0.001405492
DNAJC4	-0.23063892	0.029984683	0.113163234	0.001596085

NUDT1	-0.284315998	0.042264438	0.113222613	0.002537798
ALG14	-0.129059096	0.019907181	0.113950538	0.032098395
HSPB7	-0.413791856	0.00070332	0.114106103	0.00840423
AK6	-0.189582113	0.105310291	0.114428536	0.00087837
CXCR3	-0.380833636	0.000553396	0.114486109	0.041710907
TMA16	-0.144455106	0.03567917	0.114525747	0.006351259
MPLKIP	-0.251701387	0.04999206	0.114629675	0.002139992
ABALON	-0.321405633	0.005117225	0.114961049	0.041035028
ACOT11	-0.12479823	0.037104856	0.11499363	0.007121446
KLF4	-0.307150277	0.221351987	0.1150444	0.002419368
EIF4EBP1	-0.238645729	0.133266128	0.115056109	0.009874433
FANCG	-0.343836013	0.003200677	0.115469043	0.008672786
B3GNT7	-0.437975556	0.058187342	0.116569012	0.003660439
DNASE1L1	-0.169387034	0.007372281	0.11671833	0.012521007
PSMG4	-0.155193638	0.030758978	0.116740737	3.60E-05
FTL	-0.398929434	0.494339665	0.117254225	0.012723744
TMEM14B	-0.100485479	0.098405399	0.117381538	0.000357154
COG8	-0.132027781	0.008301532	0.117490337	0.04924308
CITED2	-0.404518766	0.19638395	0.117502309	0.001462795
LRRC46	-0.267077267	0.004611935	0.117773403	0.000111884
CORO1C	-0.285934639	0.185931652	0.118101768	6.41E-05
NMB	-0.216837398	0.084956526	0.118398041	2.26E-06
PCK2	-0.254557991	0.025995421	0.118702842	0.000512993
FAAP20	-0.246610801	0.06841286	0.119005366	0.000401976
RFK	-0.250702523	0.028563548	0.11903948	0.000795601

LSP1	-0.282087273	0.285524795	0.119134973	2.05E-05
RRP1B	-0.239250789	0.027878097	0.119374453	0.000333555
UBXN10	-0.219557549	0.003619908	0.119386194	0.003023528
CYBRD1	-0.218484866	0.026441587	0.119628759	0.003443511
PLEKHJ1	-0.223115146	0.031761784	0.120460542	0.002529964
UBA5	-0.110404144	0.010969036	0.120806675	0.002212949
REX1BD	-0.291221129	0.187901936	0.120995838	1.74E-06
NDUFA4L2	-0.334834179	0.001510271	0.121104192	0.013997666
CIAO2A	-0.24157133	0.161779081	0.12121083	1.75E-05
MHENCR	-0.460180133	0.005819644	0.121410493	0.029635822
TBCB	-0.277783283	0.071429692	0.121514208	0.001926799
GLRX2	-0.230765781	0.032550999	0.121665762	0.002315004
ANXA5	-0.252310893	0.250103202	0.122394517	0.00109846
MAT2B	-0.185803448	0.029950532	0.123435608	0.005806797
SARNP	-0.021049007	0.125764937	0.123436178	0.016415529
AP5B1	-0.418208199	0.026873408	0.123770412	0.001942125
FXYD3	-0.258783274	0.016496544	0.124528503	8.04E-06
DCK	-0.121597378	0.042278901	0.124671999	4.08E-05
RPA1	-0.171333508	0.060136486	0.125297129	0.002187849
FAAP100	-0.398912737	0.01096213	0.1253928	0.028448543
BTF3L4	-0.11245439	0.051538213	0.125400224	0.022497663
AURKB	-0.323957083	0.006239352	0.125965359	0.002007201
SLC49A3	-0.318370527	0.021846006	0.126140508	0.001466085
GATD1	-0.294548919	0.007713255	0.126233353	0.020548464
MELTF-AS1	-0.233265047	0.001743516	0.12633614	0.001471108

UBXN11	-0.141046534	0.08639232	0.126552183	0.049249616
PET117	-0.055983586	0.019308913	0.127287299	0.00522324
MAK16	-0.228833275	0.009367482	0.128515826	0.00128504
LAMTOR3	-0.157843543	0.051655671	0.128555606	0.00161143
LINC02562	-0.298589294	0.000347026	0.128824985	0.037410619
PDLIM7	-0.365838335	0.046529892	0.128887393	0.024042449
GRHPR	-0.179043674	0.028959072	0.129440319	0.040487076
PRPS1	-0.154019553	0.036366661	0.129615764	8.00E-08
ITGB1BP1	-0.256594694	0.153199004	0.130513294	7.71E-08
SURF6	-0.301773279	0.014963128	0.130725868	0.000898635
GEM	-0.273467015	0.07099118	0.131020211	0.002930536
TPMT	-0.146417046	0.058045103	0.131233459	1.46E-07
ATIC	-0.144893003	0.02453137	0.132131251	0.02853881
GFOD1	-0.091822407	0.135706679	0.132304658	0.009775863
NRGN	-0.296571293	0.034717584	0.133141243	1.46E-05
PDCD2	-0.17475222	0.040853063	0.133381098	0.000470041
CNIH1	-0.078766499	0.121813143	0.134003993	5.51E-07
CENPBD1P1	-0.088635705	0.013859133	0.134462824	0.022887206
MRPS25	-0.152217433	0.034122877	0.134625724	0.029571314
SMIM20	-0.069151836	0.06456151	0.135282893	7.47E-05
SLC35B1	-0.247209204	0.024005073	0.135287557	0.041540949
TNFRSF18	-0.259089946	0.00550207	0.135322583	0.018495909
CTDSPL	-0.069329349	0.02597408	0.135432127	0.000250783
TREM1	-0.243036685	0.173601947	0.135475091	0.003510424
NDUFS3	-0.148376389	0.165161954	0.135763483	0.002502701

GINS2	-0.258486455	0.005793139	0.136418569	2.85E-05
B4GALT2	-0.274632001	0.006203048	0.136479274	4.14E-05
LY6H	-0.482355967	0.000450986	0.136520947	0.000451091
LINC01632	-0.008367702	0.002293198	0.136584668	0.002086696
PLEKHA3P1	-0.508919907	0.00256546	0.137887245	0.010041729
KNSTRN	-0.254685761	0.012701349	0.138267869	3.61E-05
ZBTB20-AS2	-0.06883217	0.000487356	0.139087047	0.047578709
POLD4	-0.299205596	0.127937818	0.139094224	0.001424598
ZMAT2	-0.118906922	0.067644078	0.139707698	4.58E-05
BCKDK	-0.301604068	0.068008567	0.140507119	8.76E-06
SPRY1	-0.212205667	0.077139975	0.140843914	1.37E-06
APH1A	-0.221106744	0.045153645	0.141144644	0.038254857
LEPROT	-0.118942598	0.08643704	0.141258689	0.009398347
C16orf91	-0.357798154	0.011538702	0.141664191	0.002417598
CBLB	-0.0565403	0.306714148	0.141918613	1.94E-08
MRPL42	-0.12571036	0.036902932	0.1421219	0.003649011
SCCPDH	-0.118646083	0.076320255	0.142923481	0.001910447
CBWD6	-0.03300898	0.084743918	0.143113122	0.000238067
DCAF13	-0.237869092	0.033266525	0.143560419	0.003541418
GTPBP3	-0.238199966	0.009011409	0.143800544	0.030363549
GALK1	-0.213802026	0.035065249	0.144005954	0.001298148
ZADH2	-0.250600518	0.015953236	0.144217202	0.020456221
SEC11A	-0.106721467	0.139942871	0.144449745	0.006584247
AKIP1	-0.141700496	0.040374324	0.145045413	1.67E-05
ISCA1P6	-0.918028245	0.000428001	0.145370321	0.044441939

SOCS1	-0.393144673	0.035203194	0.145428021	8.16E-05
FAIM	-0.261301328	0.061667325	0.145486475	4.84E-08
ANXA4	-0.076921629	0.193102972	0.14560486	6.57E-08
USP8P1	-0.494909029	0.000551766	0.145714954	0.01205479
TP53BP2	-0.166938033	0.065150766	0.145772887	0.01713793
GLRX5	-0.191656144	0.042999219	0.146388521	3.49E-06
POP5	-0.260009131	0.024531662	0.146528947	0.008634975
RWDD4P1	-0.429693495	0.004158251	0.146726072	0.026056651
MYC	-0.305264031	0.029784763	0.147361995	0.008960923
CYTL1	-0.328260801	0.038760882	0.147656992	7.71E-08
TP53AIP1	-0.31261873	0.000491067	0.148081749	0.047421208
ALDH3B1	-0.291976194	0.049105689	0.148758369	0.000600995
LPL	-0.187455568	0.514867523	0.149228758	1.91E-11
ITGB3	-0.07317193	0.016263902	0.149307453	0.021638191
CCDC159	-0.207326764	0.012807425	0.149506643	0.026912294
RRP15	-0.209631179	0.024491077	0.151363002	0.041178275
MALSU1	-0.119059723	0.022178534	0.152551851	0.002206511
PLEKHF1	-0.167861222	0.005736428	0.153050683	6.63E-05
WDR24	-0.212947997	0.005335865	0.153411279	0.01343105
SNRPA1	-0.169000507	0.039069441	0.153614615	0.004050353
SLAMF9	-0.333996973	0.025576293	0.153630908	4.07E-06
RBX1	-0.142241965	0.251384287	0.153692221	1.98E-05
DCTN6	-0.11828865	0.043513485	0.153771924	0.002215257
CD164	-0.139660417	0.21365919	0.154390083	0.000638084
TMEM126A	-0.204223425	0.074282566	0.154585423	0.000245596

TXN2	-0.176380136	0.048792117	0.15480976	0.002740794
SNX17	-0.233254962	0.060506797	0.15535798	0.00555385
TREM2	-0.378108359	0.287312563	0.156166012	6.36E-05
MYADM	-0.33984006	0.163275418	0.156436245	0.001053505
SIX1	-0.126298173	0.000873683	0.156821126	0.014711742
ALKAL2	-0.316799255	0.001432703	0.156918821	0.007427415
NUDCD2	-0.242561674	0.0358266	0.157238295	0.013968821
PPIH	-0.26362873	0.019420954	0.157297354	0.017102286
MYL12A	-0.207615562	0.202815155	0.157411186	0.02254053
EMP1	-0.238281205	0.367582775	0.15742981	5.21E-05
PBDC1	-0.265672399	0.042218297	0.157986802	0.000819545
NEK2	-0.162844558	0.00214259	0.158354485	0.005122195
CRLS1	-0.055551759	0.092646997	0.158965665	4.23E-06
ADO	-0.323830021	0.010441238	0.159351441	0.039092796
ZYX	-0.663634631	0.088031714	0.159769359	0.004246778
CAVIN2	-0.297805273	0.007216208	0.159920656	0.000114538
PIK3IP1	-0.146439074	0.067393056	0.160165137	0.000651641
ISCA1P1	-0.862278819	0.00582204	0.160863126	0.038254857
COMMD8	-0.17962846	0.048399763	0.160896058	0.001011994
CENPK	-0.133911648	0.008223506	0.161589749	0.006801829
WDR3	-0.189050872	0.010930145	0.161878366	0.003887388
JKAMP	-0.049440699	0.021466885	0.162044597	0.00038034
IFI27L2	-0.244880569	0.205993724	0.162830987	3.61E-06
MRPL40	-0.206347248	0.048744711	0.163360674	0.010476122
PPM1G	-0.223585635	0.091419829	0.164731174	0.041257199

NCF2	-0.263646018	0.123621017	0.165738432	0.011425021
CCDC34	-0.099580837	0.010086203	0.165764258	4.71E-05
SPINK6	-0.177700623	0.005222987	0.16590542	0.002661353
PCNA	-0.227124679	0.036106703	0.166938162	8.76E-06
RRM2	-0.172845563	0.01622122	0.167075502	8.00E-08
SLC2A8	-0.244910326	0.010414921	0.168272092	0.019303206
C6orf120	-0.219985941	0.013959575	0.16860025	0.000552381
SAP18	-0.155712002	0.154271195	0.169328032	0.007473927
TMEM185B	-0.265323772	0.013497721	0.169364084	2.83E-05
OLIG1	-0.391254107	0.001845508	0.169432886	0.048233378
BAG1	-0.190535179	0.080282431	0.16962833	0.000297247
CSGALNACT2	-0.152671946	0.143162784	0.16966314	0.011649679
TMED3	-0.125766073	0.055276416	0.170414071	0.001014606
MLST8	-0.222907514	0.014515906	0.170645848	0.004106629
MTCH2	-0.181709286	0.054591707	0.17078966	0.012251977
RGCC	-0.321014435	0.704272257	0.170895292	2.29E-09
PRKAR2B	-0.041665256	0.047022388	0.170918194	0.014452187
CHCHD4	-0.282651239	0.014264689	0.171523965	0.000590512
CCNO	-0.319415159	0.001550283	0.172387146	0.001851258
PIEZO1	-0.270913366	0.039976121	0.172599584	0.000111324
RAB4A	-0.046983459	0.067640957	0.173320404	0.002612342
COX7A2L	-0.091911772	0.095677468	0.173389527	0.028046352
LYAR	-0.230171211	0.07071369	0.17433575	0.009634098
DYNLRB2	-0.226831746	0.003728015	0.174714168	0.00210134
TOR1A	-0.172066957	0.01594961	0.17633223	0.043975245

SMIM15	-0.161387817	0.037294963	0.178566785	0.017293536
IRX3	-0.15045395	0.00147484	0.179879003	0.005633242
OAS3	-0.486795677	0.025800205	0.179884116	0.027637931
ZNF639	-0.204981726	0.011141217	0.180534985	0.003534299
GGH	-0.156036246	0.019944786	0.180550993	8.65E-06
ZDHHC4	-0.130649973	0.007032774	0.181084986	0.037619734
ZWILCH	-0.118367563	0.005674371	0.182009924	0.027995586
DNAH5	-0.067392294	0.015602999	0.183112104	0.007627233
RABL6	-0.222553797	0.022293889	0.183127528	0.044940081
SURF1	-0.238187631	0.049879658	0.183204553	0.046977594
TRUB1	-0.052224918	0.005633871	0.184423959	0.025665969
NOC3L	-0.15723208	0.008190137	0.18455355	0.030974541
SLC20A1	-0.298465911	0.097863924	0.18468251	0.000747162
GCA	-0.072216191	0.067965924	0.185359072	0.026456145
ARHGDIB	-0.339077971	0.236307217	0.185981163	0.003505537
SELENOF	-0.129075539	0.199575866	0.186977515	9.02E-07
NKAPD1	-0.132576588	0.014894193	0.187265181	0.002169942
TRIM47	-0.311142856	0.010769193	0.187427366	0.01872058
SLC15A4	-0.120801052	0.041048842	0.187455433	0.02097383
BRCC3	-0.082649305	0.011125259	0.187768081	0.017818016
CISD3	-0.293196772	0.1131352	0.188657079	4.33E-06
GTF3A	-0.267741029	0.200551564	0.189088511	4.89E-06
PRMT2	-0.20551346	0.063582163	0.189134995	0.003092619
ATRAID	-0.111259386	0.103596424	0.189963967	0.000758767
FNDC10	-0.290937888	0.009273452	0.190356215	0.004715803

NTHL1	-0.208963259	0.010025892	0.190587167	0.004944503
TNS1	-0.180844861	0.104531882	0.192069722	5.86E-07
URB1-AS1	-0.299813016	0.0053733	0.192314069	0.011921909
DPH3	-0.183224845	0.069340959	0.192564197	0.000290849
LMF2	-0.289224248	0.021895513	0.193445483	0.001381418
FANCF	-0.136355028	0.00491889	0.193660164	0.01066517
HLA-DPB1	-0.232454325	0.395647106	0.193936687	0.018253569
MKI67	-0.310991553	0.01562912	0.194130376	2.41E-05
ABHD5	-0.105819888	0.191577016	0.194837353	0.016254856
RBPJ	-0.051449699	0.295330535	0.196162705	0.000217063
DMAC2	-0.20392482	0.014995406	0.19690045	0.010756706
CHRAC1	-0.199210176	0.020524796	0.200093701	0.01351409
MRFAP1L1	-0.117841479	0.016595728	0.201037062	0.003108424
RASL11B	-0.24732342	0.001449422	0.202060427	0.02254053
PCSK1N	-0.15604973	0.002341035	0.202223424	0.002267065
TIMM17A	-0.195061329	0.048473592	0.202318119	0.010334762
HLA-DPA1	-0.197843412	0.444348032	0.206786447	0.00555385
NCBP2	-0.149391425	0.027188765	0.206993473	0.009833917
LGALSL	-0.19997066	0.010759291	0.207333925	5.99E-08
DCAF7	-0.18062546	0.06225431	0.207719384	0.019535979
CLTA	-0.129590089	0.102898966	0.208290713	0.016110478
SUMO3	-0.18029809	0.115845072	0.209539336	0.001558232
TMEM250	-0.233264984	0.009313006	0.209558739	0.046745122
SDF4	-0.291361386	0.077350804	0.209775717	4.51E-05
HAUS1	-0.151410359	0.017360414	0.209920419	0.021656085

COPS7A	-0.156330989	0.031166415	0.210073718	5.05E-05
LMNB2	-0.27077495	0.009158912	0.211759228	0.012515714
MRPL13	-0.071509304	0.044022528	0.213473611	0.010058629
PMVK	-0.127670326	0.044139611	0.214141404	0.004280349
SAC3D1	-0.257511693	0.013413657	0.21472652	0.004593577
EID1	-0.108118861	0.101845115	0.214807354	0.00099792
CCL7	-0.356261123	0.070649701	0.216939232	7.78E-05
TAX1BP3	-0.141692128	0.085818609	0.217427459	0.000142104
SH3GLB1	-0.132357392	0.131117404	0.217962303	2.16E-06
MZT1	-0.098171513	0.034727663	0.218497187	0.000134498
ACP1	-0.12645649	0.05724197	0.218872517	0.003087931
KXD1	-0.175366517	0.031257439	0.219373427	0.001666292
MRPL11	-0.139250415	0.059317814	0.221336393	0.001237312
MS4A4A	-0.094576672	0.214577397	0.22140503	0.003063066
MAD2L2	-0.135274307	0.028308862	0.223792867	0.018630391
GTF3C6	-0.230254981	0.140484208	0.224132658	0.00011544
CDIPT	-0.156728025	0.033954073	0.225022586	0.001640016
FABP5P7	-0.628476059	0.559690827	0.226981968	1.03E-07
MIEN1	-0.095502542	0.067084051	0.227140242	0.00474242
DPYSL2	-0.19735745	0.220715574	0.227189894	1.94E-08
MBLAC2	-0.069146318	0.003058284	0.227696093	0.001412176
UBE2I	-0.206804608	0.083458884	0.227748672	0.000583752
RPA3	-0.055410274	0.053225023	0.228213501	0.000181825
MLPH	-0.115987352	0.023542683	0.229287523	0.034102669
NFE2L1	-0.288806219	0.016989561	0.229347115	0.016796398

CFAP97	-0.073969286	0.020392539	0.229561232	0.019897767
CAVIN1	-0.431600778	0.01214448	0.230185798	0.004634973
SPRY2	-0.179180786	0.033186143	0.231576302	0.017657667
RER1	-0.111827335	0.086908833	0.233997733	0.001188522
CAPZA2	-0.093134036	0.096831333	0.234357889	0.009787083
PTMS	-0.289308241	0.200523874	0.235055688	6.31E-06
NDUFAF4	-0.123152665	0.033236149	0.238039762	0.00121258
MGST2	-0.064832034	0.278382725	0.238211939	0.005739756
TUSC1	-0.117498558	0.003324953	0.239088207	0.038908453
FARSB	-0.070298655	0.033991363	0.239779957	0.01496097
WDR36	-0.154184266	0.02097111	0.240148707	0.006375187
INTS10	-0.107769741	0.047819468	0.241074664	2.60E-05
MFSD3	-0.286809854	0.016954325	0.241734107	4.53E-05
SRM	-0.336942378	0.051816557	0.242066827	0.002742665
DGCR6L	-0.131886294	0.081582193	0.242586181	3.47E-05
SNRPC	-0.16792372	0.056515104	0.244127914	0.030650536
IMPACT	-0.064484753	0.026953842	0.244400839	0.002583117
SLC10A3	-0.210374583	0.009320872	0.244774518	0.018960117
SSBP4	-0.178457849	0.034385283	0.244864754	0.000421464
SPARC	-0.394838083	0.184023015	0.245651261	3.47E-07
RALB	-0.106695116	0.043202913	0.245779297	1.08E-05
MRPS28	-0.017482449	0.040998862	0.24616935	0.010759564
MFAP2	-0.290140051	0.000883482	0.246253828	0.025609896
TP53I3	-0.192953463	0.031724874	0.24654706	0.000254929
EIF3B	-0.23737753	0.036702751	0.246725649	0.043137298

GOLIM4	-0.130531896	0.066297674	0.24703093	0.018080286
MRTO4	-0.243604029	0.063684127	0.247811547	7.80E-07
TP53TG1	-0.177833605	0.021919899	0.248851255	0.012360168
NSMCE4A	-0.065521529	0.014403718	0.249597484	0.020152814
MRPL4	-0.181524472	0.021813589	0.249636108	0.003129147
ACE	-0.210245805	0.079236313	0.249770205	0.000284431
MCM6	-0.126555843	0.016705035	0.25062782	0.015682861
TIMP3	-0.206377656	0.051181308	0.251805526	4.90E-07
LBHD2	-0.189137392	0.001318578	0.251833891	0.017170761
MAF1	-0.139981514	0.042904292	0.252677102	0.045816374
LTV1	-0.172350061	0.016803166	0.252958778	0.034718912
WBP4	-0.138335263	0.038084014	0.253215901	0.004487013
MFSD10	-0.170457857	0.047283365	0.254181162	0.002212949
GCFC2	-0.043969229	0.018820404	0.25494305	9.08E-05
COL6A1	-0.501227591	0.032859634	0.255057575	8.31E-06
GLT8D1	-0.13430456	0.015362339	0.255732779	0.001992723
COQ2	-0.16841282	0.030353819	0.256190833	0.041765451
SNAPIN	-0.129361155	0.037608357	0.256731035	0.00119456
PEA15	-0.184775399	0.127643301	0.256876847	4.34E-07
IFIT5	-0.098733076	0.01288079	0.258084506	0.004435153
C17orf58	-0.194494038	0.015844199	0.258109605	0.037139929
FAM98B	-0.100012034	0.011224908	0.25976215	0.010378365
CDC42	-0.076000347	0.185084089	0.260378295	0.019846759
LAMTOR1	-0.12555864	0.131571914	0.260822806	0.032653477
SELENON	-0.25375939	0.056019939	0.261277775	3.11E-06

HYAL2	-0.253890009	0.018719159	0.262230736	0.005182267
NENF	-0.140640972	0.13299895	0.26246351	0.000810486
RNH1	-0.246658332	0.161816538	0.262594388	0.001177469
LINC02601	-0.21138627	0.000567429	0.263793673	0.009776425
NR2C2AP	-0.329075698	0.007574323	0.263996651	0.019262553
ALDH1B1	-0.26256257	0.008752569	0.264749842	2.05E-05
TRAPPC2L	-0.211114365	0.090782245	0.26521839	0.001734773
VDAC3	-0.093705096	0.053118458	0.265419507	0.000309625
PSMA7	-0.190111277	0.288366578	0.266485267	0.000291992
ZMPSTE24	-0.15066907	0.03763628	0.267687733	7.99E-05
CYB5R3	-0.221880039	0.055629887	0.268988055	0.014545124
COPS6	-0.161793326	0.034651915	0.26971349	0.040879934
IGFBP7	-0.092250651	0.023504877	0.269819111	0.047505
FBP1	-0.141584299	0.524137422	0.272703113	2.26E-06
C1orf74	-0.197022471	0.000885289	0.272998747	0.038254857
NDUFB1	-0.187603554	0.162187259	0.275086101	0.021292071
COX18	-0.127840281	0.008723643	0.275508519	0.020141458
POP4	-0.180494738	0.025611388	0.275925119	0.008297307
ATP2A1-AS1	-0.284973069	0.001305804	0.276751041	0.000103325
ZDHHC12	-0.213521243	0.06604088	0.277056199	0.000889993
MAFB	-0.303095845	0.185179294	0.277090732	0.001216819
TIMM50	-0.192332899	0.027660839	0.27720047	0.002215257
UBL4A	-0.190544203	0.013501815	0.277466347	0.006351259
AP2S1	-0.170368074	0.256042432	0.279418587	0.008899456
SMIM10	-0.378072553	0.005402275	0.281466561	0.000142235

SERF1A	-0.013316245	0.014352841	0.281478922	0.042037855
CD74	-0.28022391	0.395212148	0.28275206	0.037259876
MISP3	-0.114447123	0.002115891	0.28379014	0.003443511
PSMG3	-0.119159572	0.022485187	0.2845857	0.010771987
COTL1	-0.232599622	0.282456793	0.284806018	0.000310915
C11orf1	-0.130153561	0.018064022	0.285276612	0.000216555
MORF4L1	-0.047276774	0.113086416	0.285488133	0.006938508
FIS1	-0.17741518	0.174367033	0.288649828	1.70E-05
FAM50A	-0.163114585	0.057112684	0.288708803	0.001551812
MAGOHB	-0.19397665	0.025212117	0.289082686	0.001910851
DCTD	-0.106468576	0.030991578	0.289506583	0.005988111
POLR2K	-0.092878293	0.136144806	0.293750887	3.46E-05
COX8A	-0.184210058	0.282326609	0.294297293	0.001502851
RWDD1	-0.137492779	0.067719058	0.29497693	0.020005808
PAXX	-0.208907713	0.036537573	0.295113395	0.006518002
KCTD3	-0.096227963	0.009409323	0.295312235	0.023843204
AKIRIN2	-0.161662828	0.204620454	0.295898039	0.000217468
ZNHIT2	-0.213787148	0.005648644	0.29598346	0.003678764
TRADD	-0.151244027	0.029232885	0.297929655	0.003750258
COPZ1	-0.051901894	0.074592025	0.299000145	0.000724638
FIBP	-0.230747586	0.074458085	0.299148534	0.000427462
NDUFS8	-0.138800944	0.109202874	0.303214693	0.00169938
TMEM50A	-0.076245111	0.157168633	0.303550733	3.67E-05
SSBP1	-0.126489019	0.091726971	0.306232575	0.018430741
FBXL15	-0.186242934	0.032053333	0.306782376	0.022917145

FABP5	-0.292295349	0.574836377	0.307472163	7.37E-08
SELENOS	-0.097191229	0.08789669	0.308240798	0.002285273
SDHC	-0.070852204	0.045445084	0.310123409	0.033427415
CROT	-0.064551328	0.006933064	0.310721748	0.032475443
COPS9	-0.185285059	0.143626667	0.312522173	0.001053505
PALLD	-0.028720774	0.061687486	0.313376365	0.013909245
GSTO1	-0.193846819	0.223875157	0.314920681	0.007728911
DCST1-AS1	-0.176465298	0.000952157	0.316278338	0.005633651
UXT	-0.137177925	0.083916983	0.316461245	0.026688076
DTYMK	-0.206905964	0.024686348	0.317276237	0.00595198
GALNT12	-0.07936975	0.015671363	0.317774408	0.001734773
TMEM167A	-0.102002879	0.127642402	0.319849906	0.000326087
MAD2L1	-0.212425304	0.008995548	0.320267823	8.29E-08
RETREG1	-0.023380823	0.093875555	0.321172695	6.65E-07
SLTM	-0.1049601	0.034477779	0.321647017	0.006880538
NOP56	-0.221084296	0.024338018	0.32215252	0.033965763
SIGMAR1	-0.149455965	0.014641215	0.323345638	0.009859948
THNSL1	-0.121520723	0.001357698	0.325269403	0.004048742
PLXNA1	-0.201187134	0.018329695	0.326627291	0.001109079
LSM5	-0.201906144	0.116378392	0.328754608	0.000428475
TPM3	-0.186839003	0.21779149	0.329166496	7.00E-06
TEX54	-0.616657614	0.000492713	0.330007064	0.045784782
TRIAP1	-0.145842769	0.037535239	0.331630672	0.000545919
SERF2	-0.08369064	0.479591191	0.331939685	0.000912557
WDR43	-0.112693677	0.060248736	0.332007424	0.001051006

TMEM43	-0.099190096	0.05161177	0.333456046	2.51E-06
DDX21	-0.169078113	0.157973444	0.333939371	0.002932514
SPCS1	-0.117369777	0.163125538	0.334184863	9.95E-05
NDUFB5	-0.12251187	0.103264863	0.335547286	0.003482107
CD63	-0.131088917	0.348018361	0.336429562	0.020440707
ZWINT	-0.224572391	0.015858642	0.336489394	2.29E-09
PKP2	-0.057963458	0.228270783	0.338955247	0.007875742
CSRP1	-0.14031943	0.060169554	0.343162891	7.04E-05
CKS1B	-0.271246203	0.024054968	0.34485838	0.005971316
PAK1IP1	-0.177274656	0.017995869	0.345026164	0.016618037
THAP7	-0.14549039	0.013595255	0.34554638	0.008024102
PWP1	-0.088370929	0.027477416	0.345899102	0.008178453
C4orf48	-0.280362644	0.203832424	0.346423343	0.00120881
HSD17B8	-0.175210374	0.011274332	0.34701253	0.002874705
CINP	-0.091478252	0.021495149	0.347098602	0.0336952
NUF2	-0.042484954	0.004953052	0.347151902	0.010799029
UBE2C	-0.153228943	0.012165774	0.348816032	0.000644161
COX14	-0.114260643	0.072302496	0.349195179	0.049300807
RAB18	-0.066576469	0.052673962	0.349258507	0.000426101
FYTTD1	-0.090211238	0.034622643	0.349434543	0.001317384
UTP18	-0.107312954	0.033029501	0.349498915	0.018944928
MRPL19	-0.079166883	0.024217841	0.350036474	0.003741671
MRPL3	-0.07761643	0.071021477	0.350432883	0.000162228
HCST	-0.206843864	0.24863176	0.352198923	6.36E-05
RNASEH2A	-0.091495794	0.011369366	0.353364867	0.012011136

ZBTB38	-0.105236196	0.041391517	0.355924548	0.027407145
CYSTM1	-0.039102614	0.091346166	0.356057732	0.024042449
C3orf14	-0.129812871	0.011600267	0.356824361	0.041628916
RCN2	-0.072362568	0.028718094	0.356916093	0.005157535
PDHB	-0.07824332	0.023218445	0.359710242	0.006469007
RNF7	-0.123795745	0.089349816	0.359908718	0.010927468
NSMCE1	-0.074069286	0.063762762	0.360372099	7.27E-06
SYT8	-0.157857053	0.004800695	0.360656003	0.000314464
PSMG1	-0.100434676	0.019450974	0.360765608	0.025288863
CTSO	-0.062600642	0.020174403	0.361703172	0.009833917
DUSP1	-0.174694879	0.252935141	0.362591957	0.036092069
SESN1	-0.040868513	0.08545222	0.362887546	0.002859061
TUBB	-0.231937802	0.132439511	0.363172313	0.032231566
NDUFB6	-0.104181878	0.077550037	0.364100546	0.004712693
NME1	-0.186335499	0.090139707	0.366417063	0.000408983
SLIRP	-0.103233216	0.11286821	0.366787001	0.01407463
FAM72C	-0.02275609	0.004977083	0.368104988	0.00119456
TMEM230	-0.080532745	0.125584628	0.368129749	0.000499502
NOB1	-0.09483656	0.013923163	0.369663132	0.024331097
SMIM30	-0.114255143	0.160405993	0.369867081	3.36E-05
CAPNS1	-0.139441878	0.096267534	0.370190149	0.004406973
ASNSD1	-0.059896431	0.018680115	0.370232473	0.027866246
BCAP31	-0.106677899	0.178402713	0.370498317	0.000199267
POMP	-0.149675076	0.296139235	0.371031419	0.000574217
BUB3	-0.131216413	0.031130162	0.371910496	0.004755157

AP2M1	-0.157610844	0.083410743	0.372609793	0.044847036
BIRC5	-0.213853946	0.014684669	0.372792027	7.37E-08
APEX1	-0.13510621	0.082765658	0.373400261	0.002522181
NOLC1	-0.163432165	0.02324742	0.373521696	0.00516783
EEF1A1P11	-0.217373935	0.108545585	0.376142682	0.034731865
HMGN5	-0.020059144	0.008890243	0.377162809	0.022780019
TUSC2	-0.177399499	0.032030015	0.377601653	0.020458332
TUBE1	-0.072397084	0.005540571	0.381528627	0.003391796
IER5L	-0.138638782	0.002955649	0.381879189	0.039725316
DNPH1	-0.155708629	0.084049014	0.385093567	0.000891048
RARRES2	-0.449673037	0.006675238	0.386686024	0.031784945
RAD23A	-0.171385476	0.036673941	0.387469945	0.016469801
DUT	-0.063289264	0.097207402	0.388774565	8.10E-05
SRSF8	-0.162226734	0.031119465	0.39066462	0.000121823
NDUFB2	-0.098070139	0.220808272	0.391148682	0.006806927
COL4A2	-0.130353238	0.056305594	0.391964864	0.000670861
SNRPF	-0.091895062	0.093839544	0.392789743	0.005185397
SLC25A39	-0.089826612	0.061012258	0.393007915	0.000935248
NCEH1	-0.089140123	0.161014349	0.393661657	0.01417252
MRPL36	-0.166349669	0.029050079	0.394889711	0.04975091
SNAPC5	-0.147963262	0.026790425	0.395974846	0.010703039
SNRNP25	-0.295720301	0.035029753	0.396212745	0.000764013
FAM98A	-0.088274628	0.008937977	0.397996364	0.013997666
PDCD10	-0.078159354	0.042597166	0.399823928	0.013451495
NCLN	-0.179030494	0.052817668	0.4011731	0.017811041

MPC2	-0.032154544	0.11420066	0.405002965	5.09E-06
AUP1	-0.15962519	0.058052422	0.405421841	0.009646025
MRPL20	-0.15453305	0.107802468	0.405994687	0.000237723
LYPD2	-0.19657749	0.001733805	0.406645348	0.01085039
PLPP2	-0.117692681	0.003437133	0.408390714	0.005939673
TCEAL4	-0.113233125	0.055089702	0.40872429	0.013175853
PPCS	-0.070712598	0.084542381	0.41099098	0.000247546
TMEM141	-0.12428876	0.053519093	0.416663841	0.003536184
LGALS1	-0.169973562	0.698057166	0.41891092	6.79E-05
HINT2	-0.232105283	0.040978513	0.420611918	0.011478166
ERG28	-0.121794383	0.021837859	0.421462611	1.21E-05
SLC35A5	-0.065123181	0.010786719	0.421534361	0.019990111
COMMD3	-0.138827263	0.034397918	0.423312451	0.003113733
DBI	-0.194817768	0.422350768	0.42502285	7.51E-06
CDC20	-0.202814426	0.006547969	0.425239733	1.37E-06
MOB1A	-0.061710028	0.091445517	0.425785879	0.014544593
COMT	-0.079650539	0.149434332	0.425956173	0.00169938
SERPINB6	-0.067659277	0.078051491	0.427177148	7.32E-05
DAD1	-0.076976472	0.152905261	0.429667563	0.000656117
ACTR1B	-0.154472734	0.018515972	0.432598729	0.000811344
SSRP1	-0.135204154	0.033295448	0.433951034	2.16E-06
COX5A	-0.070030625	0.161001513	0.434031195	0.00646736
POLD2	-0.110494062	0.035102447	0.438203261	7.00E-10
CDK2AP2	-0.163309356	0.030523249	0.43890521	0.019309013
ACTL6A	-0.066672326	0.020917179	0.445188621	4.60E-05

CDKN3	-0.111270678	0.021740343	0.445355694	1.79E-10
HMGA1	-0.226151573	0.075548844	0.446581257	0.024280688
ALDH3A2	-0.060429776	0.033598049	0.447398701	0.000724368
RRP7A	-0.136881671	0.058625668	0.448879409	0.006891823
SUPT16H	-0.112734018	0.020162446	0.449073229	0.045982633
ALG5	-0.060228991	0.040992396	0.449357507	0.007728911
ZNF385A	-0.136474938	0.076764015	0.451474665	4.14E-06
GRSF1	-0.083335173	0.032316856	0.454121388	0.019799212
TIMM8B	-0.190160716	0.18882102	0.455881739	8.26E-05
TSR3	-0.169635087	0.018376264	0.456397119	0.00088896
DRAP1	-0.096671426	0.158791134	0.458173363	0.000139511
SURF2	-0.168868449	0.01980814	0.458658347	0.001910447
PGAM5	-0.088064488	0.0085791	0.458856046	0.009606302
PLPBP	-0.085195819	0.030758997	0.461486782	0.000254929
CAVIN3	-0.146000657	0.023723018	0.463302524	5.49E-05
BAD	-0.051956752	0.016192645	0.468627551	0.02881622
YIPF3	-0.090811576	0.032357394	0.470128625	0.002193513
HES6	-0.139016377	0.00520786	0.471014537	0.015258031
BANF1	-0.12207429	0.124984501	0.473719274	0.000532041
MRPS23	-0.134303779	0.042075651	0.474145513	0.000153109
CENPX	-0.11241315	0.058000735	0.475381719	0.003136465
GTF2A2	-0.118748141	0.06642655	0.475440114	0.018889248
WDR13	-0.077678498	0.024415391	0.475697766	0.011976476
ECM1	-0.162178749	0.031361985	0.476307165	3.72E-05
GGCT	-0.073888581	0.073028299	0.480593704	7.84E-06

SNRPD1	-0.123031393	0.122718377	0.483248614	0.000764673
COA3	-0.064022909	0.089444086	0.483639928	8.67E-05
SNU13	-0.100074001	0.083440096	0.484899267	0.045104641
NIFK	-0.109637657	0.029901732	0.485940215	0.002660775
COL6A2	-0.371851231	0.035512076	0.486329594	7.13E-06
ZFP36L2	-0.230217718	0.275876841	0.490511186	0.001186173
LRRC59	-0.139059691	0.023135832	0.491436369	0.02168669
UQCC3	-0.150764989	0.018181907	0.493122727	0.000265137
CT45A10	-0.024746178	0.000362965	0.493231677	0.04057525
MS4A8	-0.084216147	0.00664721	0.499591782	0.001805038
GPX4	-0.135078003	0.254248398	0.499840464	0.047804669
SMC2	-0.062428082	0.012578024	0.500247687	0.014136686
GLRX3	-0.036941207	0.038619785	0.501676511	0.045766612
MSN	-0.171514167	0.120603862	0.50201626	0.017325353
POSTN	-0.448148967	0.005514328	0.503022562	0.008581607
RAN	-0.131173728	0.262786581	0.504706022	1.55E-06
TRMT112	-0.084062602	0.151590438	0.505087573	0.002785535
MTCP1	-0.010372905	0.021663888	0.506199849	0.013997666
HACD3	-0.067857474	0.020013389	0.506931585	0.028841649
NDUFA13	-0.076029956	0.192739283	0.507424459	0.02168669
ARL4A	-0.157406269	0.139362052	0.509217276	0.049948407
GMNN	-0.090888974	0.01473385	0.510034018	0.002091409
FANCB	-0.039194108	0.003191732	0.512184181	0.023215619
PPP1CC	-0.081148636	0.050455293	0.512362778	0.025825538
HTATIP2	-0.081157404	0.04314641	0.513435898	0.009874433

CHID1	-0.05039421	0.054642461	0.515683586	0.002502701
COL1A2	-0.356250519	0.019108581	0.515798823	0.016904647
LONRF1	-0.064486569	0.127013234	0.518199406	5.51E-07
PRDX6	-0.095930487	0.134705981	0.518280604	5.26E-05
CBR3	-0.056157335	0.001289687	0.518357982	0.006042343
DAZAP1	-0.130209084	0.039002651	0.518446339	0.000405491
NDUFC1	-0.066013555	0.065374273	0.521437802	0.00582192
MRPS12	-0.092475453	0.080354909	0.521905615	0.000261283
DCUN1D5	-0.059834836	0.039092062	0.522847423	0.000123693
C11orf58	-0.031965833	0.083528715	0.529705209	0.018944928
CCDC51	-0.08718646	0.004590693	0.530512949	0.004934686
SOD1	-0.089849104	0.117825754	0.530530607	0.001702771
PLXNB1	-0.115695572	0.003161819	0.531188152	0.000931461
FBXO45	-0.075268788	0.008942842	0.53168438	0.014304052
MRPS15	-0.095725204	0.07493058	0.533337381	0.010171549
SNRPE	-0.085751041	0.143583802	0.533863365	0.000389839
NDUFAF3	-0.073669164	0.127088089	0.533942249	5.45E-05
RSL1D1	-0.098548474	0.073654857	0.534894705	0.037632217
DEPDC1	-0.06843638	0.002606445	0.539381862	0.000127675
UQCRB	-0.065133355	0.238560914	0.544880616	0.012360168
MME	-0.049728057	0.046412113	0.545285023	0.012551874
WDR1	-0.123993918	0.116192547	0.546890227	6.52E-06
CHCHD10	-0.076135105	0.249821753	0.547319992	6.16E-05
MRPS16	-0.057148603	0.022115317	0.550063332	0.033527905
CDCP1	-0.084065712	0.132460071	0.550170257	0.000357154

VPS29	-0.102814166	0.167759403	0.550240179	0.001002468
PPP1R7	-0.048031981	0.03243627	0.552491753	0.033855176
SEC61B	-0.07393217	0.143826826	0.55296374	0.037116355
TSPAN19	-0.055845251	0.002833827	0.553563859	0.004597881
TSN	-0.070473195	0.018308656	0.555455162	0.010650055
CHCHD1	-0.095997949	0.04160328	0.557104889	0.006627044
CEP55	-0.096204757	0.00783642	0.558429688	0.002410077
APMAP	-0.080565776	0.036457049	0.558911538	0.028914453
PSMG2	-0.029154345	0.056442284	0.559615727	0.033120129
TMX1	-0.061280961	0.045120205	0.55982183	0.000426101
TPM1	-0.079472076	0.010300103	0.560878413	0.027856628
RAB11B	-0.117167225	0.036779546	0.561026547	0.00102476
KCTD12	-0.133652191	0.159929035	0.562138017	0.000809253
DHRS7	-0.060997385	0.036294715	0.562144254	0.000509676
FBXW5	-0.130465549	0.053771411	0.562417828	0.000547606
CNBP	-0.052082802	0.181052288	0.562983187	0.000204909
AVPI1	-0.05866461	0.11582314	0.563715518	0.001436889
RRAGA	-0.060234299	0.032686613	0.566331729	0.000220159
TIMM10	-0.149303237	0.066029638	0.567551373	0.00696983
OAZ1	-0.067510096	0.455603005	0.570746062	0.000616696
GPT	-0.098059832	0.003308962	0.574681706	0.007502961
MRPL21	-0.067091884	0.039193885	0.575016606	0.012011136
SLC25A24	-0.031408912	0.040000442	0.575551822	0.021938129
MRPL37	-0.035648526	0.049217511	0.577353409	1.37E-06
UCP2	-0.106370771	0.107065507	0.579360692	0.023122645

REEP5	-0.054354678	0.137431449	0.579900264	0.000124644
PLA2G12A	-0.021281159	0.015483833	0.583384406	6.19E-06
LZIC	-0.059145266	0.033678968	0.5835329	0.000123693
RTN4	-0.075872961	0.154240085	0.584517698	0.00128504
MDH1	-0.067236025	0.071628402	0.585636788	0.034642023
PA2G4	-0.064357835	0.038393942	0.586567333	0.028331381
STEAP1	-0.077533059	0.001705333	0.587031238	0.0028429
RDX	-0.030546813	0.184029141	0.587102148	0.000139834
EIF3J	-0.060673612	0.074945143	0.587352984	0.00169938
EXOC5	-0.046489023	0.029853232	0.589268273	0.006342247
ATP5PF	-0.072650008	0.150490682	0.593230265	0.014304052
MTMR1	-0.027595354	0.023091553	0.593905633	0.038688544
POLE3	-0.076994356	0.025118909	0.597842077	0.000485145
PYM1	-0.038566684	0.041300478	0.598536418	0.044372504
TMEM190	-0.167713122	0.008818807	0.598902449	0.000139511
CALU	-0.113102238	0.130987238	0.602893118	3.74E-06
TIMM9	-0.045709878	0.030219679	0.603296725	0.009579785
MRPS18B	-0.059473363	0.030866189	0.603386536	0.02973819
NAA20	-0.07007222	0.078427355	0.604078181	0.011279647
TFAP2A	-0.05285369	0.000596464	0.604158313	0.016504253
ISOC1	-0.036475315	0.014682619	0.605828535	0.002522181
C12orf29	-0.054123756	0.006313791	0.605974023	0.006144256
SQOR	-0.040588617	0.142862954	0.609371302	1.89E-07
CFL1	-0.084583603	0.432092087	0.6117664	0.000159151
POLR2L	-0.097785898	0.303193854	0.612167669	0.000290849

SPAG7	-0.071886257	0.057978329	0.613607283	0.035445807
NME4	-0.045236372	0.031311928	0.614271648	0.001531152
HNRNPKP5	-0.240310971	0.000428401	0.615264694	0.019590369
SLC44A2	-0.06210634	0.058792947	0.615654952	4.55E-05
DUSP7	-0.079518362	0.012777582	0.619432658	5.42E-05
NT5E	-0.083908796	0.00794218	0.621264062	0.036498285
EMC4	-0.089735403	0.038551999	0.621383738	0.017092393
PJA1	-0.048689184	0.005700405	0.622844484	0.007374893
POLR2E	-0.078000893	0.051014676	0.622999289	0.033120129
HAT1	-0.040467986	0.015166043	0.624282451	0.041182735
ARMC10	-0.043781113	0.040548441	0.625352014	0.000605201
CYGB	-0.068252877	0.013030382	0.625435674	0.000973733
RANBP1	-0.105655441	0.160418141	0.625461259	2.61E-06
TXN	-0.077460795	0.351546087	0.626253312	0.000290849
DYNLT1	-0.073524045	0.099691469	0.626431736	0.005536195
FBXL3	-0.029664432	0.016436437	0.62933427	0.015516507
COMMD2	-0.065564511	0.02155986	0.634537105	0.025864936
MRPL15	-0.087662469	0.047183223	0.635931605	0.001051006
ATP5MC1	-0.09006505	0.130790694	0.636456665	0.003571558
ALDH9A1	-0.04168097	0.026743129	0.63721796	0.016913864
GOT2	-0.07606753	0.02517482	0.63756121	5.81E-06
MPC1	-0.07196116	0.0752192	0.637916978	0.042599805
TOMM22	-0.091963435	0.08338562	0.639175465	0.002926269
EPHX1	-0.052866188	0.034872767	0.64049279	0.02178949
CLEC5A	-0.06053279	0.065648255	0.644008996	0.003027741

YWHAH	-0.091862445	0.204351535	0.644960975	0.004890343
ARF1	-0.084151492	0.134089557	0.646605557	0.000291992
RRAS	-0.067344954	0.020586534	0.647793825	0.01566099
ZNF688	-0.096933737	0.016159705	0.649703853	0.017033023
CWC15	-0.058841978	0.047174984	0.649719117	0.017961717
FN1	-0.208808838	1.112004592	0.649797956	1.79E-10
MRPL57	-0.067880581	0.127474462	0.653180273	0.000202066
ATP5MG	-0.029394664	0.211544063	0.656078272	0.045766612
OCIAD1	-0.035018413	0.056771975	0.658318185	0.018080286
SKP1	-0.033277274	0.109743785	0.660911935	0.031355785
CRIP1P2	-0.313045935	0.016324509	0.661559454	0.048572106
ANP32E	-0.068484851	0.025443539	0.661857433	0.013854136
NDFIP1	-0.032522259	0.065116439	0.662911367	0.002881991
TK1	-0.066435745	0.018149144	0.665287084	3.77E-06
HLA-DRA	-0.06930053	0.511453172	0.665929569	0.004793482
DGCR6	-0.009924134	0.019608654	0.665956214	0.003219426
ANAPC11	-0.041808373	0.127147447	0.666492529	0.007891647
RTL8C	-0.114918574	0.041538099	0.667758687	1.09E-05
UNC5B-AS1	-0.106215831	0.001060181	0.667917735	0.008114463
UFC1	-0.057589959	0.082778884	0.668292866	0.035233218
ATP2B4	-0.052427714	0.142103189	0.668777259	1.94E-08
LINC00891	-0.015655666	0.004678522	0.674811652	0.000292214
TXNDC12	-0.028370365	0.059301802	0.674867983	0.000747162
HNRNPD	-0.086592333	0.047028134	0.67733689	0.017937062
SLC39A6	-0.093166873	0.022815929	0.678622369	0.006197794

ZHX1-C8orf76	-0.003996706	0.020966831	0.679694816	0.028039355
EIF5B	-0.057318786	0.101610201	0.681182402	0.000848811
PRDX1	-0.082213746	0.107622279	0.689276736	0.013069955
TMEM9B	-0.038042238	0.064918091	0.69088885	0.003665296
DGKH	-0.024078717	0.102665635	0.692340453	0.000114711
PSMB5	-0.049627391	0.047455117	0.696095357	0.039958505
BAG5	-0.068293983	0.011943897	0.699186373	0.030361951
PDCD5	-0.083310147	0.044351021	0.699888898	0.007224475
SSR3	-0.055649556	0.15254159	0.702531544	6.17E-05
PRDX3	-0.037510928	0.123946171	0.70393909	0.00013109
HSPBP1	-0.033767541	0.02071712	0.706001734	0.002112798
LAMTOR2	-0.056498399	0.165185943	0.708155226	0.002212949
BLOC1S4	-0.050811777	0.013994987	0.708761776	0.016061567
GMFB	-0.044233976	0.049514826	0.711502698	9.22E-06
SH3BGRL3	-0.083067924	0.642510066	0.714797873	2.51E-06
FAM229B	-0.023527819	0.00679873	0.715245906	8.15E-06
TMEM33	-0.043902554	0.09080484	0.716937332	0.000288528
HMGN2P19	-0.162084615	0.00192421	0.718071057	0.031063811
GLUL	-0.054628523	0.35740966	0.718305219	0.004934686
LAMTOR4	-0.065055505	0.157305138	0.718964067	0.027847601
C1QBP	-0.060691555	0.018001867	0.72115391	0.036071322
PLPP5	-0.042976351	0.01629679	0.722669398	0.033326564
RNF146	-0.020658835	0.034165514	0.723119302	0.005472368
BRIX1	-0.045546465	0.021060273	0.724565145	0.013122759
TIMMDC1	-0.0296085	0.02786591	0.725143852	0.038492577

ACTB	-0.114527464	0.63086984	0.726073974	0.000263227
EIF2S1	-0.048944412	0.040675253	0.727241438	0.003219792
SUMO1	-0.029119446	0.08347118	0.729086366	0.011891803
CDK1	-0.050337065	0.012691458	0.729951373	1.17E-05
VAMP3	-0.030752273	0.068997835	0.732770216	4.87E-05
SELENOT	-0.036454829	0.135503189	0.733634833	0.000448726
NDUFAF8	-0.083002236	0.070602142	0.737751854	0.001980029
ERH	-0.028942535	0.144371141	0.740641302	0.003314582
LAGE3	-0.071429984	0.040884828	0.743360405	0.017102286
DCXR	-0.042998295	0.09053586	0.745830965	0.000188131
SNHG16	-0.028206548	0.026866439	0.748986701	0.010286294
MTPN	-0.029941329	0.063670464	0.750191846	0.014891348
GINM1	-0.021490096	0.024826254	0.751052708	0.035659463
TMEM14A	-0.025353619	0.020122782	0.751763803	3.47E-07
MAP3K6	-0.062942238	0.009828517	0.755820401	0.006939723
FAM104B	-0.023086759	0.016751022	0.757863836	0.014715049
SNX3	-0.039339239	0.223459426	0.759043791	0.002624541
RABGGTB	-0.02643438	0.041928163	0.759387276	0.029864425
SSNA1	-0.051692378	0.064930413	0.765519667	0.004736948
MRPL17	-0.055064454	0.051115148	0.765646261	3.98E-06
PPIAP31	-0.08717868	0.110868104	0.766774275	0.02385172
CCDC75P1	-0.168436615	0.000244233	0.770361308	0.024769609
YDJC	-0.061276138	0.016949541	0.772776284	0.004838645
NDUFB11	-0.043155812	0.11779406	0.772786553	0.042268432
YIF1A	-0.052697675	0.069665575	0.773203527	0.000139152

NDUFAB1	-0.037745638	0.114897223	0.773340798	0.00120881
PUM3	-0.022241261	0.033574231	0.7748033	0.028892675
ATP6V1G1	-0.038155793	0.275905868	0.775177097	0.000105938
NUDT15	-0.061680213	0.011026021	0.777379294	0.016103553
TNKS1BP1	-0.078899707	0.008838269	0.777845228	0.042199722
IL1RN	-0.044092915	0.154935728	0.780240593	0.044236054
YWHAG	-0.059879407	0.088570933	0.781670917	0.006600329
CCNA2	-0.040764649	0.005119719	0.781704664	1.89E-05
TMEM187	-0.02859079	0.008259775	0.787847984	0.029044239
FH	-0.034130188	0.020016338	0.788550939	0.002252197
ACTR3	-0.036634439	0.117137353	0.791728779	0.009157752
PPIL1	-0.03706348	0.010667144	0.792049717	0.00021526
MED14OS	-0.01973332	0.006482645	0.792822781	0.023610645
MRPS35	-0.01615627	0.067382607	0.79291412	0.000121823
PXMP2	-0.017080283	0.004514933	0.799000535	0.018990634
GNAS	-0.042722326	0.162399157	0.799605496	0.010993024
EXOSC4	-0.056520751	0.033623153	0.801278221	0.000931461
NDUFB10	-0.028157305	0.106969687	0.802401718	0.009451161
HNRNPA2B1	-0.038143816	0.10372128	0.803212324	0.035491105
CLDN8	-0.067407739	0.000375201	0.805539824	0.012680594
COX17	-0.031363761	0.18793751	0.807168377	2.86E-05
CENPW	-0.041977794	0.031439216	0.807362225	0.001545749
PRDX4	-0.033074835	0.069721569	0.808379993	7.04E-05
DEK	-0.031214388	0.104436762	0.813974676	6.72E-06
CCNB1	-0.053729272	0.004579628	0.814139585	0.025447888

POP7	-0.049603416	0.031555464	0.814746575	0.000202721
MRPS21	-0.026224573	0.08007527	0.820297695	0.03885912
MCTS1	-0.019293104	0.074403265	0.831349358	2.48E-05
FGD5-AS1	-0.02171037	0.035754852	0.831962346	0.014713193
TMSB4X	-0.022461194	0.708292215	0.834544236	0.000444738
CALM3	-0.034290416	0.24557787	0.835946456	1.09E-05
NRAV	-0.018282386	0.002800816	0.836050675	8.76E-06
MRPL24	-0.029905397	0.053030835	0.838931557	0.000445834
MAPK15	-0.03524187	0.005249337	0.840020532	0.000736814
ARHGEF35	-0.013419305	0.002472355	0.843761235	0.017811041
GNPNAT1	-0.02185084	0.011906513	0.847313089	4.41E-06
MNS1	-0.013171127	0.003507503	0.847344024	1.28E-05
HNRNPAB	-0.035355467	0.083707856	0.848320917	8.55E-08
ZC3H15	-0.023154509	0.072355284	0.84985422	0.002660775
POLDIP2	-0.02562136	0.022627711	0.851104202	0.047993359
RUVBL1	-0.01214597	0.021166851	0.85117986	3.35E-05
DENND4C	-0.016758706	0.17896472	0.854067204	0.043736715
PDLIM5	-0.010501306	0.10384497	0.855380616	0.008779231
PDAP1	-0.027351482	0.037192698	0.859183675	0.00414001
TRIR	-0.018525979	0.1063676	0.863633357	0.026272393
VPS28	-0.023770103	0.099802712	0.866892676	0.003573467
MRPL43	-0.016460936	0.04605659	0.870837341	0.000598081
AGA	-0.020138867	0.013528652	0.871005698	0.029864425
MYL6	-0.031145791	0.385828899	0.872572513	0.007393162
EBLN3P	-0.025744426	0.019423349	0.875951642	0.027263225

C1orf122	-0.022354344	0.085593873	0.878863518	4.24E-05
FAM207A	-0.004719536	0.035126098	0.883149164	0.000533583
LOX	-0.023195889	0.001711553	0.88778981	0.009976805
FAM210B	-0.010772673	0.024001508	0.888113859	0.000724109
PIN1	-0.018329269	0.086127851	0.889826306	3.36E-05
SDHAF1	-0.028453244	0.010464613	0.892588622	0.026974256
BRK1	-0.015595154	0.188433035	0.892987852	0.001022029
LSM4	-0.019443184	0.114770019	0.895010116	0.000355204
PDZD11	-0.023712128	0.027880831	0.895285469	0.003102023
NDUFB3	-0.018070153	0.100498961	0.898666312	0.006759222
PGRMC2	-0.014382658	0.03507774	0.899568175	7.51E-06
GSPT1	-0.018064726	0.086146085	0.899728998	0.003679893
RTRAF	-0.012736775	0.073877246	0.901654549	0.03430673
SPINT1-AS1	-0.013903271	0.005698923	0.901847585	0.000826966
NPM3	-0.024984315	0.044882721	0.902055955	5.12E-05
ABCE1	-0.016984966	0.058247971	0.902076076	3.75E-05
CD164L2	-0.02351701	0.002731535	0.902553688	0.000250783
JOSD2	-0.012354508	0.06047378	0.903040101	0.006938733
PGM2	-0.010879227	0.021845205	0.91460849	0.025516718
INSIG2	-0.007232728	0.02149497	0.915518796	0.018810722
APPL1	-0.010269865	0.048217343	0.91698225	0.000448972
TMEM258	-0.006896983	0.158856217	0.917833748	0.019720243
VPS25	-0.015958442	0.036147177	0.920657163	0.00098365
S100A13	-0.01111253	0.097795236	0.923382906	0.005695774
EIF3I	-0.013466968	0.057706908	0.924757931	0.023820728

TOP2A	-0.016092305	0.013020631	0.926878791	0.000263289
SELENOW	-0.015733858	0.162774642	0.930893696	1.61E-07
ABHD11	-0.015461703	0.011057226	0.931852058	0.015128895
BEX4	-0.014670758	0.070092831	0.933416064	0.001092711
CYC1	-0.014009993	0.06746284	0.934901649	0.015927682
CNIH4	-0.007716386	0.123335237	0.938156313	0.000250514
SUB1	-0.006981267	0.149860405	0.938660495	0.021982299
NIPSNAP3A	-0.010094221	0.030391044	0.938894384	0.046309273
COL3A1	-0.049147458	0.019153747	0.939784375	0.023215619
OSTC	-0.007549303	0.116791799	0.947468881	0.003580343
CHMP5	-0.006418668	0.095646751	0.947615728	2.03E-05
PAICS	-0.008358217	0.027526935	0.95003789	0.00044253
SNRPA	-0.003848937	0.033112854	0.95240025	0.028914214
MRM2	-0.002860883	0.011824732	0.952495951	0.047181727
MRPL52	-0.012858735	0.11932901	0.953241686	0.003852607
DYDC2	-0.00504793	0.001374971	0.954303116	0.04460736
PTTG1	-0.006554833	0.016077482	0.955848118	0.00461917
APRT	-0.006725105	0.199969467	0.956938772	0.000671769
CCDC167	-0.003973208	0.04525481	0.968842381	0.003414132
NUDT8	-0.004572948	0.006736835	0.982145033	2.85E-05
PNPLA4	-0.00116399	0.012763485	0.982935286	0.014489099
JPT2	-0.002790792	0.009806044	0.983188063	0.019584632
CPM	-0.000597389	0.307413057	0.989226624	0.000192969
PAX9	-0.001127057	0.000597135	0.991884735	0.042955037
GABARAPL2	-0.000291323	0.146334927	0.997134062	1.18E-05

PLGRKT	-4.90E-05	0.05125288	0.999434521	1.06E-05

Table S5. **Comparing the effect of miR-33 inhibition in IPF PCLS to IPF lung macrophage Signatures from ScRNAseq.**

The table shows the list of the profibrotic genes (increased in IPF macrophage signature from scRNAseq data), which their expressions were reversed by PNA-33 in IPF PCLS RNAseq. To evaluate miR-33's ability to attenuate the IPF macrophage disease signature, we compared the log fold changes of the IPF macrophage signature from the previously published scRNAseq data (GSE136831) (generated from 32 IPF lungs and 28 controls (17, 18)) with gene expression profile from IPF PCLS either treated or untreated with miR-33 inhibitor. Non-ribosomal genes with an adjusted p-value below 0.5 and absolute log fold change greater than 0.07 were used to represent differentially expressed genes in IPF macrophages.

References.

1. Hanzelmann S, Castelo R, and Guinney J. GSVA: gene set variation analysis for microarray and RNA-seq data. *BMC Bioinformatics.* 2013;14(7.

2. Mannam P, Shinn AS, Srivastava A, Neamu RF, Walker WE, Bohanon M, Merkel J, Kang MJ, Dela Cruz CS, Ahasic AM, et al. MKK3 regulates mitochondrial biogenesis and mitophagy in sepsis-induced lung injury. *Am J Physiol Lung Cell Mol Physiol.* 2014;306(7):L604-19.

3. Bueno M, Zank D, Buendia-Roldan I, Fiedler K, Mays BG, Alvarez D, Sembrat J, Kimball B, Bullock JK, Martin JL, et al. PINK1 attenuates mtDNA release in alveolar epithelial cells and TLR9 mediated profibrotic responses. *PLoS One.* 2019;14(6):e0218003.

4. Mallavia B, Liu F, Lefrancais E, Cleary SJ, Kwaan N, Tian JJ, Magnen M, Sayah DM, Soong A, Chen J, et al. Mitochondrial DNA Stimulates TLR9-Dependent Neutrophil Extracellular Trap Formation in Primary Graft Dysfunction. *Am J Respir Cell Mol Biol.* 2020;62(3):364-72.

5. Barnthaler T, Theiler A, Zabini D, Trautmann S, Stacher-Priehse E, Lanz I, Klepetko W, Sinn K, Flick H, Scheidl S, et al. Inhibiting eicosanoid degradation exerts antifibrotic effects in a pulmonary fibrosis mouse model and human tissue. *The Journal of allergy and clinical immunology.* 2019.

6. Barnthaler T, Maric J, Platzer W, Konya V, Theiler A, Hasenohrl C, Gottschalk B, Trautmann S, Schreiber Y, Graier WF, et al. The Role of PGE2 in Alveolar Epithelial and Lung Microvascular Endothelial Crosstalk. *Sci Rep.* 2017;7(1):7923.

7. Landini G, Martinelli G, and Piccinini F. Colour Deconvolution - stain unmixing in histological imaging. *Bioinformatics.* 2020.

8. Ruifrok AC, and Johnston DA. Quantification of histochemical staining by color deconvolution. *Analytical and quantitative cytology and histology.* 2001;23(4):291-9.

9. Schneider CA, Rasband WS, and Eliceiri KW. NIH Image to ImageJ: 25 years of image analysis. *Nat Methods.* 2012;9(7):671-5.

10. Lee I, Tiwari N, Dunlop MH, Graham M, Liu X, and Rothman JE. Membrane adhesion dictates Golgi stacking and cisternal morphology. *Proc Natl Acad Sci U S A.* 2014;111(5):1849-54.

11. Zhou Z, Kozlowski J, and Schuster DP. Physiologic, biochemical, and imaging characterization of acute lung injury in mice. *Am J Respir Crit Care Med.* 2005;172(3):344-51.

12. Zhang X, Goncalves R, and Mosser DM. The isolation and characterization of murine macrophages. *Curr Protoc Immunol.* 2008;Chapter 14(Unit 14 1.

13. Theiler A, Barnthaler T, Platzer W, Richtig G, Peinhaupt M, Rittchen S, Kargl J, Ulven T, Marsh LM, Marsche G, et al. Butyrate ameliorates allergic airway inflammation by limiting eosinophil trafficking and survival. *The Journal of allergy and clinical immunology.* 2019.

14. Christensen L, Fitzpatrick R, Gildea B, Petersen KH, Hansen HF, Koch T, Egholm M, Buchardt O, Nielsen PE, Coull J, et al. Solid-phase synthesis of peptide nucleic acids. *J Pept Sci.* 1995;1(3):175-83.

15. Malik S, and Bahal R. Investigation of PLGA nanoparticles in conjunction with nuclear localization sequence for enhanced delivery of antimiR phosphorothioates in cancer cells in vitro. *J Nanobiotechnology.* 2019;17(1):57.

16. Reyfman PA, Walter JM, Joshi N, Anekalla KR, McQuattie-Pimentel AC, Chiu S, Fernandez R, Akbarpour M, Chen CI, Ren Z, et al. Single-Cell Transcriptomic Analysis of

Human Lung Provides Insights into the Pathobiology of Pulmonary Fibrosis. *Am J Respir Crit Care Med.* 2019;199(12):1517-36.

17. Adams TS, Schupp JC, Poli S, Ayaub EA, Neumark N, Ahangari F, Chu SG, Raby BA, DeIuliis G, Januszyk M, et al. Single-cell RNA-seq reveals ectopic and aberrant lung-resident cell populations in idiopathic pulmonary fibrosis. *Sci Adv.* 2020;6(28):eaba1983.

18. Morse C, Tabib T, Sembrat J, Buschur KL, Bittar HT, Valenzi E, Jiang Y, Kass DJ, Gibson K, Chen W, et al. Proliferating SPP1/MERTK-expressing macrophages in idiopathic pulmonary fibrosis. *Eur Respir J.* 2019;54(2).
